# Squarephaneic Tetraanhydride: A Conjugated Square‐Shaped Cyclophane for the Synthesis of Porous Organic Materials**

**DOI:** 10.1002/anie.202212623

**Published:** 2022-10-25

**Authors:** Simon Eder, Bowen Ding, Daisy B. Thornton, Darlene Sammut, Andrew J. P. White, Felix Plasser, Ifan E. L. Stephens, Martin Heeney, Stefano Mezzavilla, Florian Glöcklhofer

**Affiliations:** ^1^ Department of Chemistry Imperial College London Molecular Sciences Research Hub London W12 0BZ UK; ^2^ Centre for Processable Electronics Imperial College London Molecular Sciences Research Hub London W12 0BZ UK; ^3^ Department of Materials Imperial College London Royal School of Mines London SW7 2AZ UK; ^4^ The Faraday Institution Harwell Science and Innovation Campus Didcot OX11 0RA UK; ^5^ Department of Chemistry Loughborough University Loughborough LE11 3TU UK

**Keywords:** Aromaticity, Carboxylic Anhydrides, Conjugated Macrocycles, Imides, Organic Materials

## Abstract

Aromatic carboxylic anhydrides are ubiquitous building blocks in organic materials chemistry and have received considerable attention in the synthesis of organic semiconductors, pigments, and battery electrode materials. Here we extend the family of aromatic carboxylic anhydrides with a unique new member, a conjugated cyclophane with four anhydride groups. The cyclophane is obtained in a three‐step synthesis and can be functionalised efficiently, as shown by the conversion into tetraimides and an octacarboxylate. Crystal structures reveal the high degree of porosity achievable with the new building block. Excellent electrochemical properties and reversible reduction to the tetraanions are shown for the imides; NMR and EPR measurements confirm the global aromaticity of the dianions and evidence the global Baird aromaticity of the tetraanions. Considering the short synthesis and unique properties, we expect widespread use of the new building block in the development of organic materials.

## Graphical abstract

A unique new member of the family of aromatic carboxylic anhydride building blocks—a conjugated square‐shaped cyclophane with four anhydride groups—is presented. The building block can be obtained in a three‐step synthesis and functionalised efficiently to give materials with highly interesting properties ranging from high degrees of porosity to excellent electrochemical properties, as shown by the conversion into imides and carboxylates.

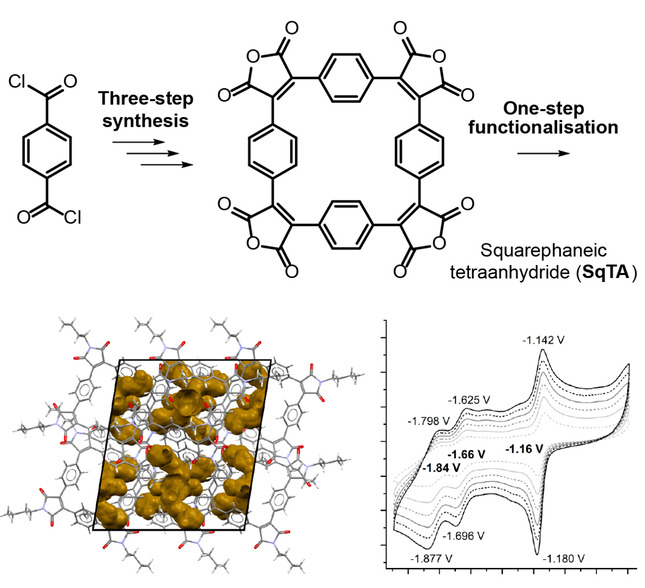

## Introduction

**Scheme 1:**
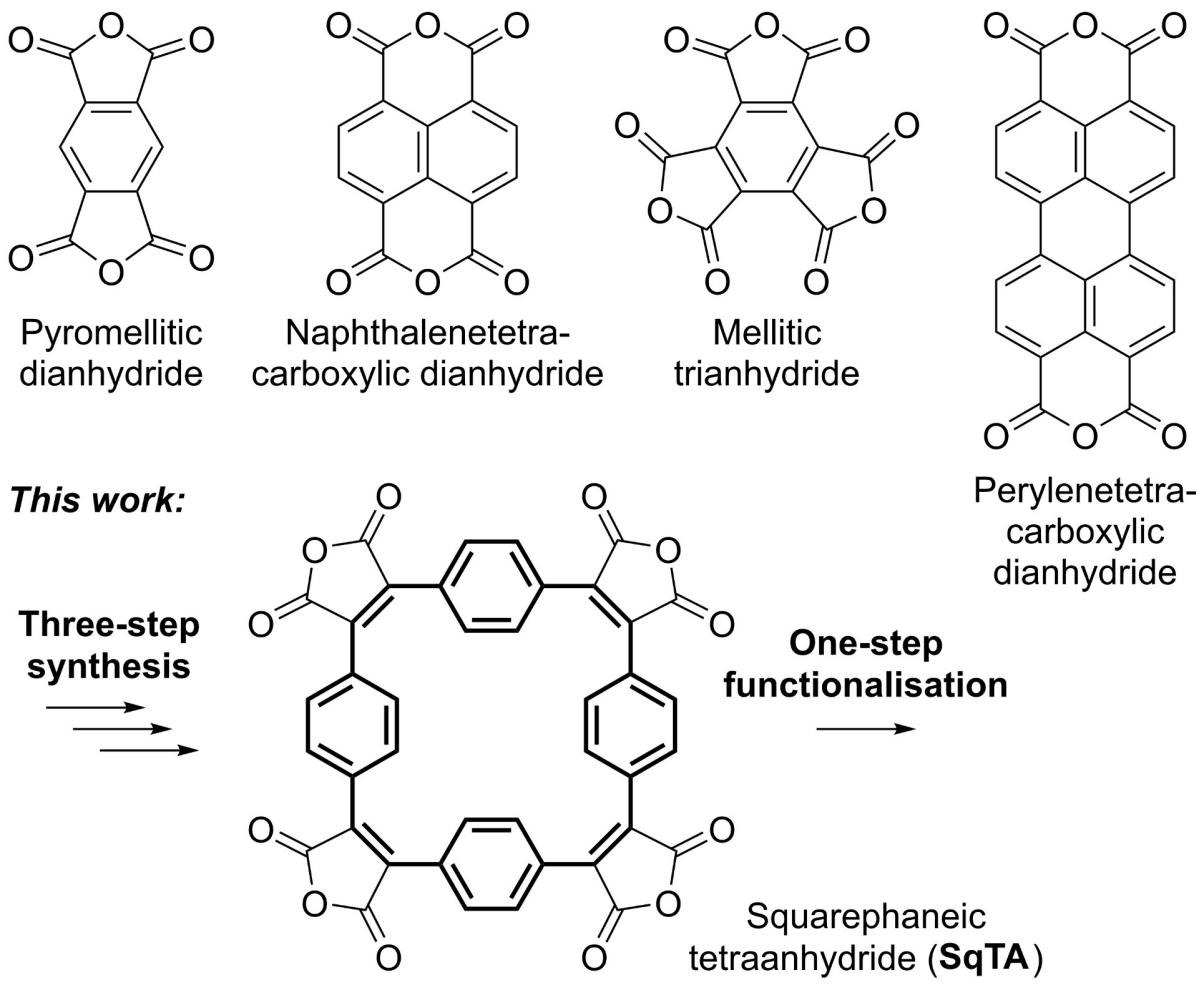
**Top**: Examples of aromatic carboxylic anhydrides, ubiquitous building blocks in the synthesis of organic materials (organic semiconductors, pigments, battery electrode materials, etc.). **Bottom**: Squarephaneic tetraanhydride (**SqTA**) introduced in this work; bold bonds indicate the conjugated paracyclophanetetraene (**PCT**) substructure.

Carboxylic anhydrides are among the most reactive carboxylic acid derivatives available, which makes them highly valuable building blocks for organic synthesis. For the synthesis of organic materials, aromatic carboxylic anhydrides with more than one anhydride group (Scheme 1, top) were found to be particularly useful. Most frequently, these anhydrides are converted into imides: Pyromellitic dianhydride and naphthalenetetracarboxylic dianhydride can, for example, be reacted with diamines to yield polyimides, which—among other applications—can serve as organic battery electrode materials.^[1]^ Both of these dianhydrides can also be converted into diimides, which can either be used directly for applications from biomedicine to electronics or, in case of prior halogenation, as precursors for the synthesis of polymer semiconductors and other functional materials.^[2]^ It has further been shown that naphthalenetetracarboxylic dianhydride can be used for the hydrothermal synthesis of fully conjugated small molecules and polymers, including the industrial organic pigment perinone.^[3]^ Similarly, perylenetetracarboxylic dianhydride can be used for the synthesis of dyes and pigments, namely perylene diimides.^[4]^ Perylene and other rylene diimides have also found widespread use in organic electronics.[^2d^, ^5^]

Importantly, reactions of aromatic carboxylic anhydrides and amines can also yield porous materials, including covalent organic frameworks (COFs),^[6]^ if at least one of the precursors features more than two reactive sites. As a rare example of an aromatic carboxylic anhydride with more than two reactive sites, mellitic trianhydride has been used for the synthesis of COFs.^[7]^ While the porosity can facilitate the insertion/adsorption of ions and gas (an important feature for applications from battery electrodes to gas separation), the functional groups of imide‐based COFs are just as important for their performance, as they provide redox‐active sites and can bind gases and ions.[^6d^, ^6e^, ^6f^, ^8^]

In a different approach to obtaining porous organic materials, we have recently shown that conjugated macrocycles can exhibit a high degree of porosity, as a result of their geometry.^[9]^ In combination with ring current effects that can occur in such cyclic molecules, which can lead to good redox properties, the porosity of the investigated macrocycle paracyclophanetetraene (**PCT**) enabled its application as a high‐performance organic battery electrode material. Other **PCT** derivatives also exhibited good redox properties and unusual ring current effects,^[10]^ further highlighting the interesting properties that can be achieved with **PCT** and its derivatives.

However, for a broader use of **PCT** as a (sub)structure in organic materials, a suitable reactive building block for the synthesis of such materials was missing. Considering the high reactivity and widespread use of aromatic carboxylic anhydrides, we therefore aimed to develop a building block that features **PCT** as an integral component as well as carboxylic anhydride groups for chemical functionalisation. The molecule resulting from this design process, squarephaneic tetraanhydride (**SqTA**) (Scheme 1, bottom; **PCT** substructure highlighted in bold) was expected to enable the facile synthesis of porous organic materials by conversion of the anhydride groups into imides or other functional groups, making **SqTA** a unique new member of the family of aromatic carboxylic anhydride building blocks. Our specific aims were to (i) develop a straightforward synthesis of **SqTA**, (ii) demonstrate its efficient functionalisation by conversion of the anhydride groups into imides and carboxylates, and (iii) show the properties, including the degree of porosity, that can be achieved with **SqTA**.

## Results and Discussion

**Scheme 2:**
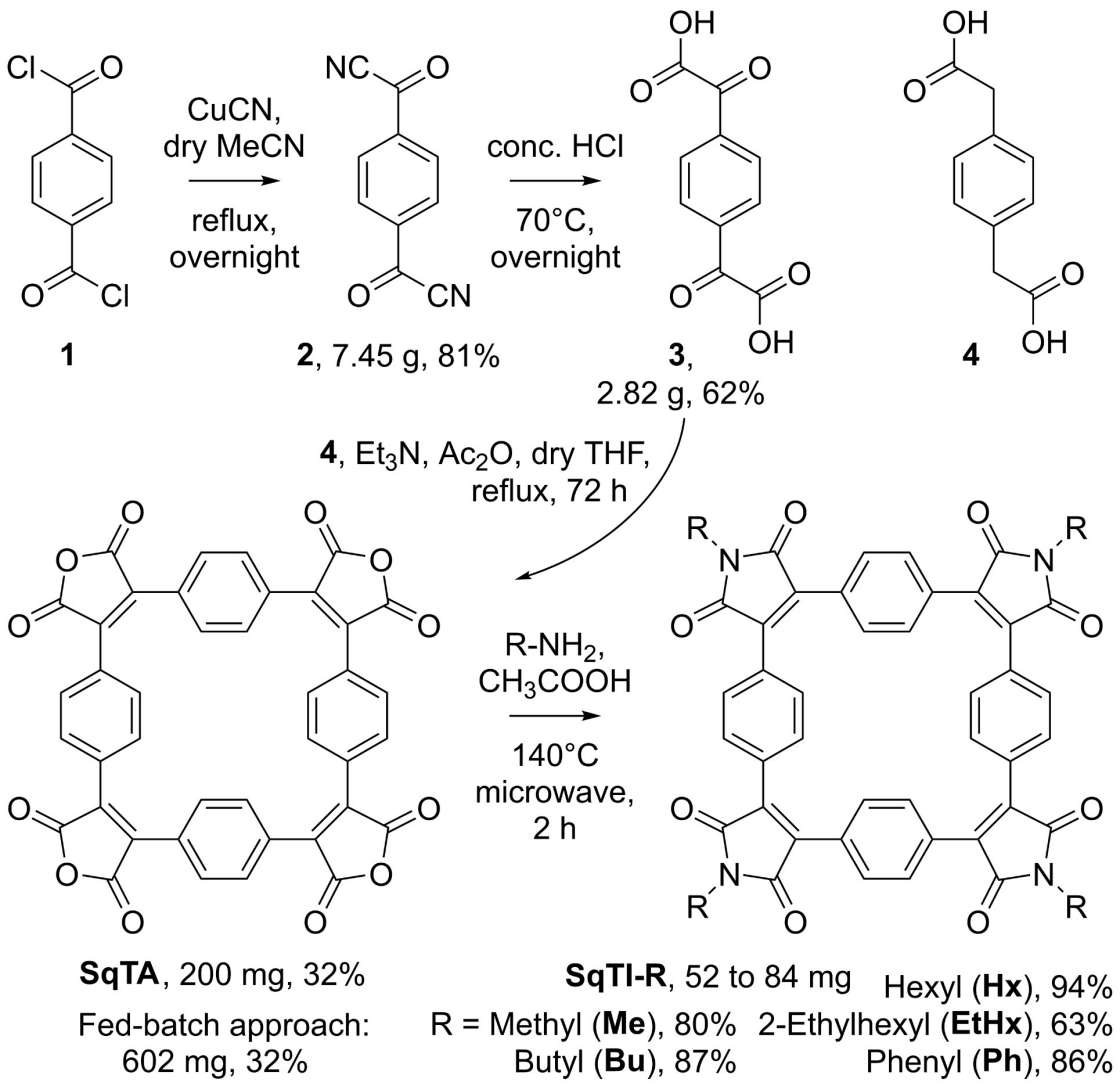
Synthesis of squarephaneic tetraanhydride (**SqTA**) in three steps starting from terephthaloyl chloride (**1**) and conversion into squarephaneic tetraimides (**SqTI‐R**) with different groups R.

For a straightforward synthesis of **SqTA**, we opted for a Perkin‐type cyclisation reaction of α^1^,α^4^‐dioxo‐1,4‐benzenediacetic acid (**3**) and commercially available 1,4‐benzenediacetic acid (**4**) (Scheme 2). Using these two precursors, we recently reported the synthesis of ethyl and hexyl ester‐substituted derivatives of **SqTA**,^[10a]^ which were obtained by in situ conversion of the anhydride groups after Perkin cyclisation, similar to the synthesis of other macrocycles with ester or imide groups.^[11]^ However, for the facile use of **SqTA** as a building block in organic synthesis, the isolated molecule was needed, requiring the development of a suitable isolation procedure. Furthermore, in order to facilitate the synthesis of **SqTA**, we decided to develop a more straightforward synthesis of precursor **3** than used in our previous work, eliminating the need for the highly pyrophoric reagent *tert*‐butyllithium (*t*‐BuLi).

**Figure 1:**
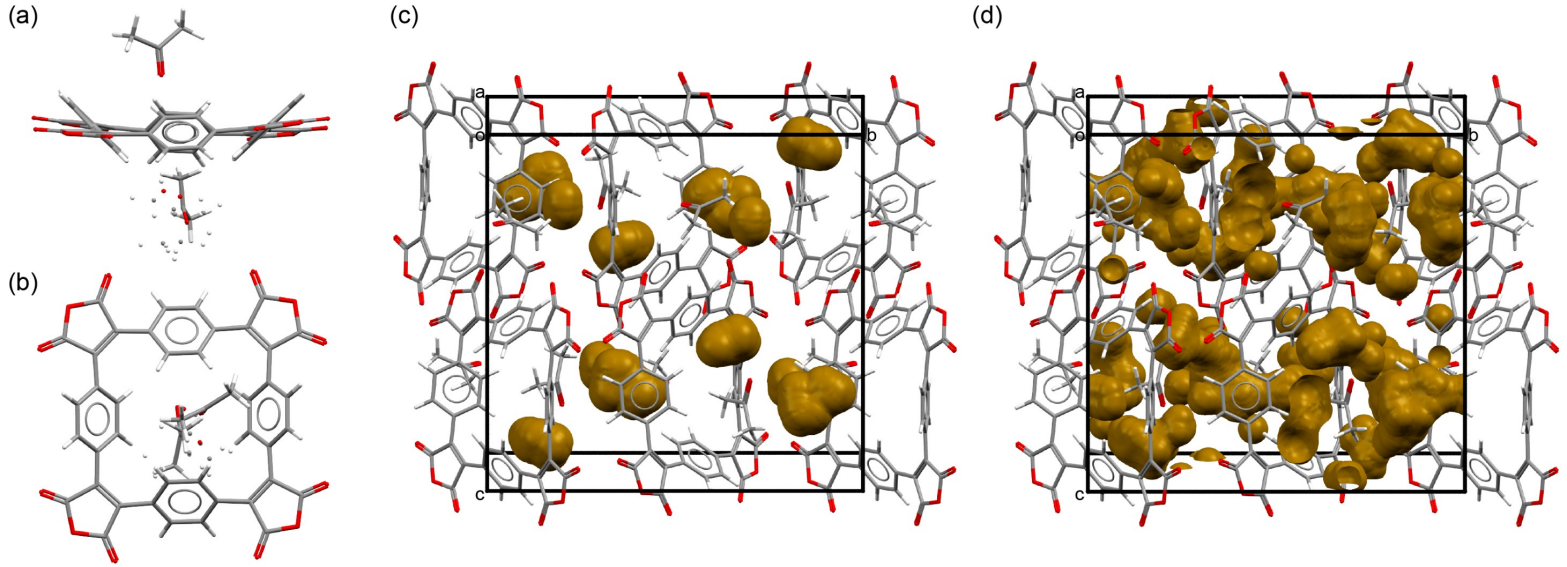
Crystal structure of **SqTA** viewed a) along and b) perpendicular to the least‐square plane of the macrocycle. Unit cell of the **SqTA** crystal showing voids large enough to hold c) sodium ions (3.9 % of the unit cell volume) and d) lithium ions (12.2 % of the unit cell volume).^[23]^

For the improved synthesis of precursor **3**, we used terephthaloyl chloride (**1**) as the starting material (Scheme 2, top) and converted it into terephthaloyl cyanide (**2**) by adapting a previously reported procedure for this reaction.^[12]^ Upon purification of the crude product by sublimation, compound **2** was obtained as a white solid in 81 % yield. Subsequent hydrolysis in concentrated hydrochloric acid (HCl), inspired by the synthesis of the corresponding mono‐substituted compound,^[13]^ gave off‐white precursor **3** in 62 % yield. Purification was again achieved by sublimation, in this case by removing more volatile side products of the reaction. The Perkin‐type cyclisation reaction of precursors **3** and **4** was then carried out under the conditions used for the synthesis of the ester‐substituted derivatives of **SqTA**, but the reaction was stopped at the anhydride stage, omitting the addition of reagents for the further conversion into ester groups. Initial attempts to isolate the product of the reaction by silica gel chromatography, through Soxhlet extraction or recrystallisation did not result in satisfying separation from side products. However, we finally managed to isolate **SqTA** by preparative recycling gel permeation chromatography (GPC) using dimethylformamide (DMF) as the eluent. Using this method, pure **SqTA** was obtained as an orange powder in yields of 32 %. ^1^H NMR and ^13^C NMR spectroscopy, high‐resolution mass spectrometry (HRMS) as well as single crystal X‐ray diffraction (XRD, Figure 1) confirmed its structure.

As with the synthesis of other conjugated macrocycles, our synthesis of **SqTA** in tetrahydrofuran (THF) was carried out under dilute reaction conditions to favour cyclisation over competing oligomerisation or polymerisation reactions. The concentration of each of the precursors in the reaction mixture was approx. 4 mM. To reduce the ensuing high solvent usage and demonstrate the scalability of the cyclisation reaction, we tested a fed‐batch approach in which we added two additional portions of precursors **3** and **4** to the same solution after the initial addition. Remarkably, this allowed us to triple the amount of product while retaining the percent yield (602 mg, 32 %), all without increasing the amount of solvent used (other than the amount of solvent used to dissolve the precursors for the addition).

**Figure 2:**
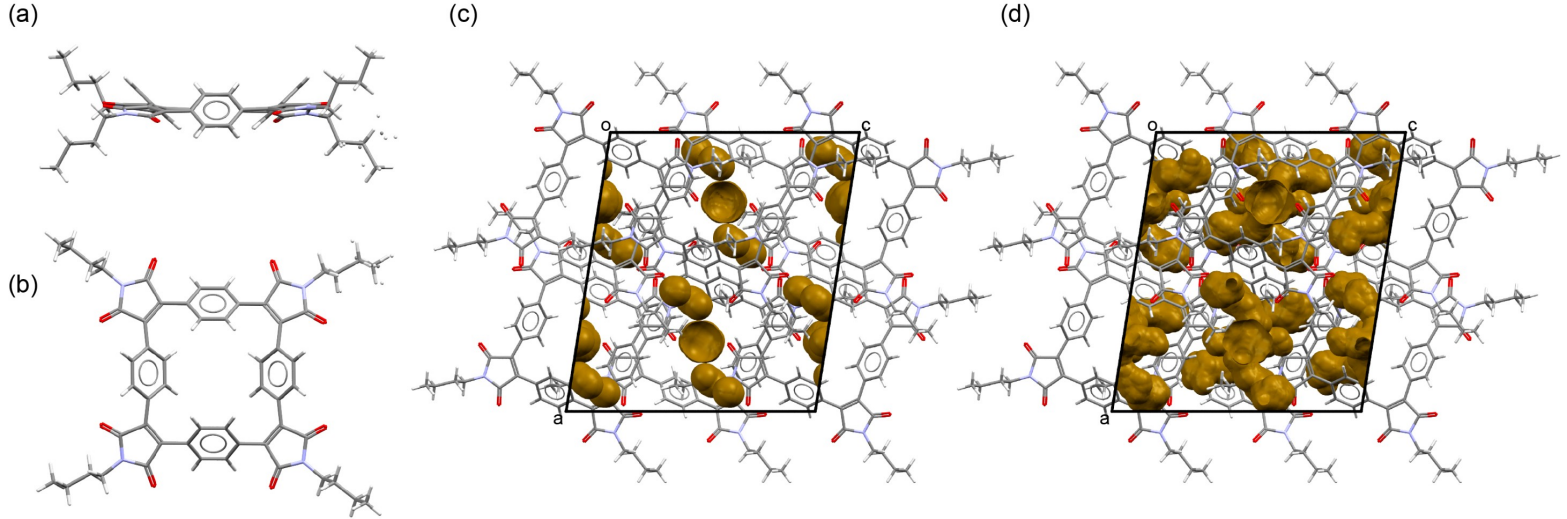
Crystal structure of **SqTI‐Bu** viewed a) along and b) perpendicular to the least‐square plane of the macrocycle. Unit cell of the **SqTI‐Bu** crystal showing voids large enough to hold c) sodium ions (5.4 % of the unit cell volume) and d) lithium ions (12.5 % of the unit cell volume).^[23]^

Further conversion of **SqTA** into tetraimides (Scheme 2, bottom) was first tested using 2,3‐diphenylmaleic anhydride as a model compound and *n*‐butylamine as the nucleophile. Stirring a solution of these two precursors in THF at room temperature for 2 hours and then heating the reaction mixture to 100 °C for 6 hours (in a sealed reaction vial) afforded the corresponding *n*‐butylimide in quantitative yields (see Supporting Information, Section 1.4). Despite these encouraging results, employing the same reaction conditions for the corresponding conversion of **SqTA** into **SqTI‐Bu** did not afford the target molecule. While the initial step of the reaction, the conversion of each of the four anhydride groups into an amide and acid group, appeared to work flawlessly at room temperature, the subsequent ring‐closing step for the formation of the imides at elevated temperature proved to be challenging. Even prolonged heating of 48 hours or the use of a microwave reactor to heat the reaction did not yield **SqTI‐Bu**. However, fortunately, switching to acetic acid as the solvent and heating this solution of **SqTA** and *n*‐butylamine to 140 °C in a microwave reactor for 2 hours did eventually afford the desired product. For the work‐up, the reaction mixture was simply diluted with water, the precipitate was filtered off, washed with water, and dried in vacuo, which afforded pure **SqTI‐Bu** as an orange solid in an excellent yield of 87 %, as confirmed by ^1^H and ^13^C NMR spectroscopy, HRMS, and XRD analysis (Figure 2).

The conversion of **SqTA** into other squarephaneic tetraimides proceeded well under the same reaction conditions. Replacing *n‐*butylamine, used for the synthesis of **SqTI‐Bu**, with other amines, we obtained **SqTI‐Me**, **SqTI‐Hx**, **SqTI‐EtHx**, and **SqTI‐Ph**, which are the corresponding imides with methyl, *n*‐hexyl, 2‐ethylhexyl, and phenyl instead of *n*‐butyl groups. Similarly good yields of 80 to 94 % were achieved, with the exception of **SqTI‐EtHx**, which required purification by silica gel chromatography and was obtained in a yield of 63 %. For comparison, we also tested the synthesis of **SqTI‐Hx** and **SqTI‐EtHx** using crude **SqTA** (not purified by GPC). The final products were then purified by GPC or silica gel chromatography. Over two steps (starting from the cyclisation precursors), purification by GPC (using chloroform as the eluent) gave **SqTI‐Hx** and **SqTI‐EtHx** in overall yields of 24 and 13 %, respectively. This is slightly lower but still comparable to the 30 and 20 % overall yields achieved when using pure **SqTA**. Purification by more easily scalable silica gel chromatography instead of GPC was only tested for **SqTI‐EtHx**, which gave the compound in the same overall yield of 13 %.

**Scheme 3:**
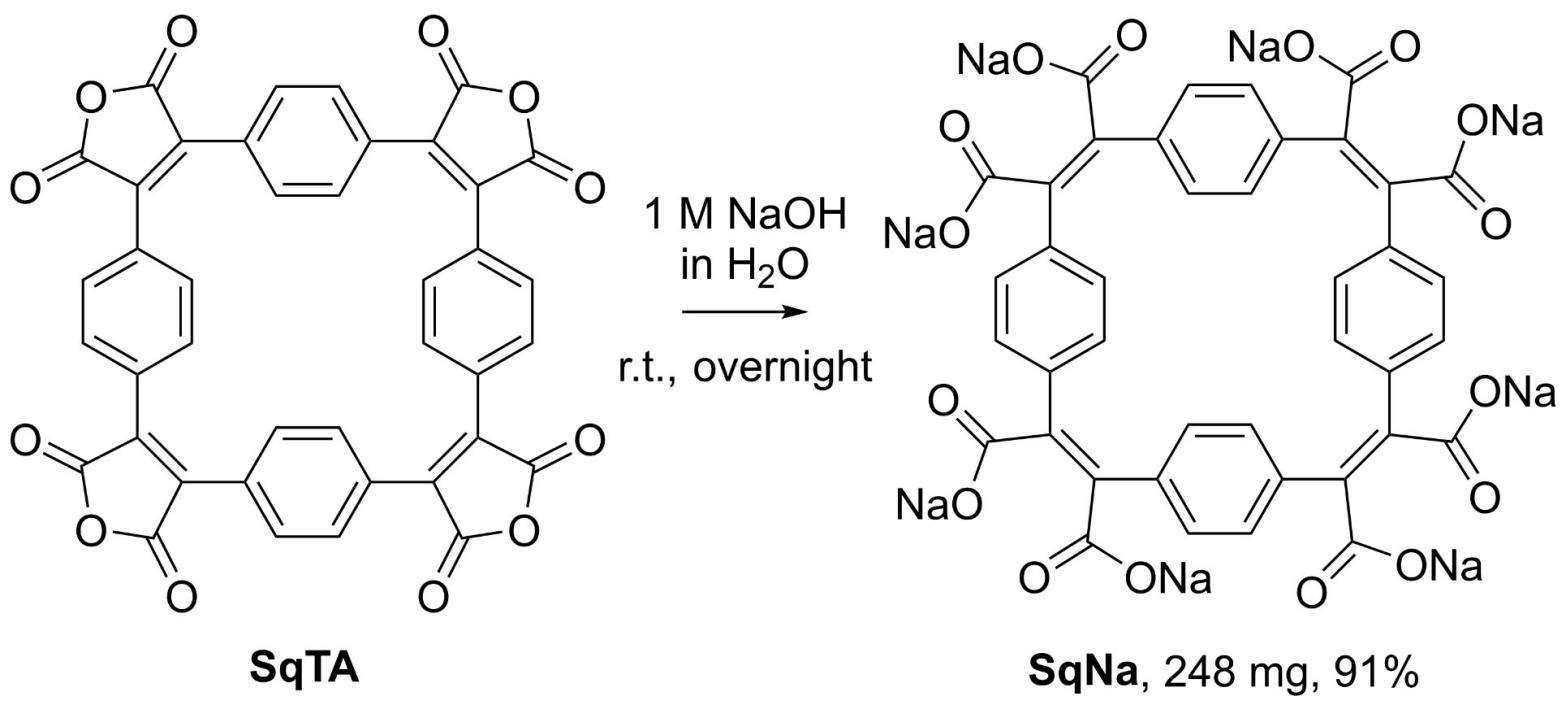
Conversion of squarephaneic tetraanhydride (**SqTA**) into sodium squarephaneate (**SqNa**) in aqueous 1 M NaOH at room temperature (r.t.).

As another conversion of **SqTA** that may yield interesting porous organic materials, we also tested the conversion of **SqTA** into the corresponding octasodium octacarboxylate, named sodium squarephaneate (**SqNa**, Scheme 3). Conjugated compounds with carboxylate groups—like **SqNa**—are frequently used as organic battery anode materials, as their reduction is usually shifted to lower potential (compared to compounds with stronger electron‐withdrawing carbonyl groups such as imides).^[14]^ For the synthesis of **SqNa**, simple stirring of **SqTA** in aqueous 1 M NaOH at room temperature overnight gave a clear solution that was slowly precipitated into vigorously stirred acetone. The precipitate was isolated and washed with acetone for purification, affording **SqNa** as an off‐white solid in an excellent yield of 91 %, as confirmed by ^1^H and ^13^C NMR spectroscopy, IR spectroscopy, elemental analysis, and HRMS.

As shown in our previous work on **PCT**, conjugated macrocycles can exhibit a high degree of porosity, which may facilitate the insertion of counterions (e.g. sodium or lithium ions) into the material (when used as battery electrode materials) whilst avoiding unwanted volume expansion during the insertion process.^[9]^ In order to investigate the degree of porosity that can be achieved using **SqTA** as a building block and to determine the volume of empty space (voids) available for the insertion of lithium or sodium counterions upon electrochemical reduction, we attempted to grow single crystals of the macrocycles for X‐ray diffraction (XRD). For **SqTA** and **SqTI‐Bu**, these attempts were successful. Single crystals of the two compounds suitable for XRD analysis were obtained by slow evaporation of saturated solutions in acetone and chloroform, respectively. The determined crystal structures were then used to perform a void analysis.

The crystal of **SqTA** that was studied showed disordered acetone inclusions (Figure 1a–b). Three different orientations of ca. 52, 28 and 20 % occupancy were identified for the disordered acetone molecules. For performing the void analysis, we assumed full occupancy in the major orientation. Placing a probe of the radius of Na^+^ (1.02 Å) on a regularly spaced grid (0.1 Å spacing) to identify empty space, we estimated that 3.9 % (150.30 Å^3^) of the unit cell volume is empty space large enough to hold sodium ions (Figure 1c), despite the presence of the acetone molecules. This corresponds to a void volume of 37.58 Å^3^ per macrocycle, sufficient space for the insertion of multiple counterions. With a probe of the radius of Li^+^ (0.76 Å), the value increased to 12.2 % (466.36 Å^3^) or 116.59 Å^3^ per macrocycle (Figure 1d). For comparison, we analysed the crystal structure of naphthalenetetracarboxylic dianhydride (CCDC 129443,^[15]^ molecular structure shown in Scheme 1), which features the same ratio of anhydride groups to aromatic rings as **SqTA**. However, using the same method, no empty space large enough to hold sodium or lithium ions was found in this crystal structure.

In contrast to **SqTA**, the crystal of **SqTI‐Bu** that was studied showed no solvent inclusions, but the terminal ethyl portion of some *n*‐butyl groups was found to be disordered (Figure 2a–b); two orientations were identified of ca. 68 and 32 % occupancy. Only the major orientation was considered for the void analysis, which revealed voids of 5.4 % (253.24 Å^3^) of the unit cell volume for Na^+^ (Figure 2c) and 12.5 % (587.96 Å^3^) for Li^+^ (Figure 2d), corresponding to 42.21 Å^3^/97.99 Å^3^ per macrocycle. In this case, we analysed the crystal structure of naphthalenetetracarboxylic diimide (NDI) with two *n*‐butyl groups (CCDC 819749)^[16]^ for comparison. As for the dianhydride, no empty space large enough to hold sodium ions was identified in this structure; only 3.9 % (17.09 Å^3^) of the unit cell volume were found to be empty space large enough to hold lithium ions (Supporting Information, Figure S22), which equals 17.09 Å^3^ per diimide.

The void analysis clearly showed that the reactive anhydride groups in **SqTA** or the imide groups in **SqTI‐Bu** did not affect the porosity; very similar degrees of porosity to those observed for **PCT** were achieved. Reanalysing the most common polymorph of **PCT** (CCDC 1229545^[17]^) using the same software version and method, we identified voids of 5.5 % (255.88 Å^3^) for Na^+^ and 15.2 % (705.46 Å^3^) for Li^+^, corresponding to 31.99 Å^3^/88.18 Å^3^ per macrocycle. The analysis of the structurally related non‐macrocyclic dianhydride and diimide structures further confirmed our previous conclusion that the high porosity is a result of the macrocyclic geometry, which inhibits dense packing.

**Figure 3:**
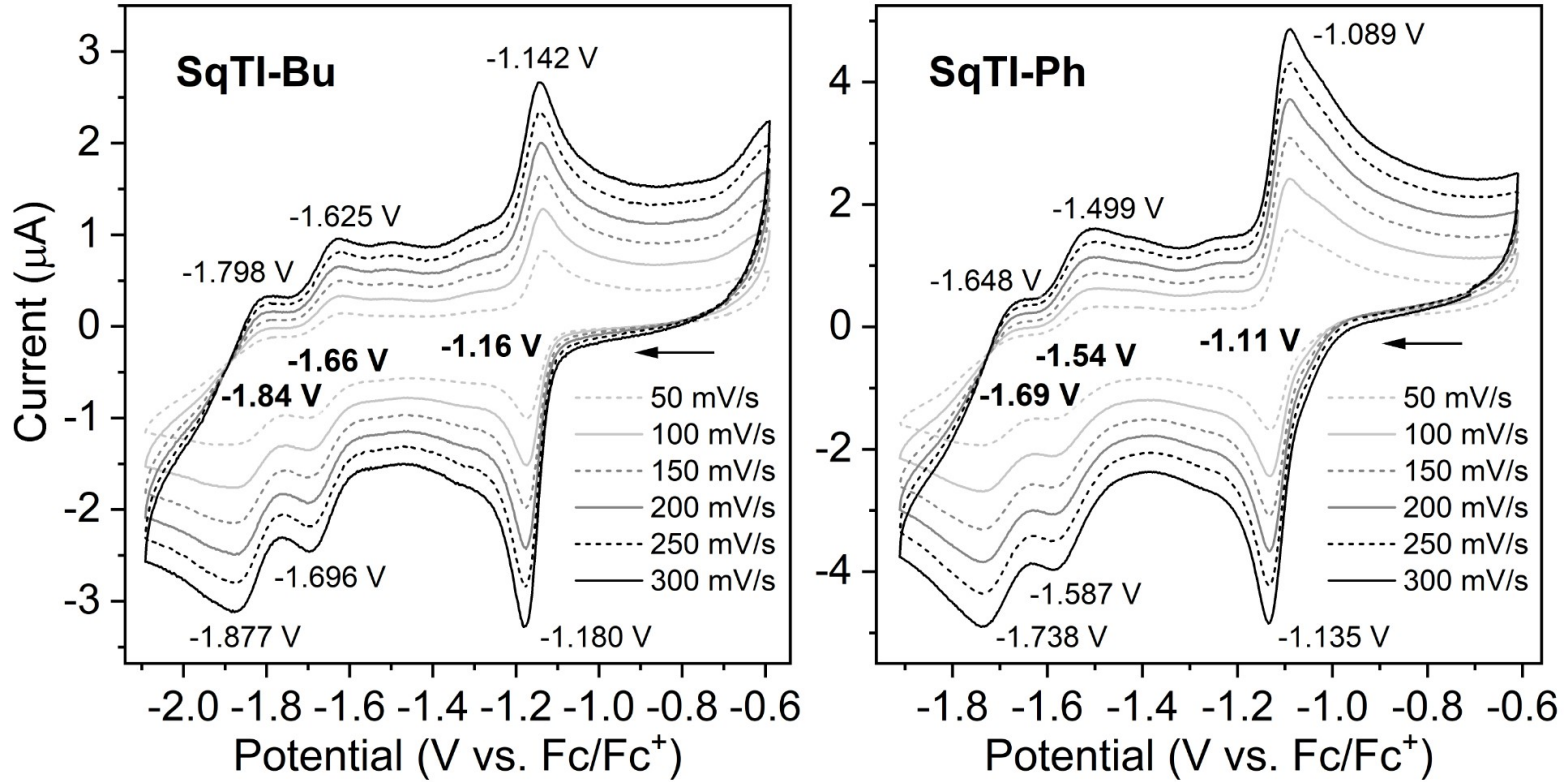
Cyclic voltammograms of **SqTI‐Bu** and **SqTI‐Ph** recorded in 0.1 M [*n*‐Bu_4_N]PF_6_/DMF at different scan rates. A three‐electrode setup was used, featuring a glassy carbon working electrode, an Ag/Ag^+^ non‐aqueous reference electrode and a Pt counter electrode.

Considering that the voids provide space for the insertion of multiple sodium or lithium counterions per macrocycle, we were curious whether our target compounds can undergo the multiple electrochemical reductions that would necessitate such insertion. Indeed, cyclic voltammetry (CV) measurements in 0.1 M [*n*‐Bu_4_N]PF_6_/DMF showed multiple reversible reduction steps for all five squarephaneic tetraimides **SqTI‐R** (Figure 3 and Supporting Information, Figures S23–S27). For the first reduction wave, the difference between the cathodic and anodic peak potential was found to be below the thermodynamic limit for a one‐electron process (57 mV at 25 °C) for all the tetraimides (Supporting Information, Table S1; 38 mV for **SqTI‐Bu** and 46 mV for **SqTI‐Ph** in Figure 3), indicating that the first reduction wave is a two‐electron process. This corresponds well with the previously observed two‐electron reduction of **PCT**, which transitions from a locally to a globally aromatic state upon twofold reduction (but does not undergo any further reductions).^[9]^ For the second and third reduction waves of the tetraimides, the difference between the anodic and cathodic peak potentials approximately doubled compared to the first wave; these are considered to be one‐electron processes. This assignment as two‐ and one‐electron processes also agrees well with the computed redox potentials (Table S2) and with EPR spectroelectrochemical measurements of **SqTI‐Hx** (Figure 5).

The two‐electron reduction of the squarephaneic tetraimides was distinctly shifted to higher potential compared to **PCT**, from −2.15 V vs. ferrocene/ferrocene^+^ (Fc/Fc^+^) reported for **PCT** to −1.16 V for **SqTI‐Bu** and −1.11 V for **SqTI‐Ph**, reflecting the effect of the electron‐withdrawing imide groups. The further reduction steps to the tri‐ and tetraanions then occurred at −1.66 and −1.84 V for **SqTI‐Bu** and at slightly higher potentials of −1.54 and −1.69 V for **SqTI‐Ph** (Figure 3). The redox potentials of **SqTI‐Me**, **SqTI‐Hx**, and **SqTI‐EtHx** were almost identical (±0.01 V) to those of **SqTI‐Bu** (Supporting Information, Figures S23 and S25–S26), with the exception of the reduction to the tri‐ and tetraanion of **SqTI‐Me**, which were shifted to slightly higher potentials. In contrast to **PCT**, for which a two‐electron oxidation at 0.75 V was reported, no oxidation of the tetraimides was observed within the electrochemical window of the electrolyte solution. However, notably, two further reduction waves were observed at very negative potentials of −2.28 and −2.46 V for **SqTI‐Ph**, −2.35 and −2.52 V for **SqTI‐Me**, and approx. −2.45 and −2.60 V for the other squarephaneic tetraimides (Supporting Information, Figures S28).

In contrast to the measurements of the imides, CV measurements of a saturated solution of **SqNa** in 0.1 M [*n*‐Bu_4_N]PF_6_/DMF did not show a reduction wave attributable to the compound, probably due the extremely low solubility of the compound in DMF (as in any commonly used solvent other than water). Measurements in KCl/water also showed no reduction wave within the electrochemical window, which is consistent with the very negative redox potential indicated by computations (see below).

Considering the excellent electrochemical properties of the imides in solution and the high porosity of **SqTI‐Bu** in the solid state, we were curious to investigate its solid‐state electrochemical activity as an anode in lithium‐ion battery half cells. Therefore, electrodes made of 50 wt % **SqTI‐Bu** as the active material, 30 wt % carbon black, and 20 wt % polyvinylidene fluoride coated onto a copper current collector were cycled against lithium metal in coin cells with 1 M LiPF_6_ in ethylene carbonate (EC)/ethyl methyl carbonate (EMC) (3 : 7) electrolyte (Figure S29). In the anodic scan of the first cycle, two sharp redox peaks were observed at approximately 1.8 and 1.4 V vs. Li/Li^+^, which likely correspond to redox processes associated with the intercalation of lithium ions into the voids of **SqTI‐Bu**. The presence and position of the peaks are in good agreement with the findings of the CV measurements in solution (Figure 3, left), corroborating the ability of **SqTI‐Bu** to intercalate lithium ions. The corresponding deintercalation peaks could also be observed, confirming the reversibility of the redox processes. However, the current of the deintercalation peaks was lower than the current of the intercalation peaks, which we attribute to partial dissolution of the active material in the electrolyte and migration through the separator (see Supporting Information, Section 4.2 for details). From the first scan in our galvanostatic measurements (Supporting Information, Figure S31), a specific capacity of approx. 225 mAh g^−1^ is estimated for the potential range from 2.5 to 1.2 V vs. Li/Li^+^ (excluding the contribution from carbon black at lower potential). However, this capacity is not maintained in subsequent cycles due to the dissolution of the active material.

**Figure 4:**
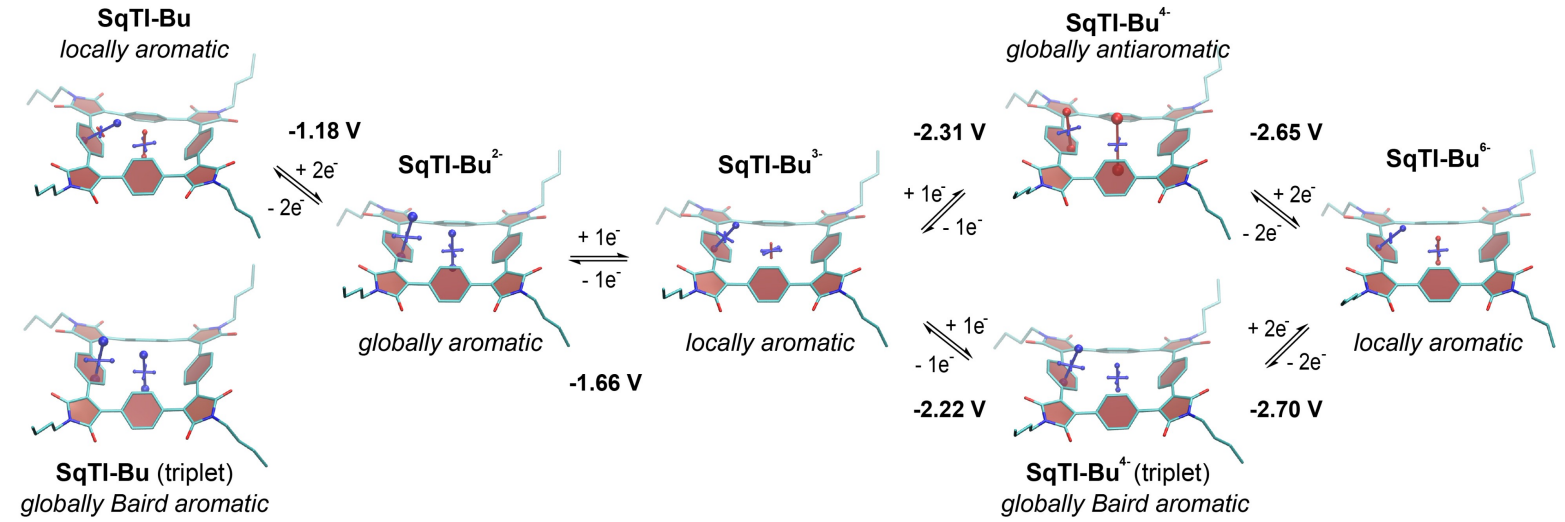
Computational analysis of **SqTI‐Bu**: (i) Computed redox potentials for the different reduction steps in DMF and (ii) VIST plots in the neutral and reduced states showing the aromaticity switching upon reduction and excitation. Shielded (aromatic) tensor components are shown in blue, deshielded (antiaromatic) tensor components in red. Each tensor component relates to ring currents in a plane perpendicular to it. Hydrogen atoms omitted for clarity.^[24]^

To investigate whether the excellent redox properties of **SqTI‐R** can be explained by ring current effects in the macrocycles and, more generally, to assess the local and global (anti)aromaticity in these systems, we carried out nucleus‐independent chemical shift (NICS)^[18]^ calculations for **SqTI‐Bu** in the neutral (S_0_ and T_1_) and reduced states. The NICS tensors were then represented graphically using the visualisation of chemical shielding tensors (VIST) method (Figure 4).^[19]^ VIST shows the tensor components as blue (shielded, aromatic) or red (deshielded, antiaromatic) dumbbells. As for the macrocycles in our previous work,^[10]^ two different positions were investigated: 1 Å off the plane of a phenylene unit, denoted NICS(1)_ph_ here, and at the centre of the macrocycle, denoted NICS(0). The NICS(1)_ph_ tensor provides information about the local aromaticity of the phenylene units, while the NICS(0) tensor is best suited to indicate the presence of macrocyclic currents.

As each tensor component in the VIST plots relates to ring currents in a plane perpendicular to it, the large blue NICS(1)_ph_ tensor component in the neutral S_0_ state indicates that the macrocycle is dominated by the local aromaticity of the phenylene units in this state. However, in the doubly reduced state, **SqTI‐Bu** becomes globally aromatic according to the computations. Such switching between different aromatic states can explain the observed excellent redox properties, due to the stabilising effect of the aromaticity.^[9]^ In order to experimentally confirm this finding, we performed low‐temperature ^1^H NMR measurements of **SqTI‐Hx** in the neutral and doubly reduced state. **SqTI‐Hx** was used for these experiments as the hexyl chains ensured that the compound stayed in solution upon chemical reduction and cooling to 193 K. Cobaltocene in CD_2_Cl_2_ was used as the reductant, inspired by a recent work on structurally related compounds,^[20]^ as its reduction potential lies conveniently between the potentials of the first and second reduction wave of the tetraimides. Indeed, the NMR measurements showed a transition from a locally to a globally aromatic state upon reduction to the dianion. The signal of the phenylene‐Hs at 7.65 ppm splits into two signals upon reduction, a signal at 9.22 ppm for the external Hs and another signal at −0.03 ppm for the internal Hs (Supporting Information, Figures S35, S37 and S38), indicating the presence of a macrocyclic diatropic current. This is further confirmed by the downfield shift of the aliphatic signals of the hexyl chains (the shift gradually decreases from 0.40 ppm for the CH_2_ closest to the conjugated system to 0.04 ppm for the CH_3_ terminating the hexyl chains).

For the trianion of **SqTI‐Bu**, the computations again indicate local aromaticity, while the tetraanion shows strong global antiaromaticity (Figure 4). As this was somewhat surprising considering the high reversibility of the reduction to the tetraanion in the CV measurements (in DMF) as well as the negative effect on the stability that such antiaromaticity would have, we also studied the triplet state of the tetraanion. Interestingly, these computations indicated that the tetraanion of **SqTI‐Bu** has a triplet ground state that is dominated by global Baird aromaticity, explaining the reversibility of the reduction. To further corroborate this finding, we computed the redox potentials for the different reduction steps in DMF (the values are provided in Figure 4). Excellent agreement is found for the first two reduction waves whereas the potential for the reduction to the tetraanion comes out as somewhat too negative. Importantly, the computations indicate that the Baird aromatic triplet of the tetraanion is favoured with respect to the antiaromatic singlet, producing a better fit with the experimental redox potential.

**Figure 5:**
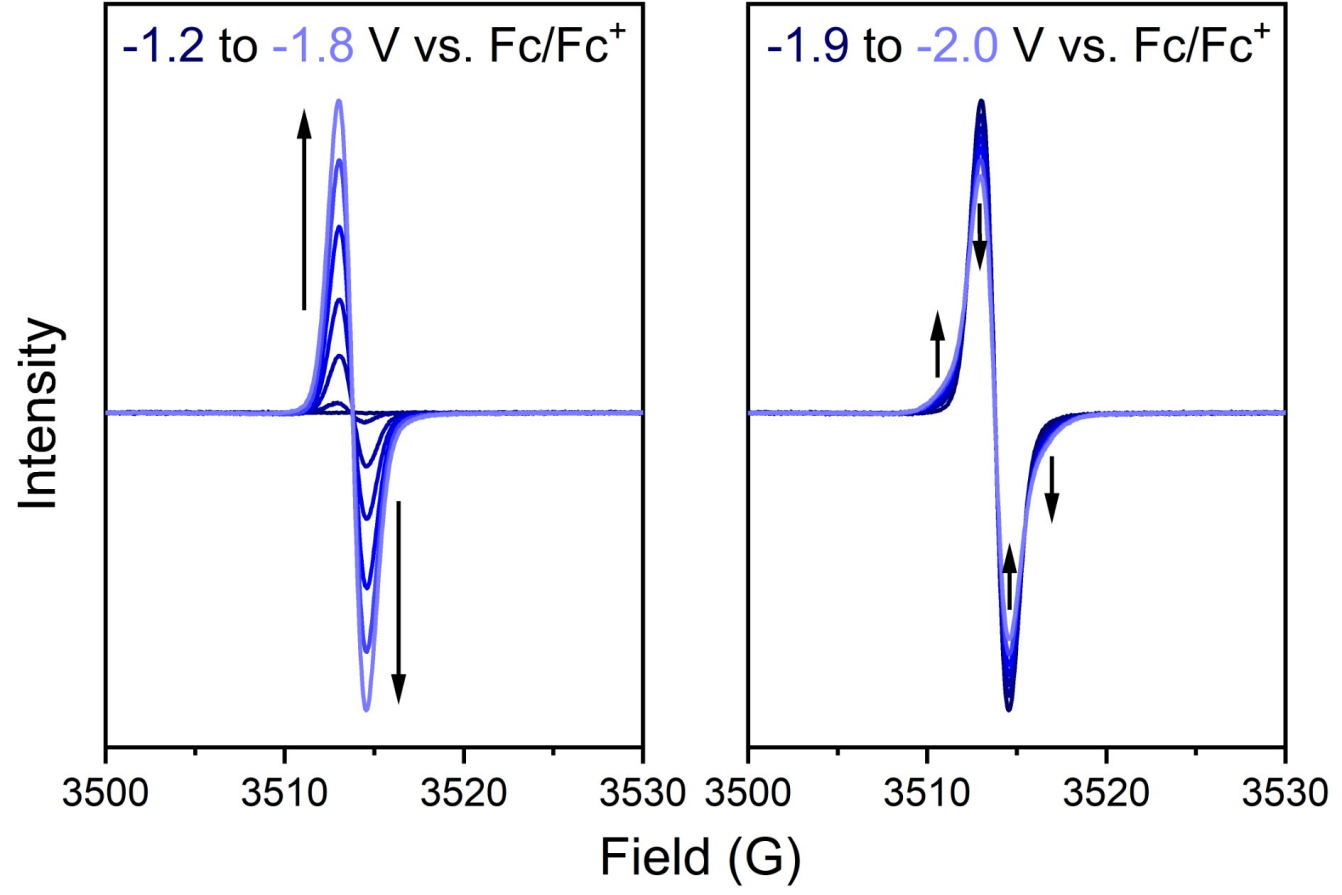
EPR spectroelectrochemical measurements of **SqTI‐Hx** in 0.1 M [*n*‐Bu_4_N]PF_6_/DMF showing the emergence of a signal when incrementing the applied potential from −1.2 to −1.8 V (left) and broadening of the signal when further incrementing and holding the potential at −2.0 V (right).

To obtain experimental evidence for this Baird aromatic triplet ground state of the tetraanion, we probed the spin multiplicities of the three discrete redox states of **SqTI‐Hx** by solution state electron paramagnetic resonance (EPR) spectroelectrochemistry (SEC) in 0.1 M [*n*‐Bu_4_N]PF_6_/DMF. **SqTI‐Hx** features the same redox potentials as **SqTI‐Bu** (Supporting Information, Figures S25), but the increased length of the alkyl chains was expected to benefit the solubility in the reduced state (as for the NMR measurements of its dianion). Application of a cathodic potential up to −1.2 V vs. Fc/Fc^+^ to access the first discrete redox state at −1.16 V resulted in an EPR silent species, confirming its assignment to the singlet dianion state. As the applied potential was further incremented from −1.2 to −1.8 V, an organic radical signal centred about *g*=2.0067 was observed to emerge and intensify (Figure 5, left), which was attributed to accessing the doublet trianion state of **SqTI‐Hx**. Upon equilibration of the organic radical signal at −1.8 V, the applied potential was further incremented to and held at −2.0 V, to access the third discrete redox state of **SqTI‐Hx** at −1.84 V, resulting in an overall broadening of the organic radical signal about *g*=2.0067 (Figure 5, right). The EPR active nature of this tetraanion state and the structural changes to the EPR signal upon conversion provide evidence for its triplet nature. The stability and reversibility of this state was confirmed upon returning the applied potential to −1.7 V, resulting in a full recovery of the doublet trianion organic radical signal (Supporting Information, Figure S33). As the applied potential was further incremented back to 0 V, a slow decay of the doublet radical signal was observed (Supporting Information, Figure S34), indicating excellent stability of the radical, which has also been observed in related motifs such as NDI.^[21]^

Using sodium naphthalenide as a stronger reductant than cobaltocene to access the tetraanion of **SqTI‐Hx** for ^1^H NMR experiments in THF‐d8, the same effects as with cobaltocene were observed (Supporting Information section 7.2). The recorded NMR signals likely correspond to the dianion, with the further reduced paramagnetic species being NMR silent.

A computational investigation of **SqTI‐Ph** and **SqTA** showed the same effects as observed for **SqTI‐Bu** with the only exception that the trianion was predicted to experience more pronounced global antiaromaticity in these systems (Supporting Information, Tables S2 and S4 and Sections 5.4.1 to 5.4.3). In contrast, global ring currents play no role in **SqNa**; all investigated states are dominated by local aromaticity according to the computations (Supporting Information, Section 5.4.4). The computations also indicated a very negative redox potential of −2.33 V vs. Fc/Fc^+^ for the first reduction of **SqNa**, explaining why the reduction was not observed in the CV measurements in KCl/water.

In order to represent the location of the electrons added in the reduction process, we then computed electronic attachment densities^[22]^ for the di‐ and tetraanions of **SqTI‐Bu** and **SqTI‐Ph** (Supporting Information, Section 5.5). In both macrocycles, the attached electrons are concentrated around the five‐membered imide rings with dominant contributions around the carbon atoms of the formal C=C bonds that form part of the macrocyclic conjugated system. This explains the strong changes in macrocyclic ring currents observed in Figure 4 upon attachment of additional electrons. As a characteristic feature, we find that the imide rings maintain the planarity of the link between the phenylene units, thus, enhancing conjugation. Conversely, enhanced twisting around the linking vinylene units explained reduced ring currents in related previously studied systems.^[10]^

**Figure 6:**
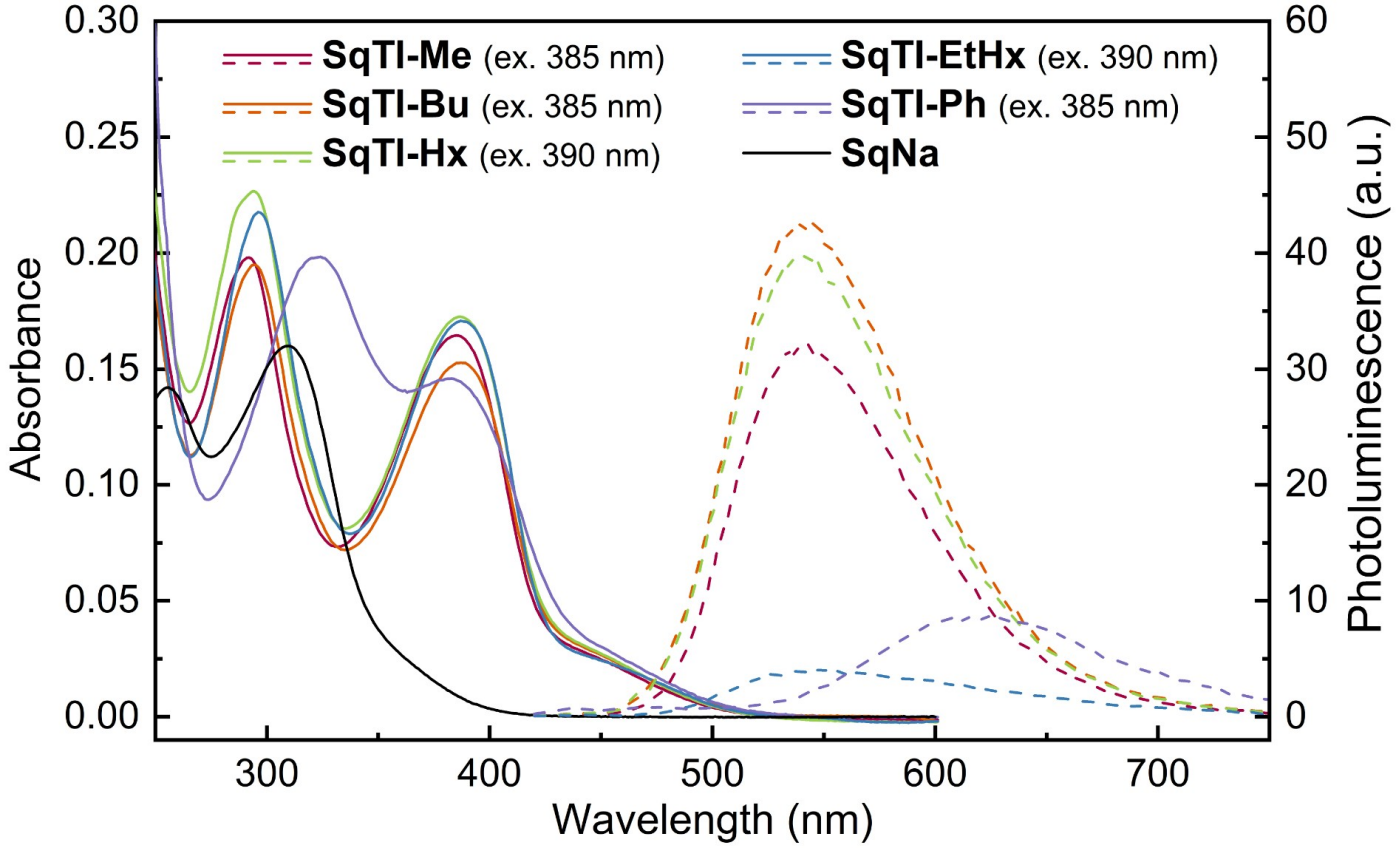
UV/Vis absorption (solid lines) and photoluminescence (PL) spectra (dashed lines) of the macrocycles in CHCl_3_ (**SqTI‐R**, 5 μM) or water (**SqNa**, 5 μM). The excitation wavelengths for recording the photoluminescence (PL) spectra are shown in brackets.

UV/Vis absorption measurements in solution (Figure 6, solid lines) and film (Supporting Information, Figure S43) showed two peaks for all tetraimides **SqTI‐R**. In solution, the first peak was between 382 and 387 nm in all cases, whereas the second peak was at 323 nm for **SqTI‐Ph** and between 292 and 296 nm for all other tetraimides. In the film, these peaks were only marginally redshifted (Supporting Information, Table S5). Compared to the tetraimides, **SqNa** showed drastically blueshifted peaks at 310 and 255 nm. Photoluminescence (PL) measurements (Figure 6, dashed lines) in solution revealed that all target compounds are very weak emitters, with **SqNa** showing only negligible emission. The PL peaks were found to be at 615 nm for **SqTI‐Ph** and between 540 and 542 nm for the other tetraimides.

Finally, thermogravimetric analysis (TGA) revealed the very high thermal stability of the macrocycles, with the decomposition temperature (T_d_, temperature of 5 % weight loss) being above 440 °C for all target compounds and as high as 516 °C for **SqTI‐Ph** (Supporting Information, Figure S47–S49), an important feature for future applications of **SqTI‐R** and other materials based on **SqTA**.

## Conclusion

Our results show that squarephaneic tetraanhydride (**SqTA**), the compound introduced in this work, represents a unique new member of the family of aromatic carboxylic anhydride building blocks. The building block features a macrocyclic paracyclophanetetraene (**PCT**) substructure and four reactive carboxylic anhydride groups for further chemical functionalisation. It can be synthesised in just three steps starting from terephthaloyl chloride (**1**) by making use of a Perkin‐type cyclisation reaction. As shown by the conversion into five different squarephaneic tetraimides (**SqTI‐R**; R=Me, Bu, Hx, EtHx, Ph) and sodium squarephaneate (**SqNa**), **SqTA** can be efficiently functionalised to give materials with highly interesting properties: (i) A void analysis of the crystal structures of **SqTA** and one of the tetraimides, **SqTI‐Bu**, showed that similarly high degrees of porosity as reported for **PCT** can be achieved; neither the anhydride groups in **SqTA** nor the imide groups in **SqTI‐Bu** seemed to affect the porosity. The voids provide space for the insertion of multiple sodium or lithium counterions per macrocycle, a promising feature for application as organic battery electrode materials. (ii) Cyclic voltammetry (CV) measurements of the five squarephaneic tetraimides in solution confirmed that excellent redox properties can be achieved using **SqTA** as a building block. Multiple reversible reduction steps were observed in these measurements, including a two‐electron reduction as the first step. (iii) An investigation of the solid‐state electrochemical activity of **SqTI‐Bu** in lithium‐ion battery half cells showed two sharp redox peaks. The presence and position of the peaks are in good agreement with the findings of the CV measurements in solution, confirming the ability of **SqTI‐Bu** to intercalate lithium ions. However, alternative electrolyte solvents, porous carbons or structural modifications that can prevent the dissolution of the active material will need to be identified in order to improve the cycling performance. (iv) Squarephaneic tetraimides are capable of switching between a locally aromatic neutral state and a globally aromatic doubly reduced state, as observed in computations and confirmed experimentally by low‐temperature NMR measurements of doubly reduced **SqTI‐Hx**. The aromaticity in the doubly reduced state compensates the electrostatic repulsion of the two additional electrons, which explains why the first reduction wave of the tetraimides could be shown to be a two‐electron process. (v) According to the computations, reduction to the tri‐ and tetraanion results in further aromaticity switching. The trianions were found to be locally aromatic, while the tetraanions feature a globally Baird aromatic triplet ground state, which further explains the excellent redox properties observed in the experiments. EPR spectroelectrochemical measurements of **SqTI‐Hx** provide experimental evidence for the triplet ground state of the tetraanion. In contrast to the tetraimides, global ring currents play no role in the neutral and reduced states of **SqNa** that were studied, which were all found to be locally aromatic. (vi) Thermogravimetric analysis (TGA) confirmed that the functionalisation of **SqTA** gives highly stable compounds; the decomposition temperature (T_d_) was above 440 °C in all cases.

Considering the short synthesis and unique properties of **SqTA** and the materials obtained from its further conversion, we expect widespread use of the new building block in the synthesis of organic materials. In particular, we believe that others will find **SqTA** to be an exceptionally useful building block for the synthesis of redox‐active porous organic materials, due to the observed excellent electrochemical properties and the intrinsic porosity resulting from its macrocyclic geometry.

## Conflict of interest

The authors declare no conflict of interest.

1

## Supporting information

As a service to our authors and readers, this journal provides supporting information supplied by the authors. Such materials are peer reviewed and may be re‐organized for online delivery, but are not copy‐edited or typeset. Technical support issues arising from supporting information (other than missing files) should be addressed to the authors.

Supporting Information

## Data Availability

The data that support the findings of this study are available in the supplementary material of this article.
