# Supplementary material for: Squarephaneic Tetraanhydride: A Conjugated Square‐Shaped Cyclophane for the Synthesis of Porous Organic Materials
Source: Angew Chem Int Ed Engl. 2022 Oct 25;61(48):e202212623. doi: 10.1002/anie.202212623 (PMC9827958; doi:10.1002/anie.202212623)
Supplement: Supplementary file 1 — Supporting Information [file ANIE-61-0-s001.pdf]

Angewandte Chemie
Eine Zeitschrift der Gesellschaft Deutscher Chemiker

Supporting Information

Squarephaneic Tetraanhydride: A Conjugated Square-Shaped Cyclophane for the Synthesis of Porous Organic Materials

*S. Eder, B. Ding, D. B. Thornton, D. Sammut, A. J. P. White, F. Plasser, I. E. L. Stephens, M. Heeney, S. Mezzavilla, F. Glöcklhofer**

Table of Contents

1. Synthesis 1
2. ^{1}H and ^{13}C NMR Spectra 9
3. Crystallography 18
4. Electrochemical measurements 21
5. Computational analysis 28
6. EPR spectroelectrochemical measurements 34
7. Chemical reduction and low-temperature NMR measurements 35
8. UV-vis absorption and photoluminescence (PL) spectroscopy 41
9. Thermogravimetric analysis (TGA) 44
10. Infrared (IR) spectroscopy 46
11. References 47

1. Synthesis

Reagents and solvents were purchased from commercial suppliers and used without further purification, including precursors **1** and **4**.
Purification by preparative recycling gel permeation chromatography (GPC) was carried out on a LaboACE LC-5060 (Japan Analytical Industry Co., Tokyo, JAPAN) recycling GPC system equipped with a JAIGEL-2HR column and a TOYDAD800-S detector. DMF (or $CHCl_3$) was used as the eluent at a flow rate of 7 (or 10) mL min^{-1}.
NMR spectra were recorded at 400 MHz for ^{1}H and 101 MHz for ^{13}C on a Bruker AV-400 spectrometer. High-resolution mass spectrometry (HRMS) was carried out on systems from Thermo Scientific (Thermo Scientific Q-Exactive/Dionex Ultimate 3000) for atmospheric pressure chemical ionization (APCI) and Waters (Waters LCT Premier (ES-ToF)/Acquity i-Class) for electrospray ionization (ESI). While the Thermo Scientific system gives the actual mass of the ionized compounds, the Waters system is calibrated to give the mass of the neutral compounds. This was considered when calculating the *m/z* values for comparison with the measurements.

1.1 Terephthaloyl cyanide (2)

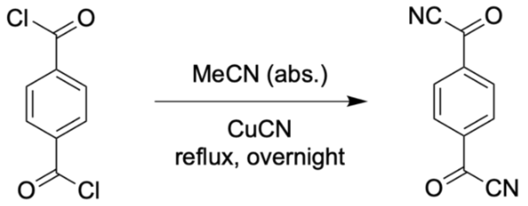

1 Molecular Weight: 203.02

2 Molecular Weight: 184.15

The reaction was carried out by adapting a previously reported procedure.[1]

Terephthaloyl chloride **1** (10.15 g, 50 mmol, 1.0 equiv.) and copper(I) cyanide (9.40 g, 105 mmol, 2.1 equiv.) were suspended in a 250 mL two-neck round-bottom flask in dry acetonitrile (100 mL) under nitrogen atmosphere. The resulting white suspension was heated to reflux and stirred overnight, resulting in a brown solution after 30 min. The reaction was allowed to cool to room temperature and the solvent was evaporated *in vacuo*. The product was separated from insoluble by-products by triturating the brown solid residue with 3 × 200 mL dry boiling toluene. The clear supernatant containing the product was decanted off each time through a sintered glass funnel. Evaporation of the solvent afforded the crude product as an off-white powder. Purification by sublimation (0.2 mbar, 140 °C) gave **2** as a white solid in yields of 81% (7.45 g, 40 mmol). ^{1}H NMR (400 MHz, $CDCl_3$): δ = 8.39 (s, 4H) ppm. ^{13}C NMR (101 MHz, $CDCl_3$): δ = 166.92, 138.45, 131.19, 112.24 ppm. NMR spectra in accordance with the literature.[2] *Note: Purification by sublimation sometimes yielded an impure product in our experiments. The impurity can be removed by recrystallisation from toluene if it is present. It is further advisable to use only dry solvents in the work-up. It is also recommended to use dry DMSO-d_6 to assess the purity of the product by NMR spectroscopy.*

1.2 α^1,α^4-Dioxo-1,4-benzenediacetic acid (3)

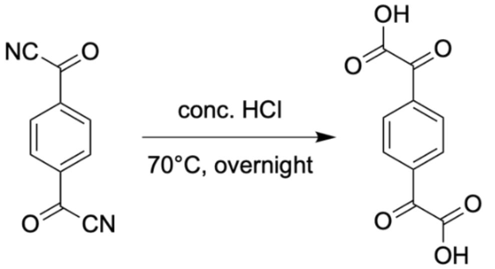

2 Molecular Weight: 184.15

3 Molecular Weight: 222.15

The reaction was inspired by a previously reported procedure for the synthesis of the corresponding mono-substituted compound.[3]

Terephthaloyl cyanide **2** (3.76 g, 20.4 mmol, 1.0 equiv.) was added in one potion to 80 mL of rigorously stirred conc. HCl (preheated to 70 °C) and stirred overnight at 70 °C. The white suspension was allowed to cool to room temperature, diluted with 100 mL water, filtered through a sintered glass funnel, and washed with 50 mL water. The filtrate was further diluted with water and filtered again if additional solid precipitated. The clear solution was extracted

with 6 × 200 mL Et_2O, the combined organic layers dried over $MgSO_4$, filtered and the solvent evaporated *in vacuo*. The remaining off-white powder was purified by sublimation (0.20 mbar, 145 °C), hereby removing side products and leaving the product behind. The remaining solid was triturated two times with refluxing $CHCl_3$ in between sublimations to allow for sublimation of further side product that was incorporated in the product powder. **3** was obtained as off-white powder in sufficient purity of 98% (based on NMR measurements) in yields of 62% (2.82 g, 12.7 mmol). ^{1}H NMR (400 MHz, DMSO-d$_6$): δ = 8.14 (s, 4H) ppm. ^{13}C NMR (101 MHz, DMSO-d$_6$): δ = 187.81, 165.04, 136.33, 130.13 ppm. NMR spectra in accordance with the literature.[4] *Note: It is recommended to use dry DMSO-d$_6$ to assess the purity of the product by NMR spectroscopy.*

1.3 Synthesis of squarephaneic tetraanhydride (SqTA)

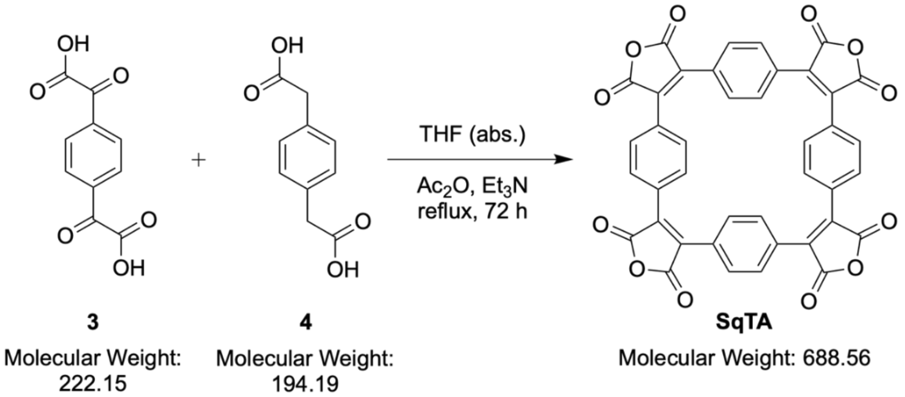

The synthesis of **SqTA** was carried out under the conditions used for the synthesis of ester-substituted derivatives of **SqTA**,[4] but the reaction was stopped at the anhydride stage, omitting the addition of reagents for the further conversion into ester groups. The previously reported synthesis of the ester-substituted derivatives was adapted from Durola et al.[5]

A 1 L two-neck round-bottom flask was charged with 380 mL dry degassed THF (no stabilizer), acetic anhydride (2.6 mL, 27 mmol, 15 equiv.) and triethylamine (2.5 mL, 18 mmol, 10 equiv.) and heated to reflux under nitrogen atmosphere. Meanwhile, precursors **3** (400 mg, 1.8 mmol, 1.0 equiv.) and **4** (350 mg, 1.8 mmol, 1.0 equiv.) were dissolved in 80 mL dry THF in an oven-dry 100 mL flask and degassed. The resulting solution was added dropwise to the reaction over 20 h (4 mL h^{-1}) using a syringe pump and the reaction mixture stirred at reflux for 72 h in total. The dark green solution was then allowed to cool to room temperature and all volatiles were evaporated *in vacuo*. The resulting brown oil was treated with 80 mL 2 M HCl in an ultrasonic bath for 30 min, resulting in the formation of an orange suspension that was filtered through a sintered glass funnel and washed with 50 mL H_2O. Drying in high vacuum yielded 598 mg crude product, which was dissolved in 5 mL DMF and filtered through a 0.2 µm syringe filter before loading the GPC column with the resulting solution (DMF was used as eluent for the GPC, 7 mL min^{-1}). After purification by GPC, evaporation of DMF (and co-evaporation with toluene to remove residues of DMF) yielded 200 mg **SqTA** (0.29 mmol, 32%) as an orange powder. ^{1}H NMR (400 MHz, DMSO-d$_6$): δ = 7.69 (s, 16H) ppm. ^{13}C NMR (101 MHz, DMSO-d$_6$): δ = 164.75, 137.21, 130.03, 129.46 ppm. HRMS (ESI): *m/z* calcd for $C_{40}H_{15}O_{12}^-$: 687.0564 [*M*–H]; found: 687.0597. UV-Vis: λ_{max} = 357 nm (MeCN).

As a scalable alternative, a fed-batch approach for the synthesis of **SqTA** was tested in which we added two additional portions of precursors **3** and **4** to the same solution after the initial addition. In total, three portions of precursors were added over 3 × 20 h (5.4 mmol of each precursor) before stirring for 72 h at reflux (no additional acetic anhydride or triethylamine were added). The work-up was performed as described above, but the crude product was separated into 500 mg portions in order to prevent an overloading of the GPC column. The same percent yield of 32% (0.87 mmol, 602 mg) was achieved.

1.4 Model Compound *N*-butyl-2,3-diphenylmaleimide (MC)

The conversion of **SqTA** into squarephaneic tetraimides (**SqTI-R**) was tested using 2,3-diphenylmaleic anhydride as model compound.

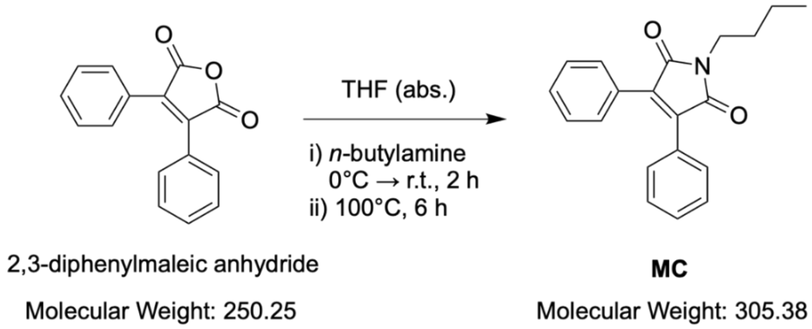

2,3-Diphenylmaleic anhydride (50 mg, 0.2 mmol, 1.0 equiv.) was dissolved in 2 mL THF (dry, degassed) in a microwave vial under nitrogen atmosphere. The slightly yellow solution was treated with 0.2 mL 2 M *n*-butylamine solution in THF (0.4 mmol, 2.0 equiv.) and stirred at room temperature for 2 h until thin-layer chromatography (TLC) indicated full conversion of the starting material. The reaction was then heated to 100 °C for 6 h until TLC showed complete conversion of the intermediate. After cooling to room temperature, all volatiles were evaporated *in vacuo* and the resulting yellow oil dried under high vacuum (0.20 mbar). **MC** was obtained as a pale-yellow solid in quantitative yields (61 mg, 0.2 mmol, 100%). ^{1}H NMR (400 MHz, $CDCl_3$): δ = 7.52–7.30 (m, 10H), 3.66 (t, *J* = 7.2 Hz, 2H), 1.71–1.62 (m, 2H), 1.43–1.34 (m, 2H), 0.96 (t, *J* = 7.3 Hz, 3H) ppm.

1.5 Synthesis of squarephaneic tetraimides (SqTI-R)

1.5.1 General Procedure

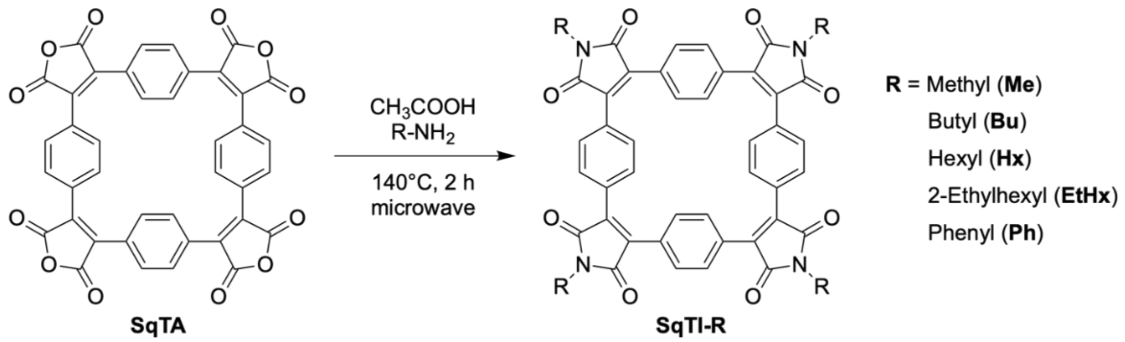

In a microwave vial, **SqTA** (1.0 equiv.) was suspended in 2 mL glacial acetic acid and degassed with nitrogen. To the resulting orange suspension, the respective alkylamine or phenylamine was added via syringe under vigorous stirring. The reaction was then heated to 140 °C in a microwave reactor for 2 h. After cooling to room temperature, the resulting orange suspension was diluted with 2 mL H_2O, filtered through a sintered glass funnel, and washed with water to afford the respective squarephaneic tetraimide **SqTI-R**. After drying, the product was usually obtained in high purity, but in some instances further purification or an alternative work-up was required.

Alternatively, crude **SqTA** (not purified by GPC) can be converted directly to the respective **SqTI-R** and purified by GPC (using $CHCl_3$ as eluent, 10 mL min^{-1}) or silica gel chromatography, as demonstrated for some tetraimides.

1.5.2 Macrocycle SqTI-Me

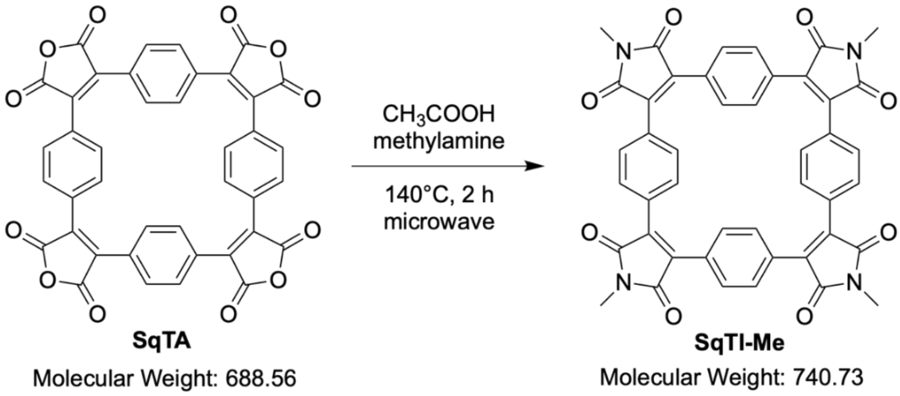

*Synthesis of **SqTI-Me** following the general procedure:* **SqTA** (60 mg, 0.087 mmol, 1.0 equiv.), glacial acetic acid (2 mL) and methylamine (1.74 mL of 2.0 M solution in THF, 3.5 mmol, 40 equiv.) were used. After the general work-up, the crude product was dissolved in CH_2Cl_2 and filtered to remove insoluble side products. Evaporation of the solvent *in vacuo* afforded **SqTI-Me** as an orange solid in a yield of 80% (52 mg, 0.070 mmol). ^{1}H NMR (400 MHz, $CDCl_3$): δ = 7.72 (s, 16H), 3.16 (s, 12H) ppm. ^{13}C NMR (101 MHz, $CDCl_3$): δ = 170.22, 135.23, 130.45, 130.38, 24.65 ppm. HRMS (APCI): *m/z* calcd for $C_{44}H_{29}N_4O_8^+$: 741.1980 [*M*+H]$^+$; found: 741.1971. UV-Vis: λ_{max} = 292 nm ($CHCl_3$).

1.5.3 Macrocycle SqTI-Bu

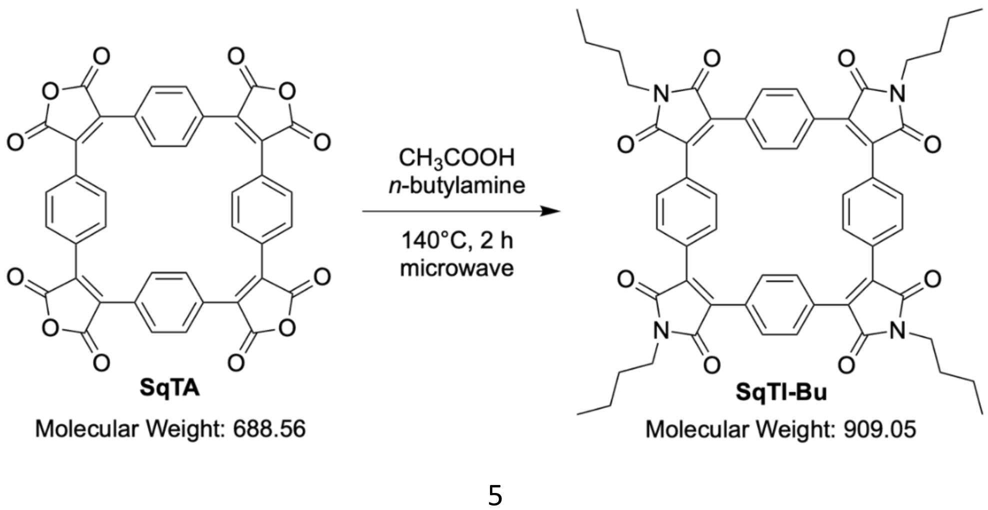

*Synthesis of **SqTI-Bu** following the general procedure:* **SqTA** (60 mg, 0.087 mmol, 1.0 equiv.), glacial acetic acid (2 mL) and *n*-butylamine (0.07 mL, 0.7 mmol, 8.0 equiv.) were used. **SqTI-Bu** was obtained as an orange solid in a yield of 87% (69 mg, 0.076 mmol). ^{1}H NMR (400 MHz, $CDCl_3$): δ = 7.73 (s, 16H), 3.64 (t, J = 7.3 Hz, 8H), 1.69–1.60 (m, 8H), 1.43–1.31 (m, 8H), 0.96 (t, J = 7.3 Hz, 12H) ppm. ^{13}C NMR (101 MHz, $CDCl_3$): δ = 170.20, 134.96, 130.47, 130.39, 38.53, 30.75, 20.21, 13.79 ppm. HRMS (ESI): *m/z* calcd for $C_{58}H_{56}N_5O_8^+$: 950.4129 [*M*+MeCN+H]; found: 950.4130. UV-Vis: λ_{max} = 294 nm ($CHCl_3$).

1.5.4 Macrocycle SqTI-Hx

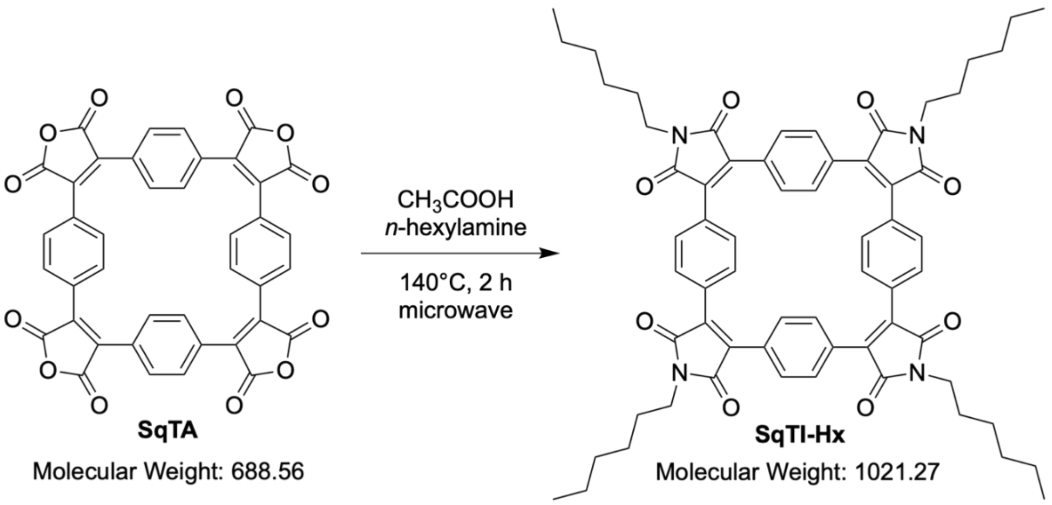

*Synthesis of **SqTI-Hx** following the general procedure:* **SqTA** (60 mg, 0.087 mmol, 1.0 equiv.), glacial acetic acid (2 mL) and *n*-hexylamine (0.09 mL, 0.7 mmol, 8.0 equiv.) were used. **SqTI-Hx** was obtained as an orange solid in a yield of 94% (84 mg, 0.082 mmol). ^{1}H NMR (400 MHz, $CDCl_3$): δ = 7.73 (s, 16H), 3.63 (t, J = 7.3 Hz, 8H), 1.70–1.60 (m, 8H), 1.37–1.27 (m, 24H), 0.89 (t, J = 7.1 Hz, 12H) ppm. ^{13}C NMR (101 MHz, $CDCl_3$): δ = 170.18, 134.98, 130.47, 130.42, 38.83, 31.49, 28.65, 26.65, 22.65, 14.17 ppm. HRMS (ESI): *m/z* calcd for $C_{66}H_{72}N_5O_8^+$: 1062.5381 [*M*+MeCN+H]; found: 1062.5364. UV-Vis: λ_{max} = 294 nm ($CHCl_3$).

Alternatively, the reaction can be carried out employing crude (not purified by GPC) **SqTA** (60 mg, 0.087 mmol, 1.0 equiv.) and *n*-hexylamine (0.09 mL, 0.7 mmol, 8.0 equiv.), affording a mixture of product and oligomeric / polymeric side products. The product can be purified by GPC (using $CHCl_3$ as the eluent, 10 mL min^{-1}), affording **SqTI-Hx** in 24% (21 mg, 0.021 mmol) overall yield (with respect to the cyclisation precursors).

1.5.5 Macrocycle SqTI-EtHx

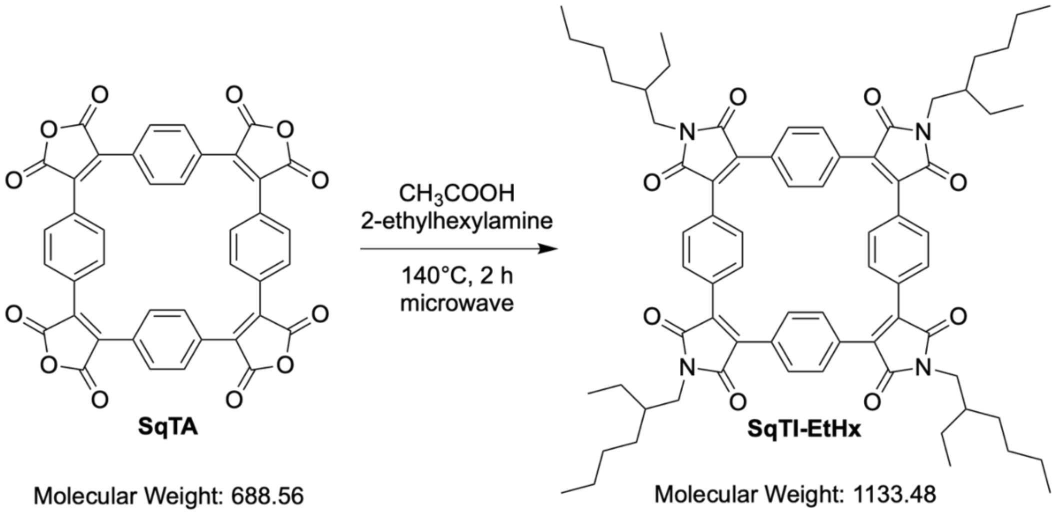

*Synthesis of **SqTI-EtHx** following the general procedure:* **SqTA** (60 mg, 0.087 mmol, 1.0 equiv.), glacial acetic acid (2 mL) and 2-ethylhexylamine (0.12 mL, 0.7 mmol, 8.0 equiv.) were used. Instead of the general work-up, the reaction was diluted with 20 mL CH_2Cl_2 and extracted with 20 mL H_2O. The organic layer was dried over $MgSO_4$, filtered, and the solvent evaporated. The crude red oil was purified by silica gel chromatography (40 g of silica gel) using petroleum ether:ethyl acetate (6:1) as the eluent. After evaporation of the solvent, **SqTI-EtHx** was obtained as an orange solid in a yield of 63% (62 mg, 0.055 mmol). ^{1}H NMR (400 MHz, $CDCl_3$): δ = 7.73 (s, 16H), 3.53 (d, J = 7.1 Hz, 8H), 1.82–1.74 (m, 4H), 1.39–1.24 (m, 32H), 0.95–0.88 (m, 24H) ppm. ^{13}C NMR (101 MHz, $CDCl_3$): δ = 170.44, 134.92, 130.48, 130.45, 42.57, 38.50, 30.67, 28.69, 24.03, 23.14, 14.23, 10.57 ppm. UV-Vis: λ_{max} = 296 nm ($CHCl_3$).

Alternatively, the reaction can be carried out employing crude (not purified by GPC) **SqTA** (60 mg, 0.087 mmol, 1.0 equiv.) and 2-ethylhexylamine (0.12 mL, 0.7 mmol, 8.0 equiv.), affording a mixture of product and oligomeric / polymeric side products. The product can be purified by GPC (using $CHCl_3$ as the eluent, 10 mL min^{-1}), affording **SqTI-EtHx** in 13% (13 mg, 0.011 mmol) overall yield (with respect to the cyclisation precursors). As an alternative to the purification by GPC, purification by silica gel chromatography (40 g of silica gel) using petroleum ether:ethyl acetate (6:1) as the eluent and subsequent trituration in hexane gave **SqTI-EtHx** in 13 % (13 mg, 0.011 mmol) overall yield (with respect to the cyclisation precursors).

1.5.6 Macrocycle SqTI-Ph

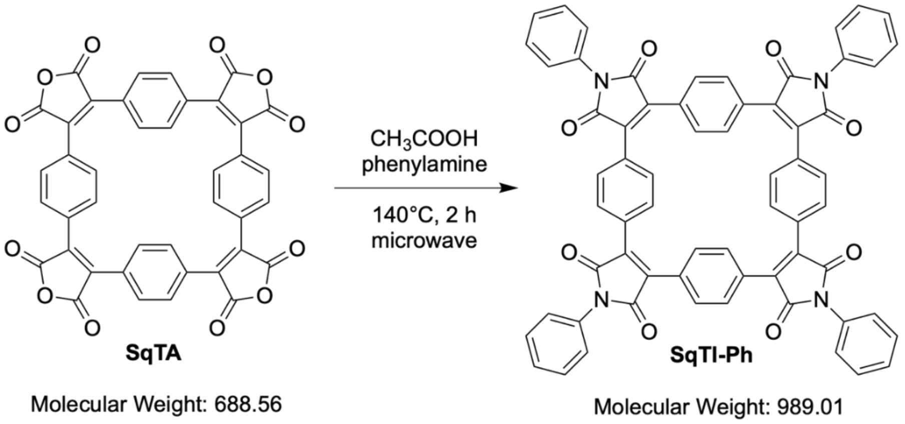

*Synthesis of **SqTI-Ph** following the general procedure:* **SqTA** (60 mg, 0.087 mmol, 1.0 equiv.), glacial acetic acid (2 mL) and aniline (0.06 mL, 0.7 mmol, 8.0 equiv.) were used. After the general work-up, the crude product was dissolved in CH_2Cl_2 and filtered over a thick pad of silica gel using 250 mL CH_2Cl_2 as the eluent. Evaporation of the solvent *in vacuo* afforded **SqTI-Ph** as an orange solid in a yield of 86% (74 mg, 0.075 mmol). ^{1}H NMR (400 MHz, $CDCl_3$): δ = 7.84 (s, 16H), 7.53–7.38 (m, 20H) ppm. ^{13}C NMR (101 MHz, $CDCl_3$): δ = 168.93, 135.19, 131.63, 130.74, 130.45, 129.33, 128.23, 126.32 ppm. HRMS (ESI): *m/z* calcd for $C_{66}H_{40}N_5O_8^+$: 1030.2877 [*M*+MeCN+H]; found: 1030.2882. UV-Vis: λ_{max} = 323 nm ($CHCl_3$).

1.6 Synthesis of sodium squarephaneate (SqNa)

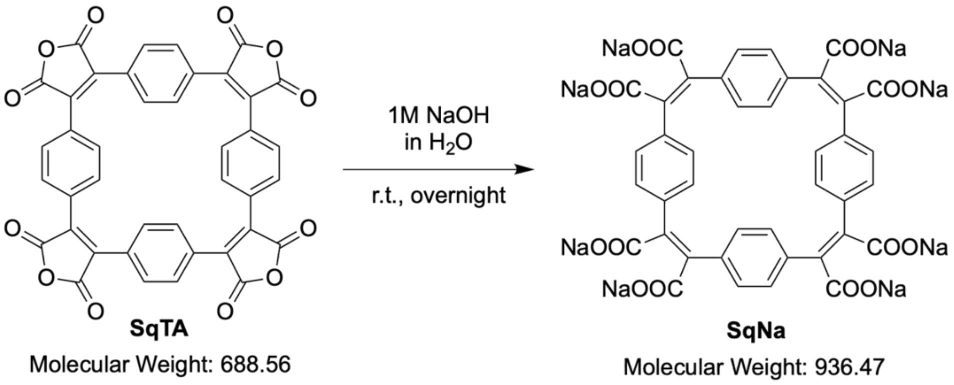

SqTA (200 mg, 0.290 mmol, 1.0 equiv.) was suspended in aqueous 1 M NaOH (2.3 mL, 2.32 mmol, 8.0 equiv.) and the reaction stirred overnight. The product was precipitated by slowly dropping the reaction solution into 10 mL vigorously stirred acetone. The suspension was centrifuged, and the clear supernatant taken off. After an additional washing step (suspending in acetone, centrifuging, taking off supernatant), **SqNa** was obtained as off-white solid in 91% yield (248 mg, 0.265 mmol). ^{1}H NMR (400 MHz, D_2O): δ = 6.87 (s, 16H) ppm. ^{13}C NMR (101 MHz, D_2O): δ = 177.62, 138.33, 136.57, 129.31 ppm. HRMS (ESI): *m/z* calcd for $C_{40}H_{23}O_{16}^-$: 759.0986 [*M*–H]; found: 759.0990 (single negative charge after all sodium ions were exchanged by protons). UV-Vis: λ_{max} = 310 nm (H_2O). Elemental analysis calcd for $C_{40}H_{16}Na_8O_{16}\cdot 2.5H_2O$: C 48.95, H 2.16, N 0.00, found: C 48.84, H 1.98, N 0. The results indicate that the compound is hygroscopic, as the sample was dried in vacuum at 120 °C for 2 h before being sent for elemental analysis.

2. ^{1}H and ^{13}C NMR Spectra

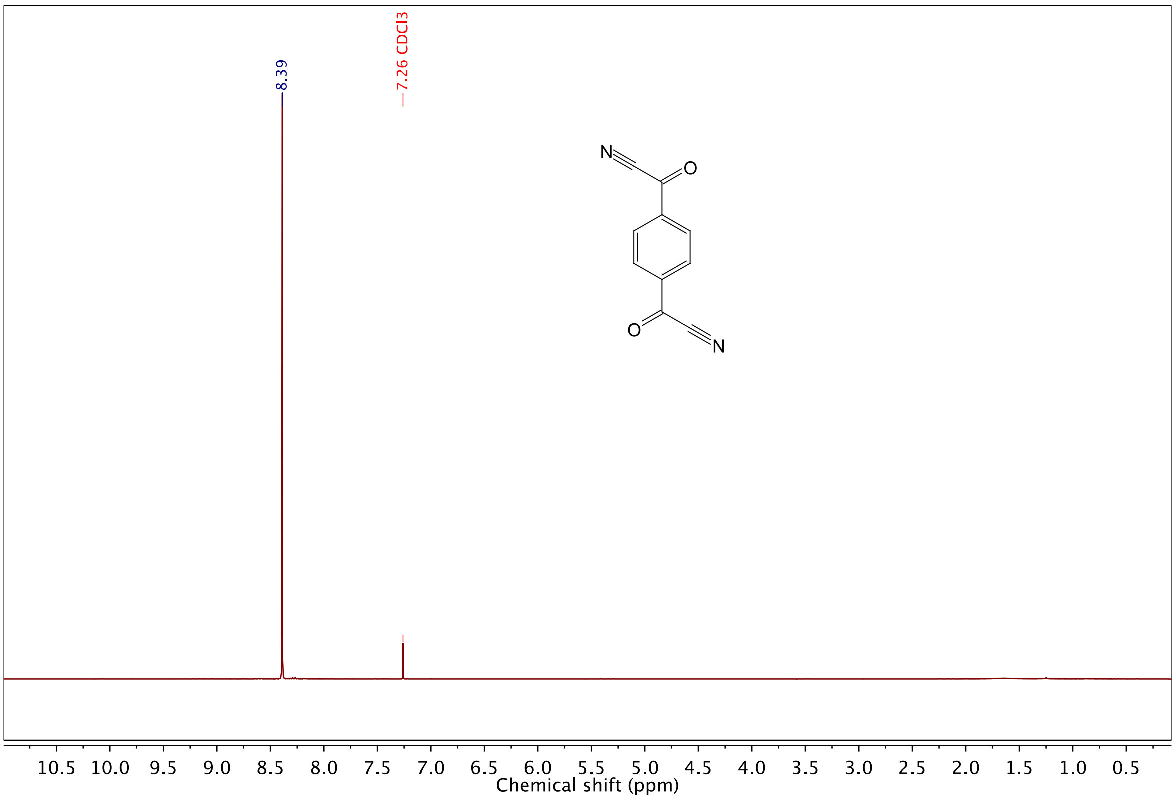

Figure S1. ^{1}H NMR (400 MHz, $CDCl_3$) of compound **2**.

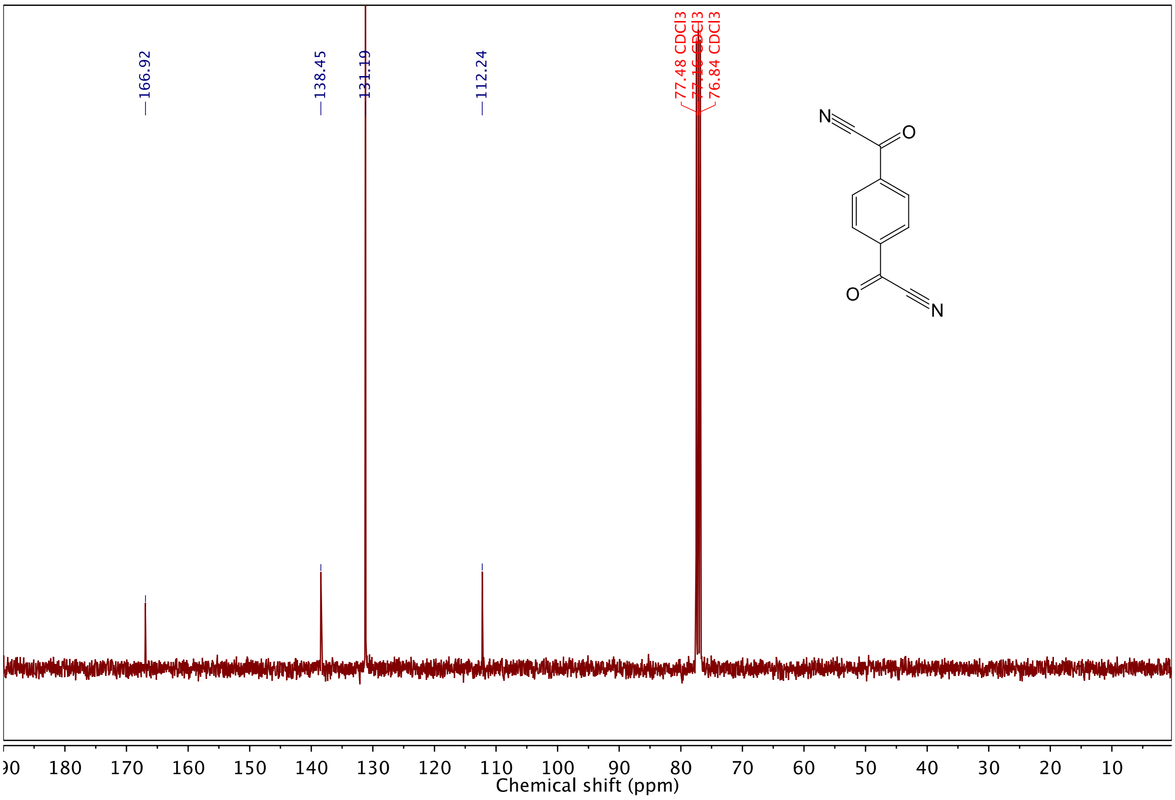

Figure S2. ^{13}C NMR (101 MHz, $CDCl_3$) of compound **2**.

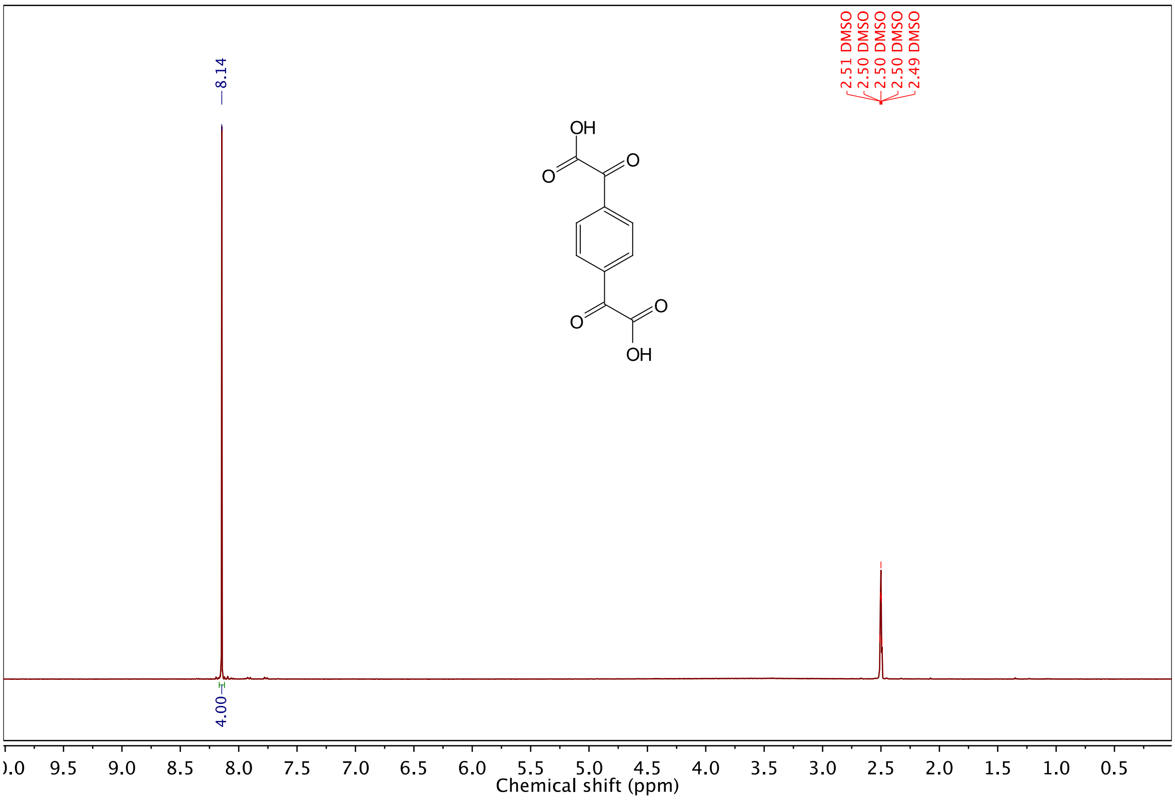

Figure S3. ^{1}H NMR (400 MHz, DMSO-d_6) of compound **3**.

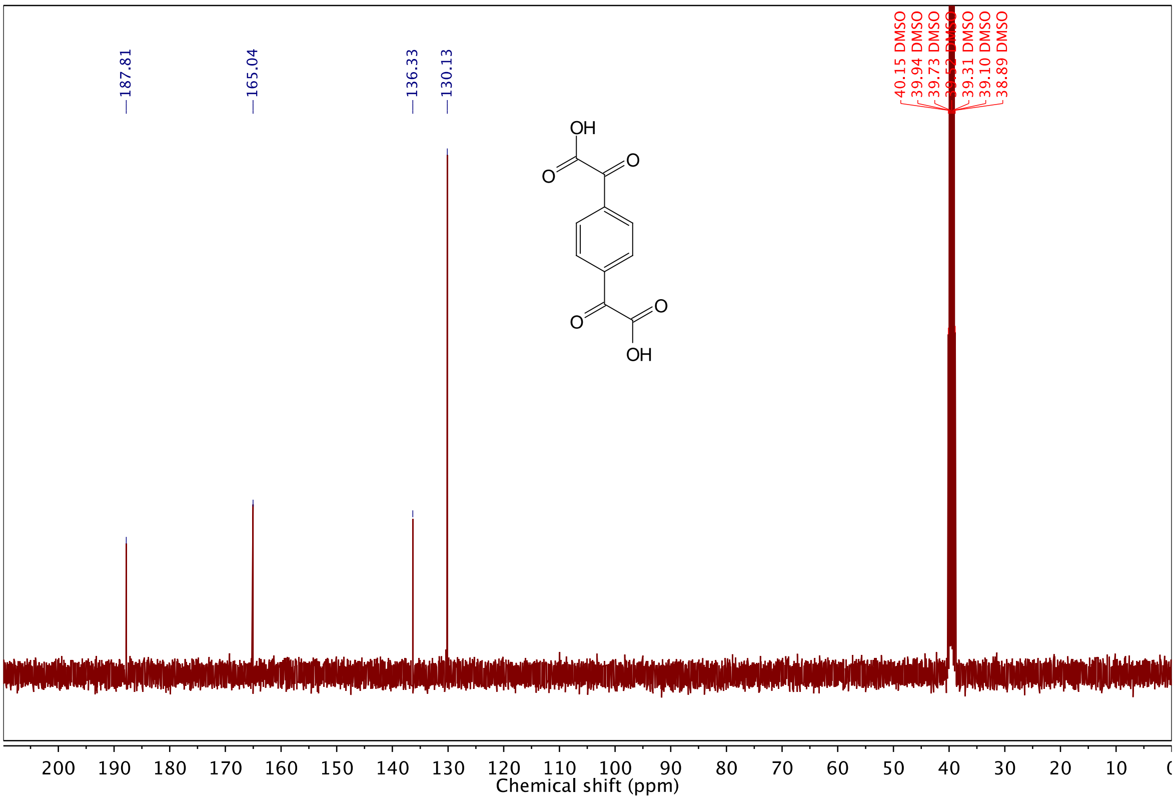

Figure S4. ^{13}C NMR (101 MHz, DMSO-d_6) of compound **3**.

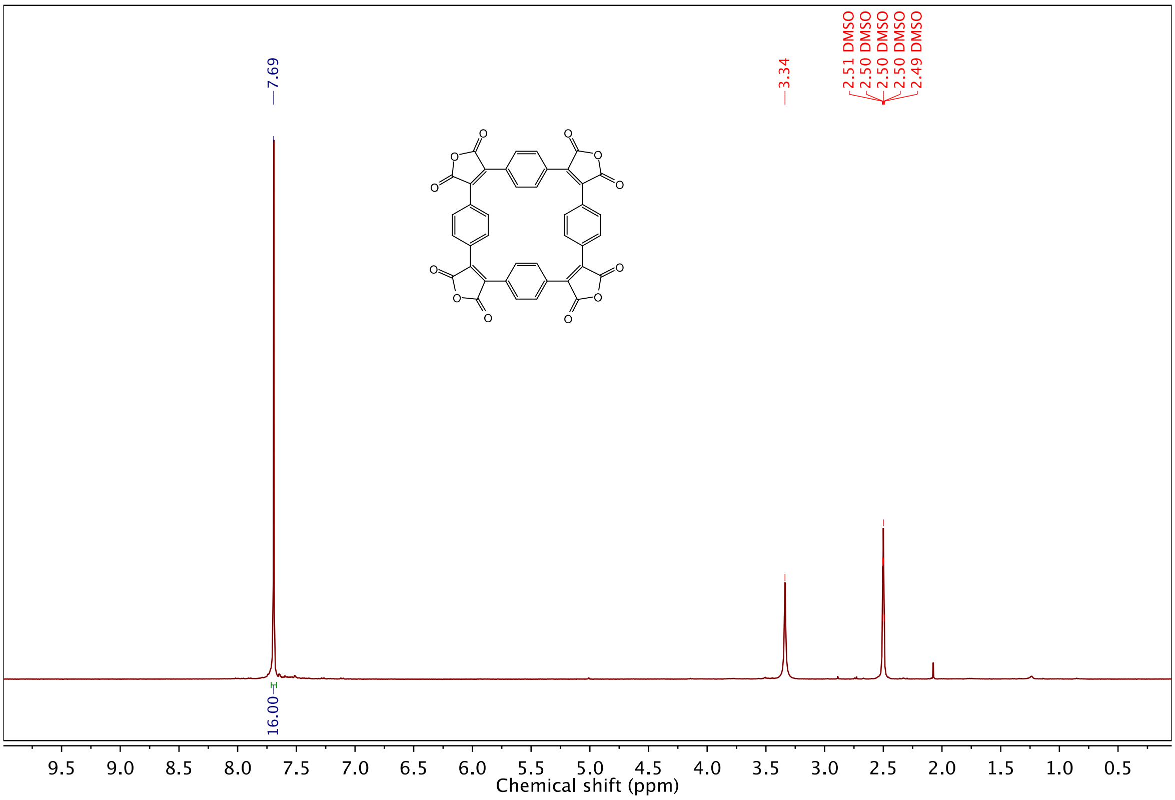

Figure S5. ^{1}H NMR (400 MHz, DMSO-d_6) of compound **SqTA**.

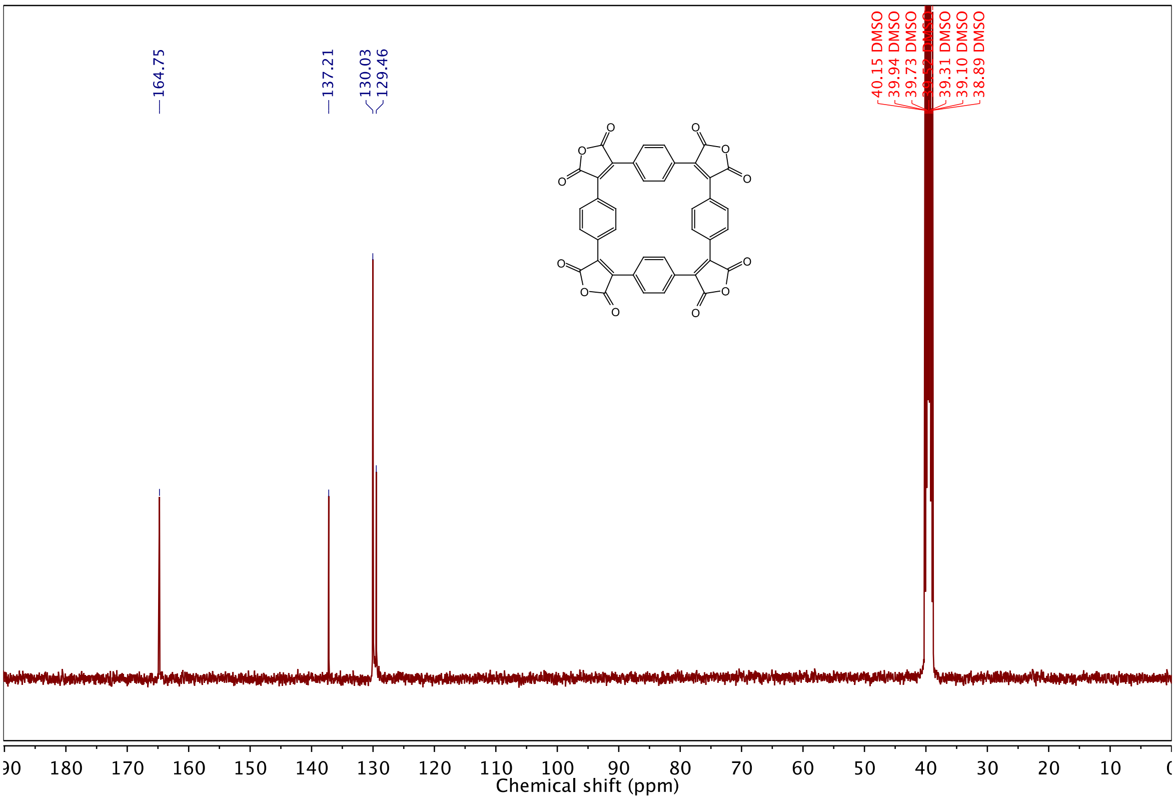

Figure S6. ^{13}C NMR (101 MHz, DMSO-d_6) of compound **SqTA**.

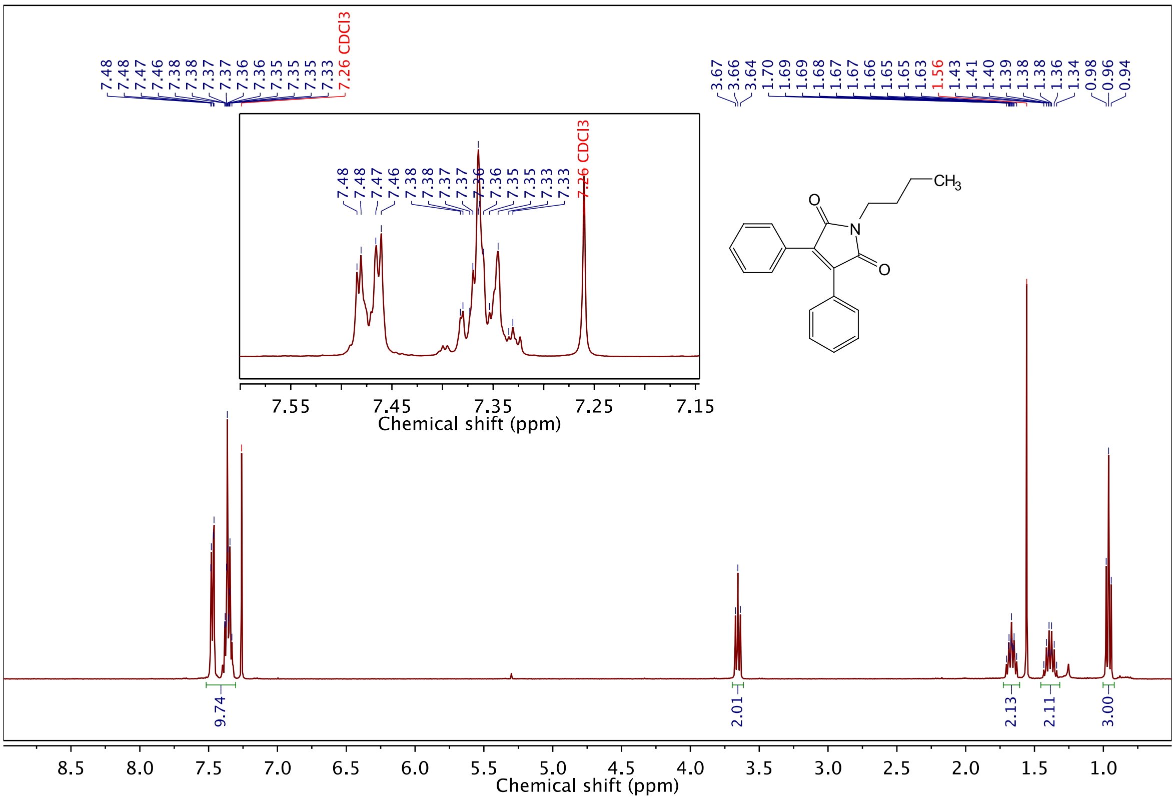

Figure S7. ^{1}H NMR (400 MHz, $CDCl_3$) of compound **MC**.

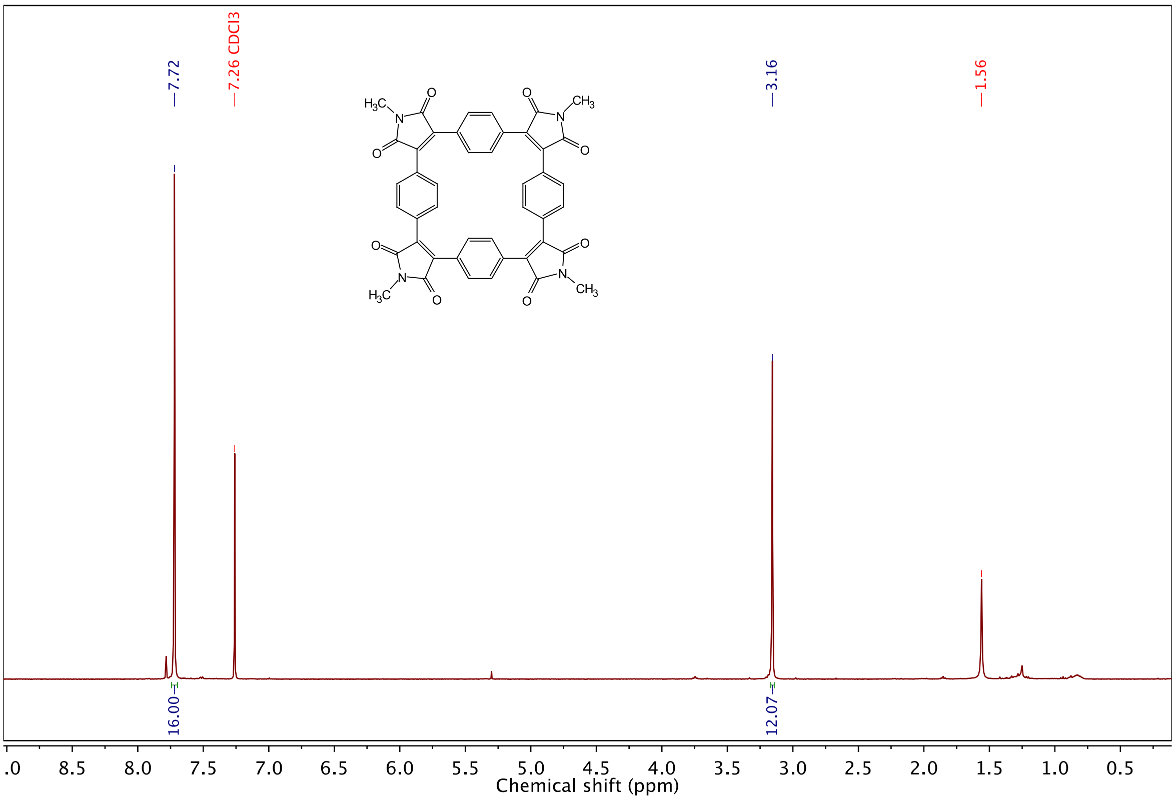

Figure S8. ^{1}H NMR (400 MHz, $CDCl_3$) of compound **SqTI-Me**.

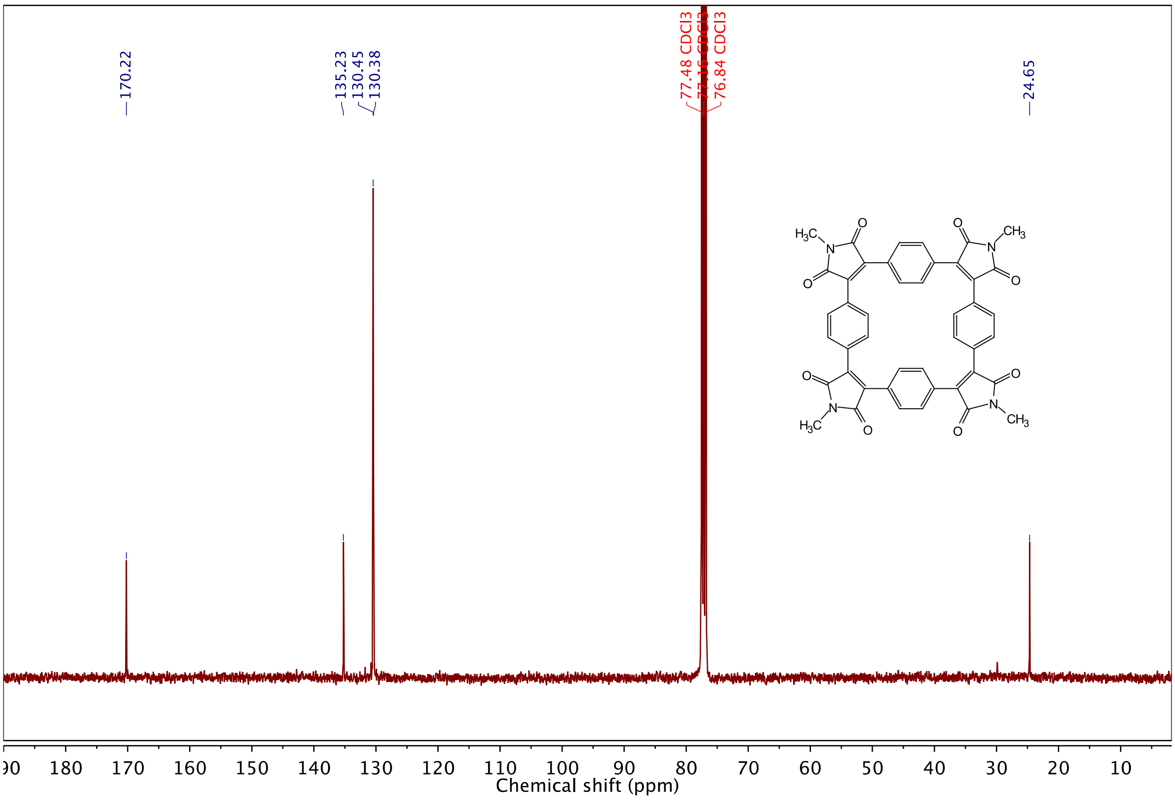

Figure S9. ^{13}C NMR (101 MHz, $CDCl_3$) of compound **SqTI-Me**.

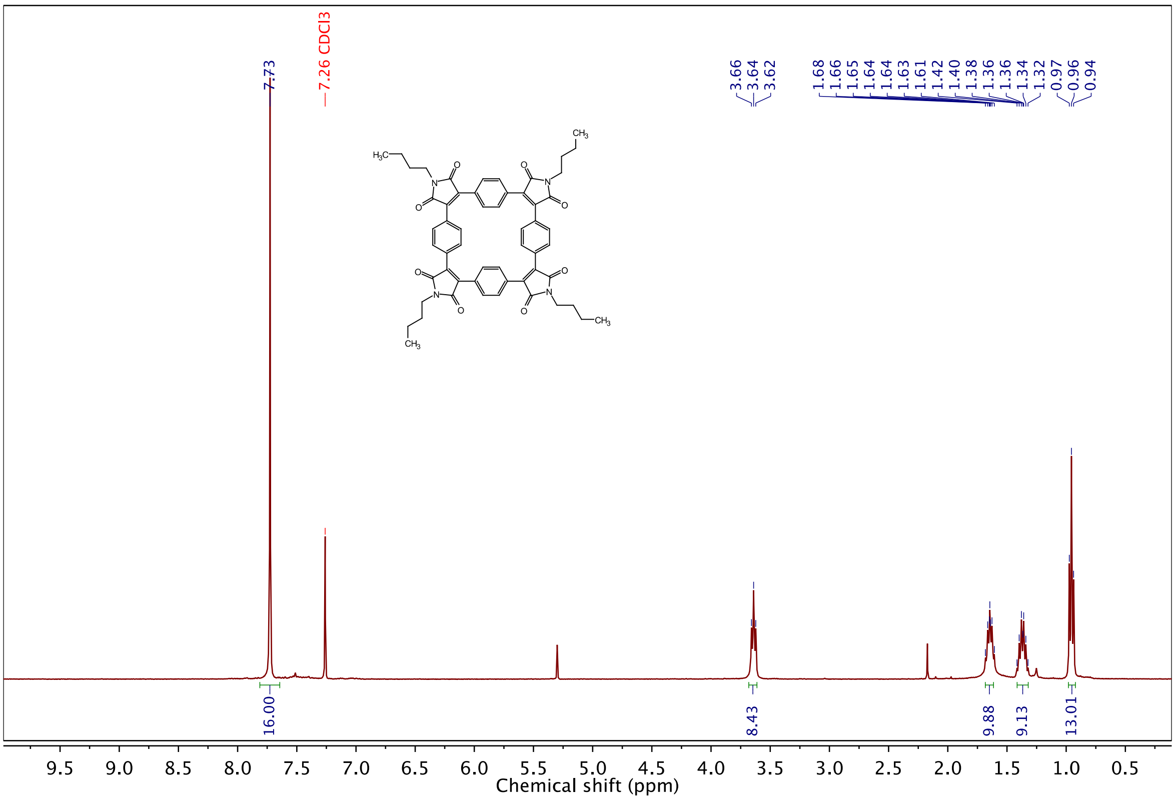

Figure S10. ^{1}H NMR (400 MHz, $CDCl_3$) of compound **SqTI-Bu**.

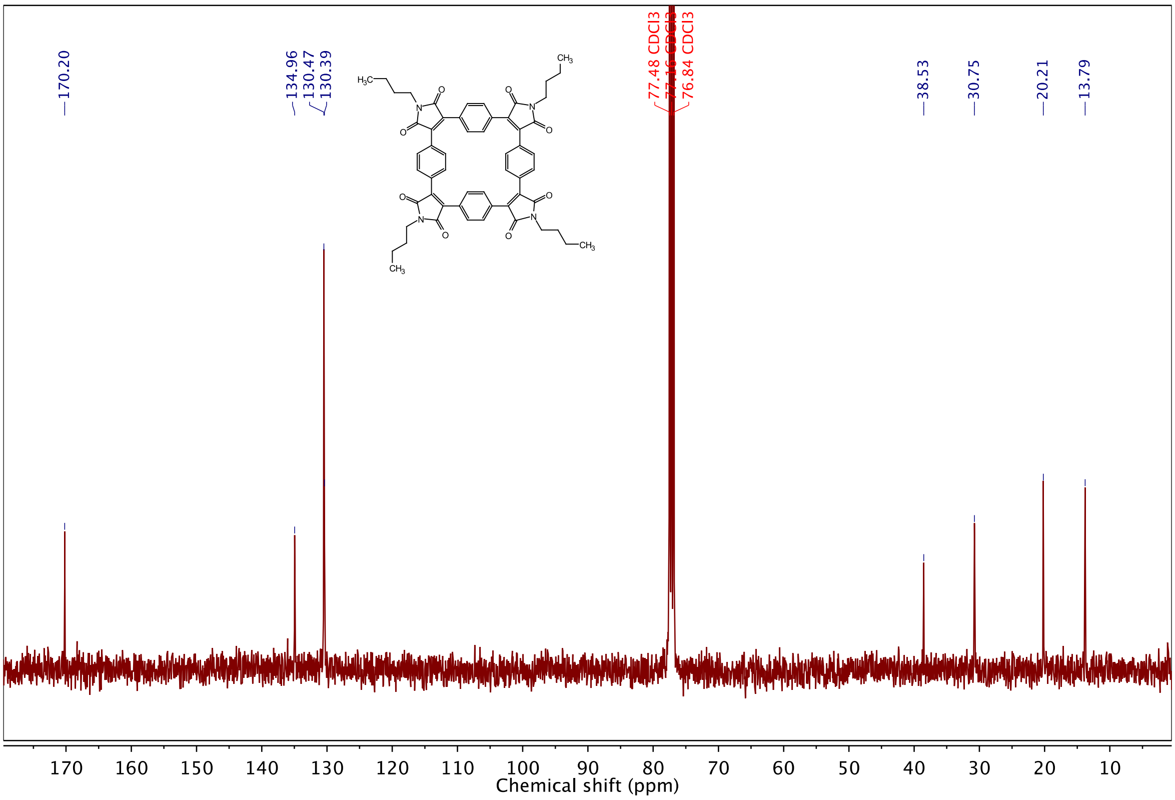

Figure S11. ^{13}C NMR (101 MHz, $CDCl_3$) of compound **SqTI-Bu**.

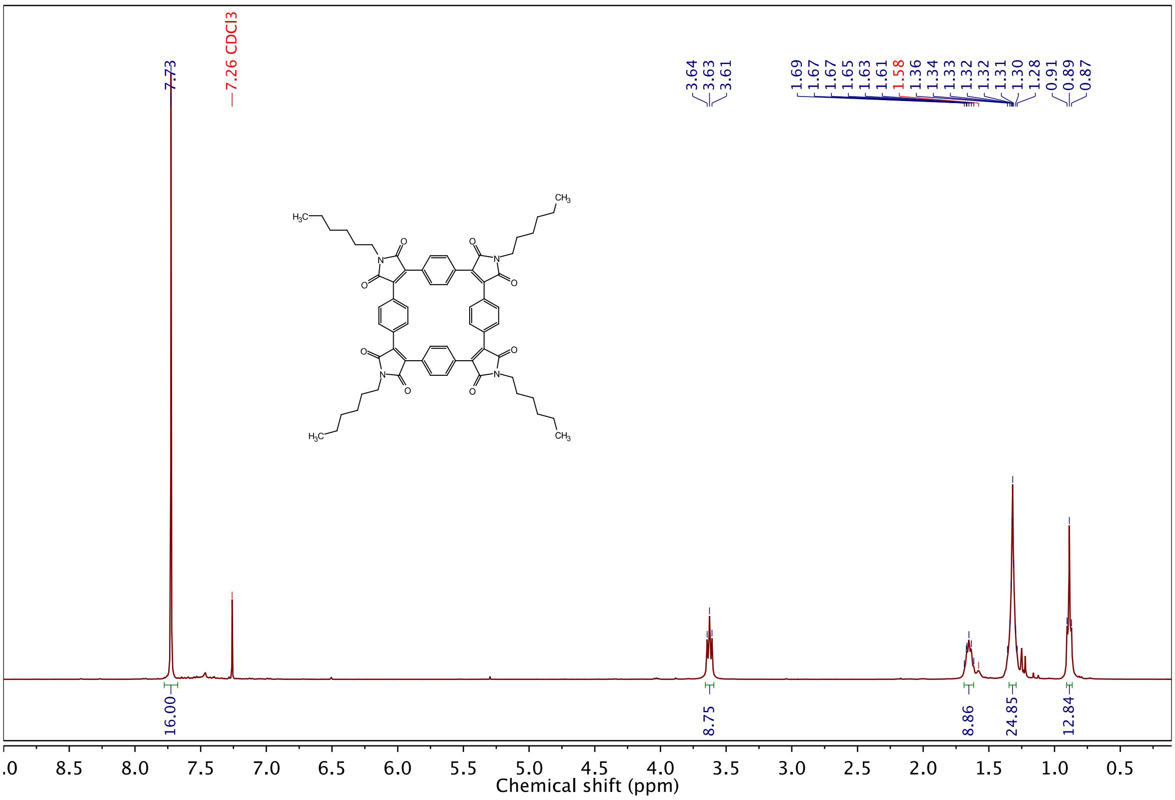

Figure S12. ^{1}H NMR (400 MHz, $CDCl_3$) of compound **SqTI-Hx.**

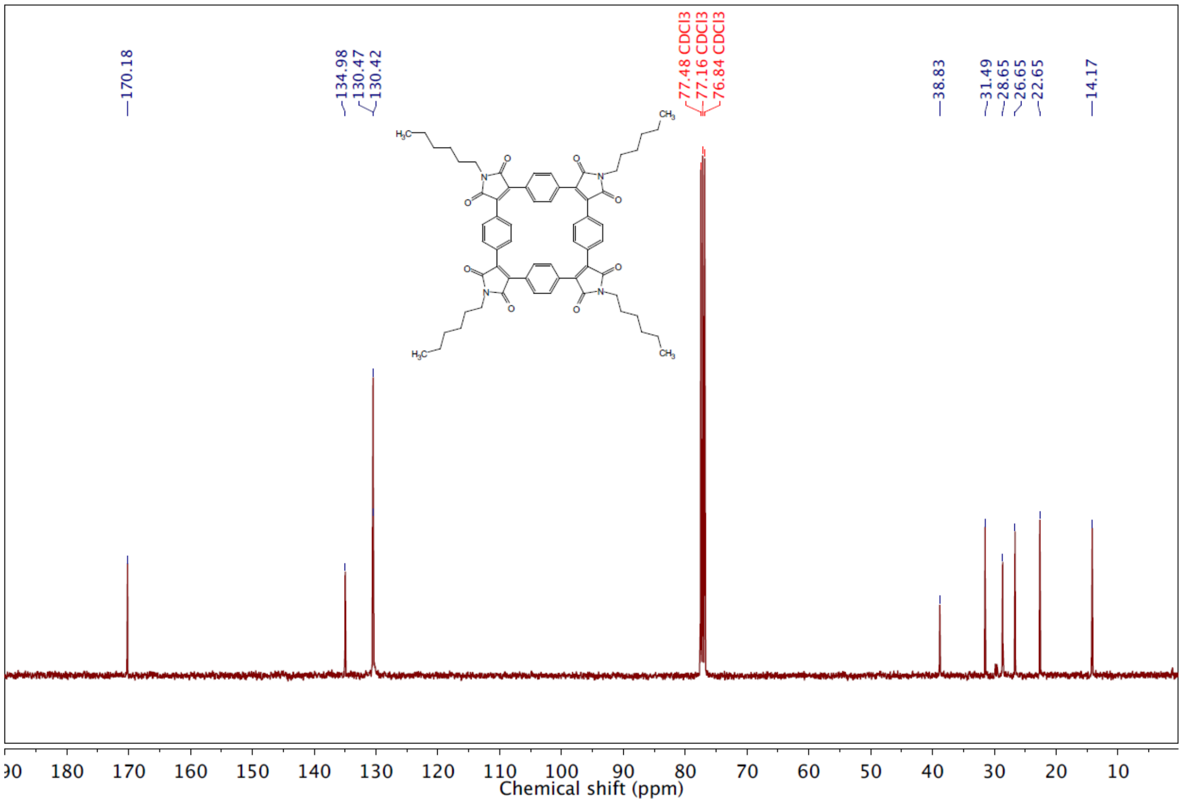

Figure S13. ^{13}C NMR (101 MHz, $CDCl_3$) of compound **SqTI-Hx**.

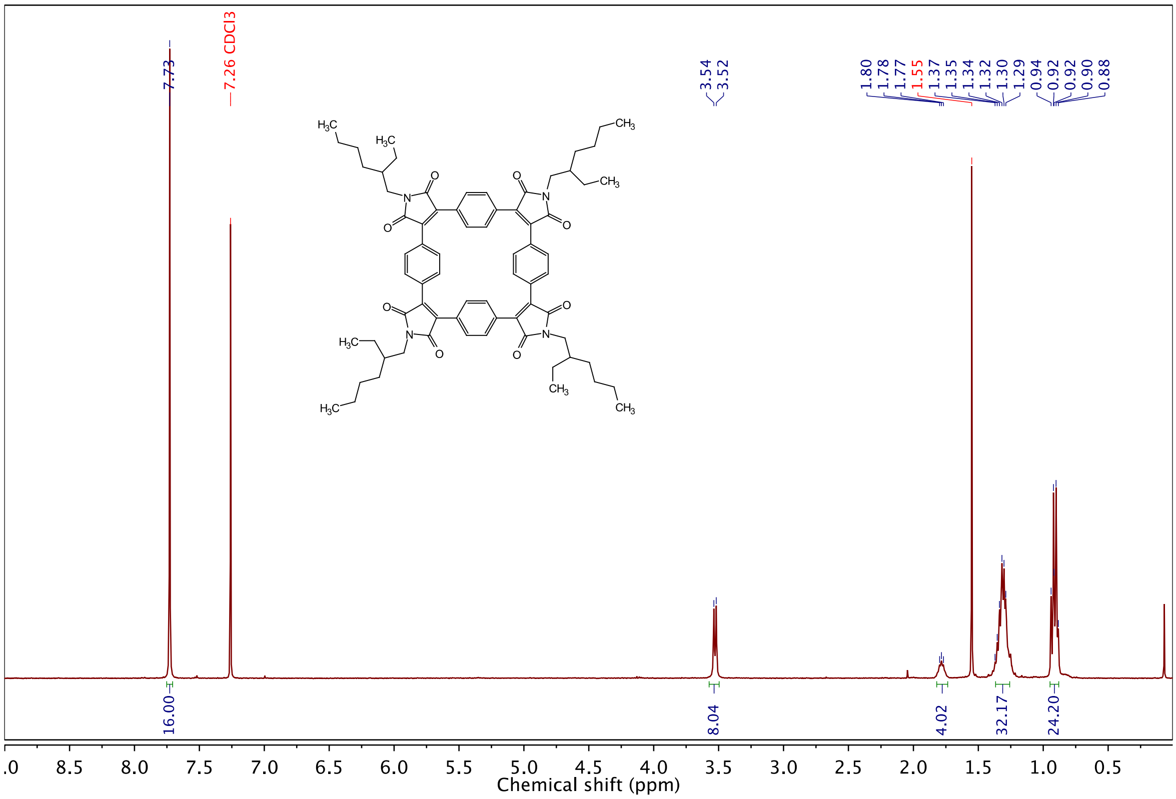

Figure S14. ^{1}H NMR (400 MHz, $CDCl_3$) of compound **SqTI-EtHx**.

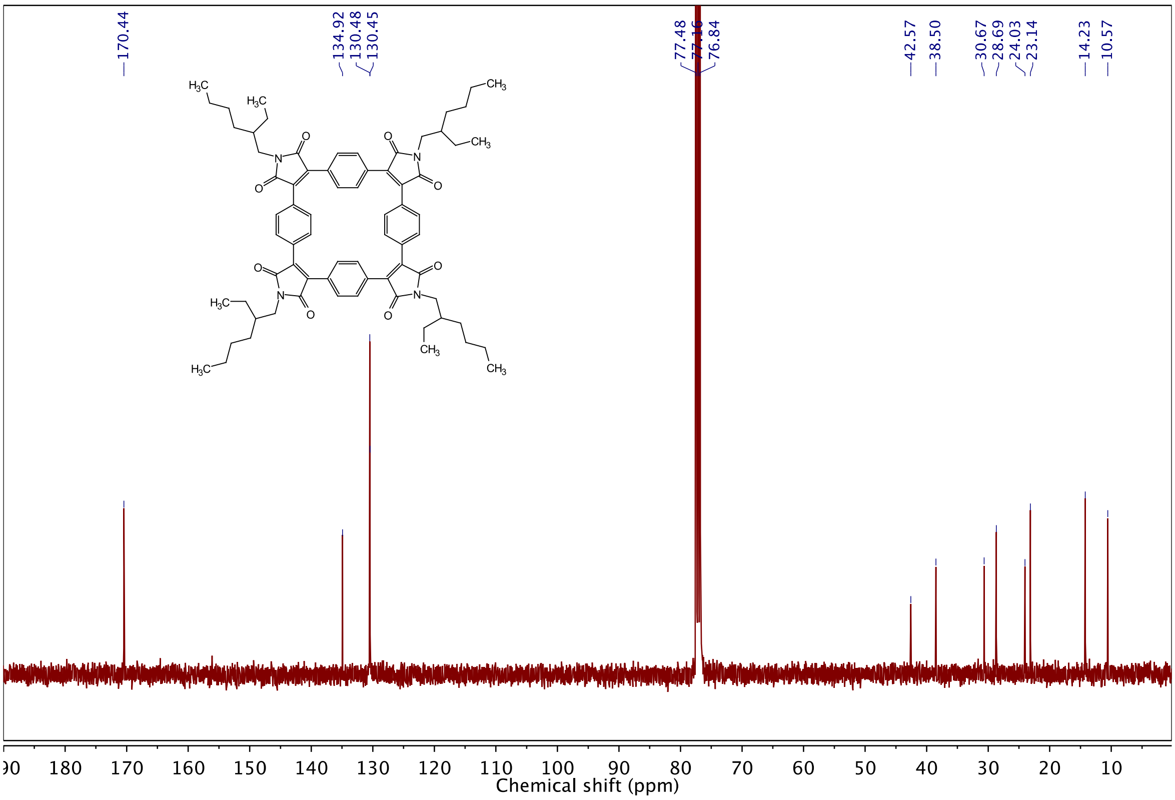

Figure S15. ^{13}C NMR (101 MHz, $CDCl_3$) of compound **SqTI-EtHx**.

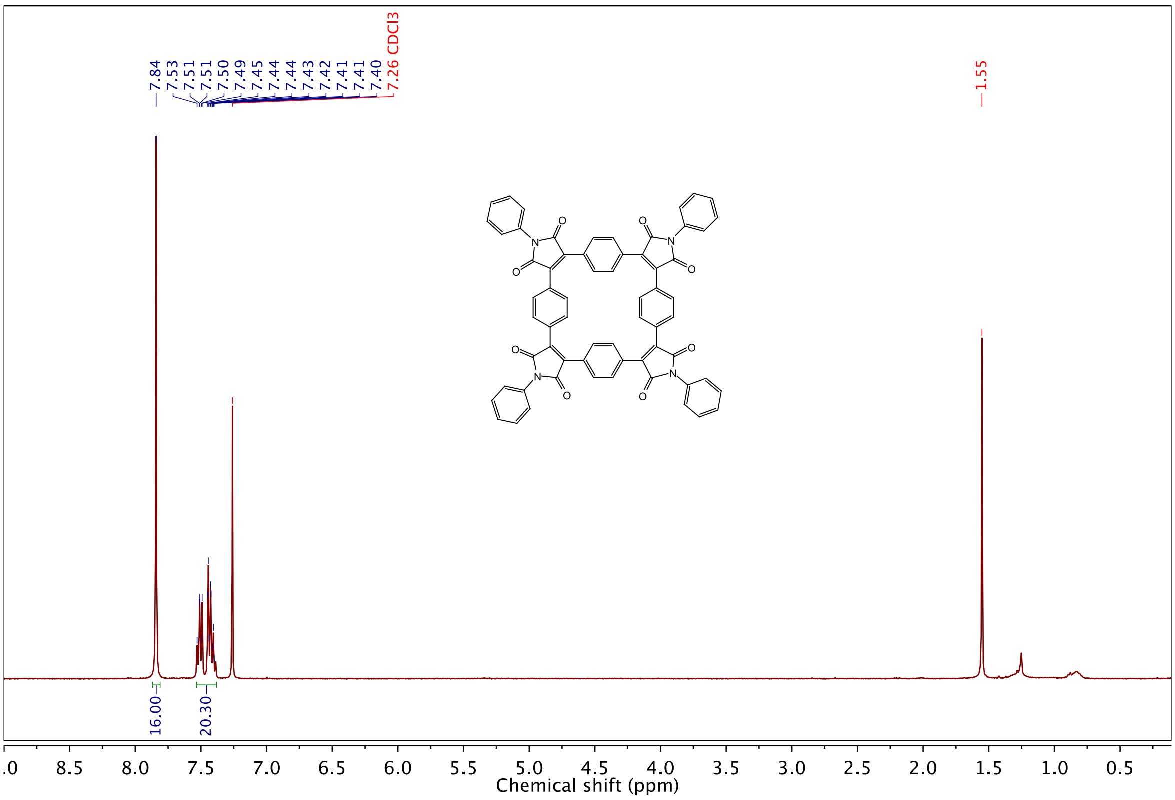

Figure S16. ^{1}H NMR (400 MHz, $CDCl_3$) of compound **SqTI-Ph**.

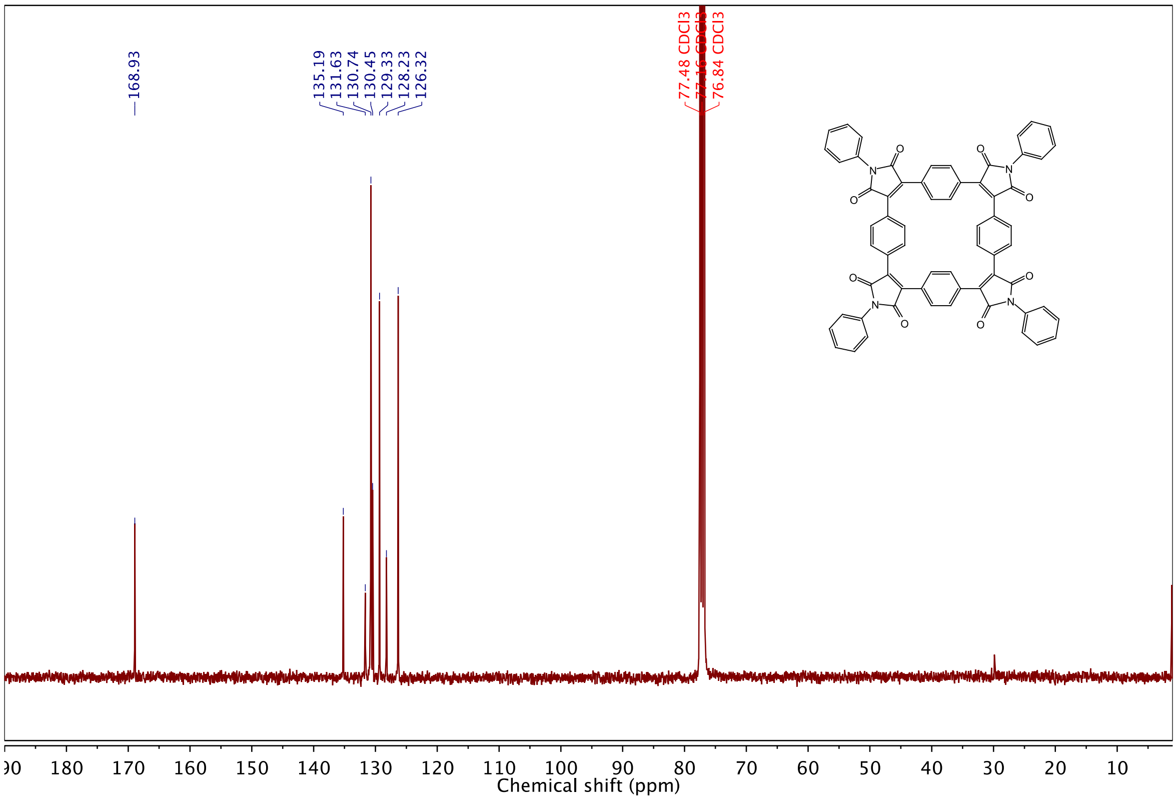

Figure S17. ^{13}C NMR (101 MHz, $CDCl_3$) of compound **SqTI-Ph**.

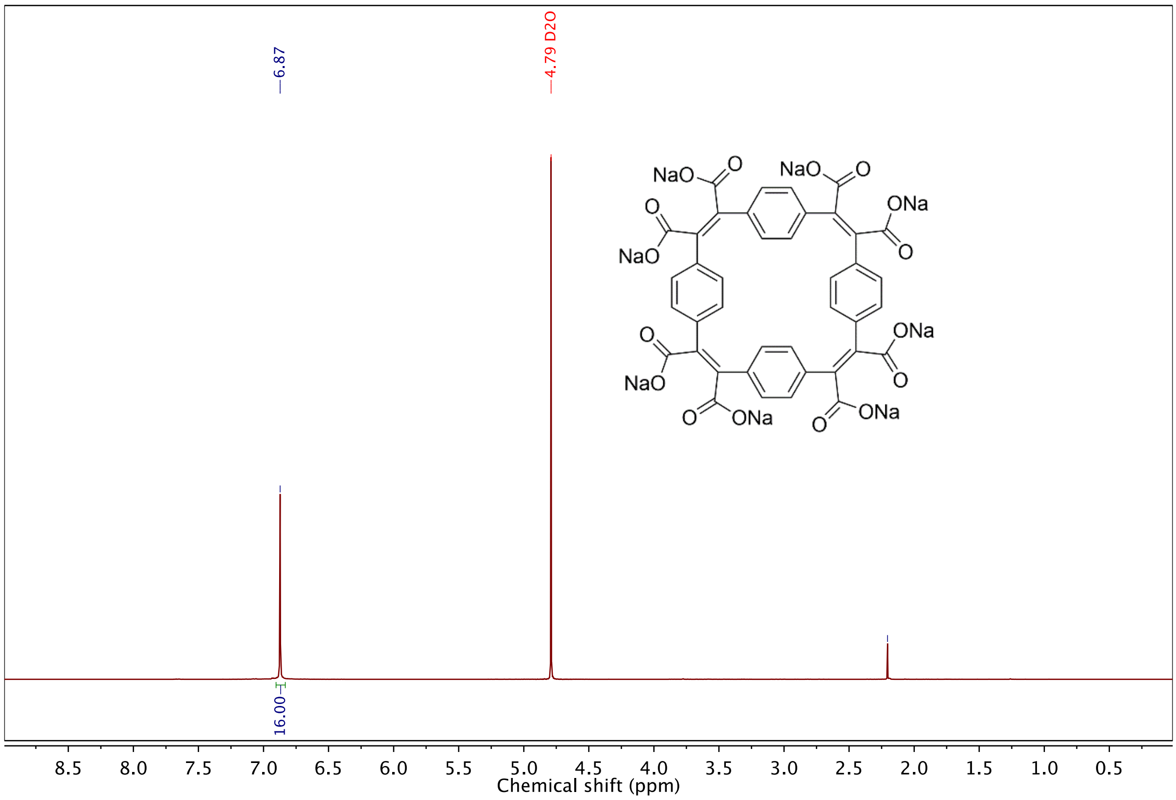

Figure S18. ^{1}H NMR (400 MHz, D_2O) of compound **SqNa.**

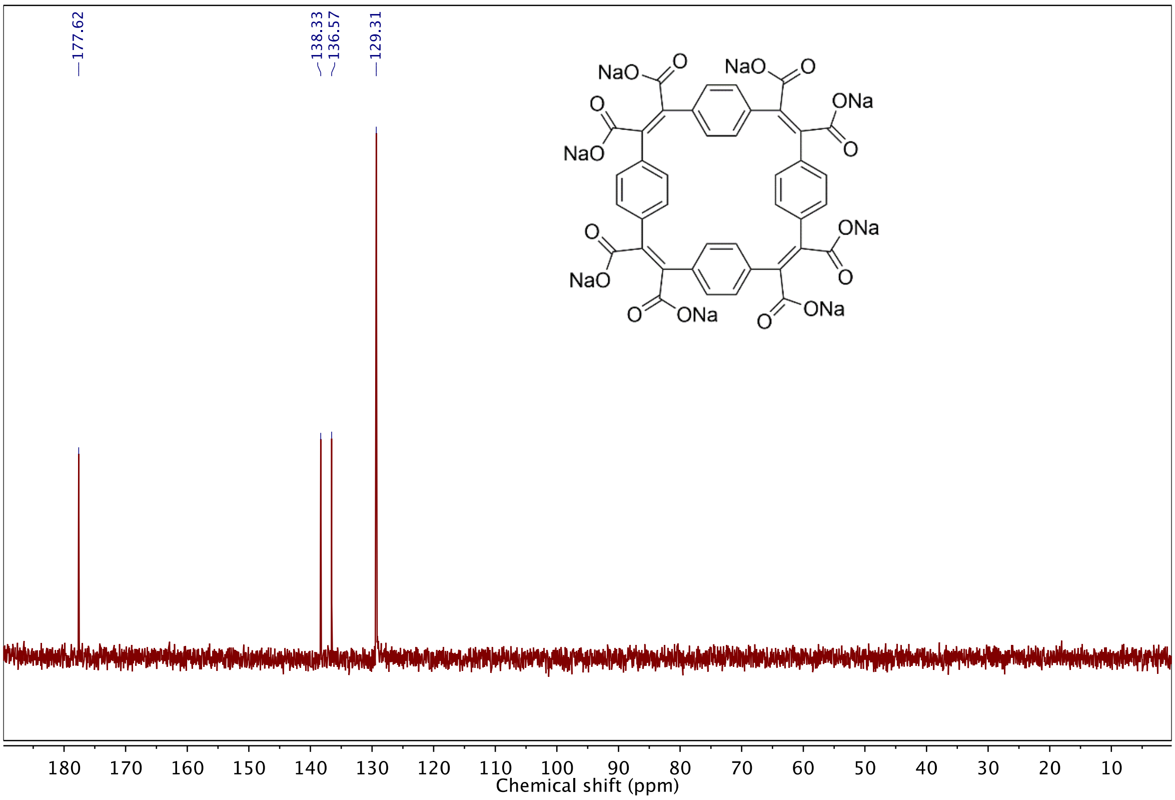

Figure S19. ^{13}C NMR (101 MHz, D_2O) of compound **SqNa.**

3. Crystallography

3.1 X-ray crystal structure of SqTA

Crystal data for **SqTA**:
$C_{40}H_{16}O_{12}\cdot 2(C_3H_6O)$, M = 804.68, monoclinic, $P2_1/n$ (no. 14), a = 10.9218(8), b = 19.4237(11), c = 18.3921(11) Å, β = 100.373(6)°, V = 3837.9(4) Å^3, Z = 4, D_c = 1.393 g cm^{-3}, μ(Mo-Kα) = 0.104 mm^{-1}, T = 173 K, orange blocks, Agilent Xcalibur 3 E diffractometer; 9209 independent measured reflections (R_{int} = 0.0282), F^2 refinement,[6] R_1(obs) = 0.0467, wR_2(all) = 0.1208, 5145 independent observed absorption-corrected reflections [$|F_o| > 4\sigma(|F_o|)$, completeness to θ_{full}(25.2°) = 98.2%], 580 parameters. CCDC 2170729.

The crystal of **SqTA** that was studied was found to be a two component twin in a *ca*. 69:31 ratio, with the two lattices related by the approximate twin law [–1.00 0.00 0.00 0.00 –1.00 0.00 0.61 0.00 1.00]. The O60-based included acetone solvent molecule was found to be disordered. Three orientations were identified of *ca*. 52, 28 and 20% occupancy, their geometries were optimised, the thermal parameters of adjacent atoms were restrained to be similar, and only the non-hydrogen atoms of the major occupancy orientation were refined anisotropically (those of the minor occupancy orientations were refined isotropically).

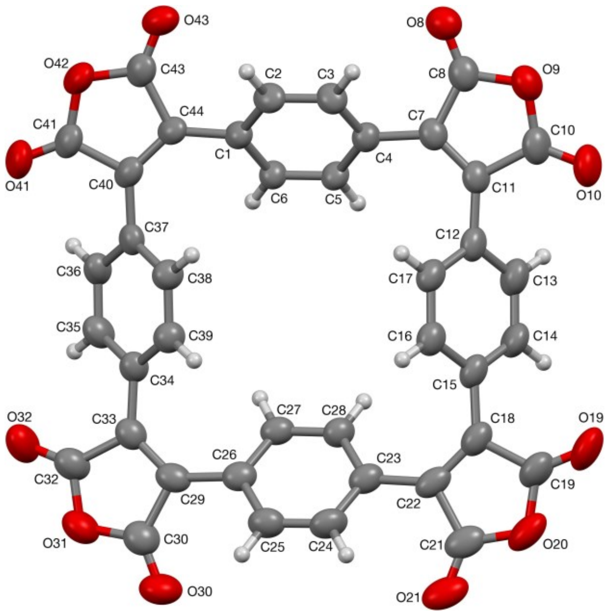

Figure S20. The crystal structure of **SqTA** (50% probability ellipsoids).

3.2 X-ray crystal structure of SqTI-Bu

Crystal data for **SqTI-Bu**:

$C_{56}H_{52}N_4O_8$, M = 909.01, monoclinic, $I2/a$ (no. 15), a = 19.8350(4), b = 13.6932(4), c = 17.5207(3) Å, β = 99.042(2)°, V = 4699.57(19) Å^3, Z = 4 [C_2 symmetry], D_c = 1.285 g cm^{-3}, μ(Cu-Kα) = 0.697 mm^{-1}, T = 173 K, orange blocky needles, Agilent Xcalibur PX Ultra A diffractometer; 4536 independent measured reflections (R_{int} = 0.0254), F^2 refinement,[6] R_1(obs) = 0.0480, wR_2(all) = 0.1507, 3375 independent observed absorption-corrected reflections [$|F_o| > 4\sigma(|F_o|)$, completeness to θ_{full}(67.7°) = 98.8%], 317 parameters. CCDC 2170730.

The structure of **SqTI-Bu** was found to sit across a C_2 axis that passes through the middle of the macrocyclic ring and is perpendicular to the mean plane of the four nitrogen atoms. The terminal ethyl portion of the N18-bound *n*-butyl group was found to be disordered. Two orientations were identified of *ca*. 68 and 32% occupancy, their geometries were optimised, the thermal parameters of adjacent atoms were restrained to be similar, and only the non-hydrogen atoms of the major occupancy orientation were refined anisotropically (those of the minor occupancy orientation were refined isotropically).

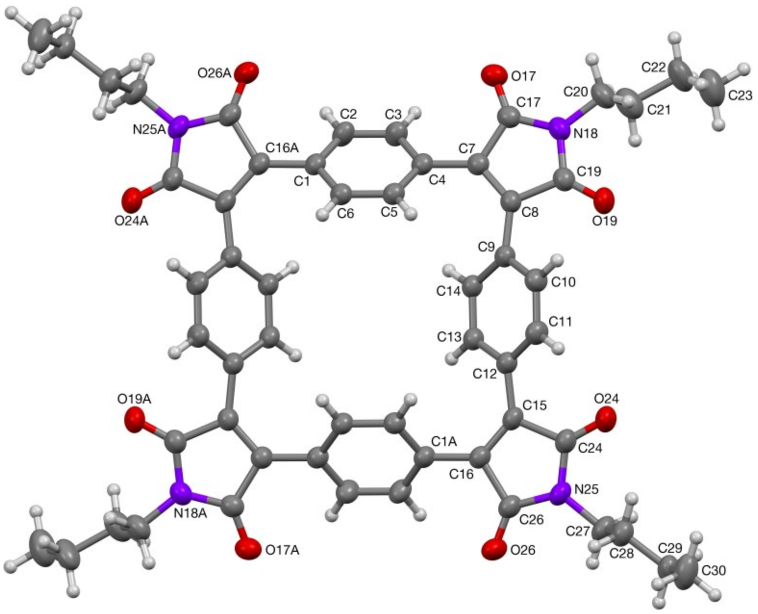

Figure S21. The crystal structure of the C_2-symmetric macrocycle **SqTI-Bu** (50% probability ellipsoids).

3.3 Void analysis

The analysis was performed using the void analysis tool in Mercury 2022.1.0 (Build 343014). The grid spacing was set to 0.1 Å and the probe radius was set to (i) 1.02 Å to identify empty space large enough to hold sodium ions or (ii) 0.76 Å to identify empty space large enough to hold lithium ions.

For the analysis of the crystal structure of **SqTA**, full occupancy in the major orientation was assumed for the disordered acetone inclusions. For the analysis of the crystal structure of **SqTI-Bu**, only the major orientation of the disordered terminal ethyl portion of the *n*-butyl groups was considered.

Naphthalenetetracarboxylic dianhydride (CCDC 129443) and naphthalenetetracarboxylic diimide with two n-butyl groups (CCDC 819749) were analysed for comparison with **SqTA** and **SqTI-Bu**, respectively.

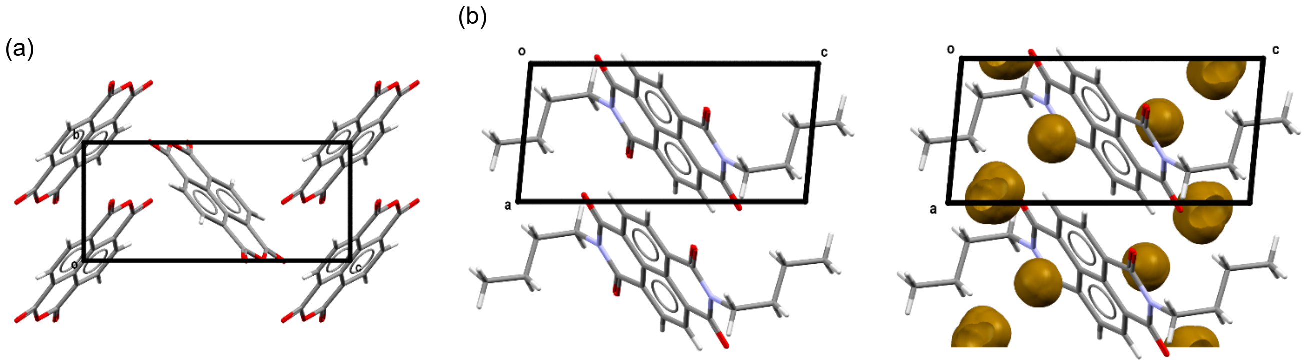

Figure S22. (a) Unit cell of naphthalenetetracarboxylic dianhydride (CCDC 129443); no voids large enough to hold sodium or lithium ions were identified. (b) Unit cell of naphthalenetetracarboxylic diimide with two *n*-butyl groups (CCDC 819749); no voids large enough to hold sodium ions were identified (left); only 3.9% (17.09 Å^3) of the unit cell volume were found to be empty space large enough to hold lithium ions (right).

4. Electrochemical measurements

4.1 Cyclic voltammetry in solution

Cyclic voltammetry (CV) measurements in solution were performed in 0.1 M [*n*-Bu_4N]PF_6/DMF with a Metrohm Autolab PGSTAT101 Electrochemical Analyser interfaced to NOVA software. A one-compartment three-electrode electrochemical cell was used for all measurements, featuring a 3 mm diameter glassy carbon working electrode, an Ag/Ag^+ non-aqueous reference electrode and a Pt counter electrode. Residual oxygen was removed saturating the electrolyte with dried N_2 by bubbling for 20 min prior to each measurement. Ferrocene was added as an internal reference upon completion of sample measurements.

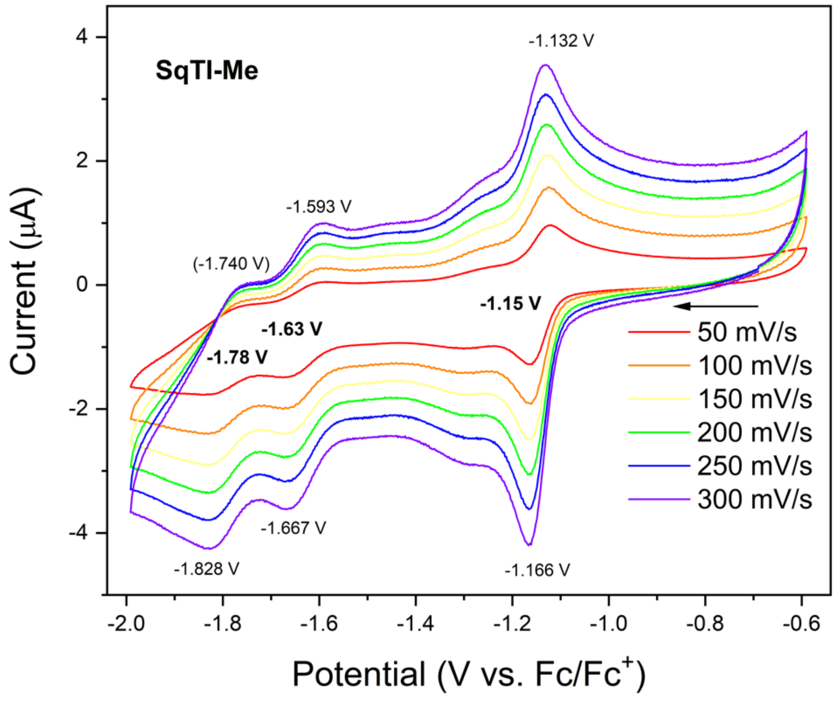

Figure S23. Cyclic voltammogram of **SqTI-Me** recorded in 0.1 M [*n*-Bu_4N]PF_6/DMF at different scan rates.

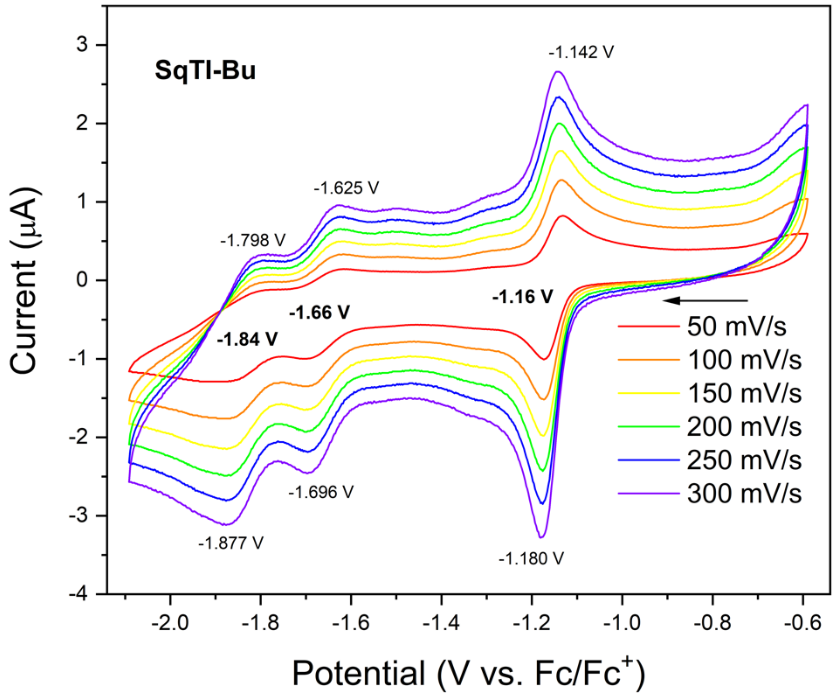

Figure S24. Cyclic voltammogram of **SqTI-Bu** recorded in 0.1 M [*n*-Bu_4N]PF_6/DMF at different scan rates.

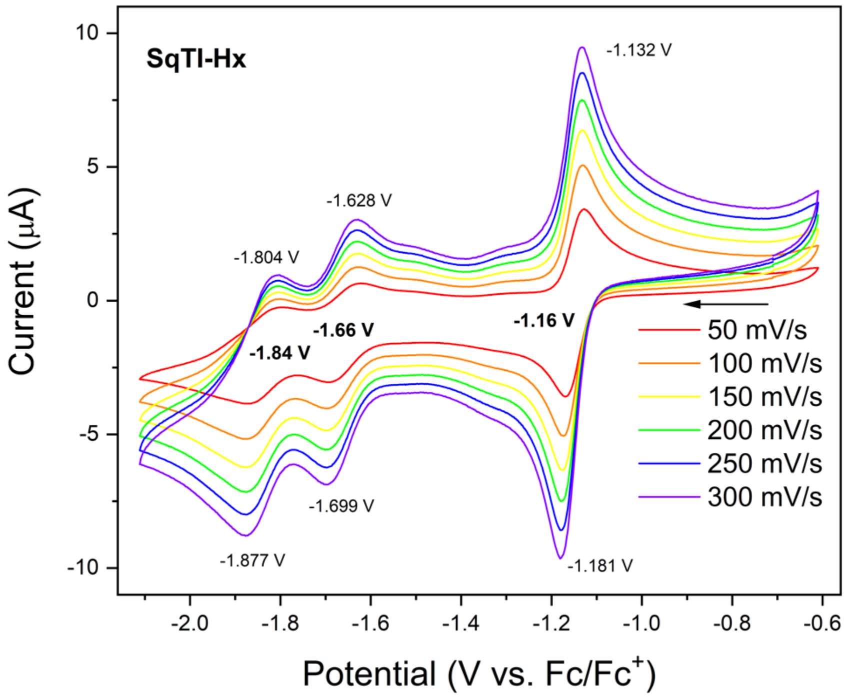

Figure S25. Cyclic voltammogram of **SqTI-Hx** recorded in 0.1 M [*n*-Bu_4N]PF_6/DMF at different scan rates.

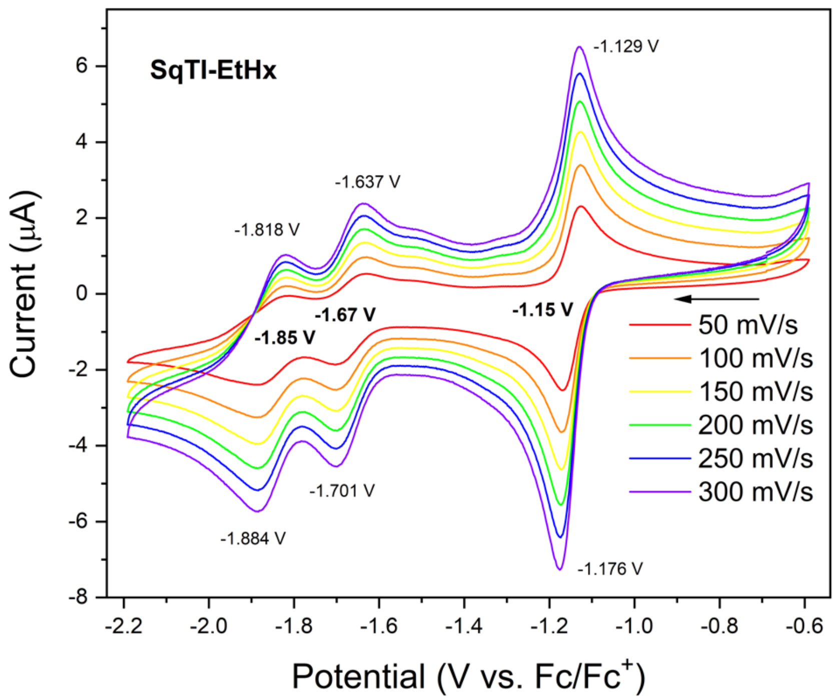

Figure S26. Cyclic voltammogram of **SqTI-EtHx** recorded in 0.1 M [*n*-Bu_4N]PF_6/DMF at different scan rates.

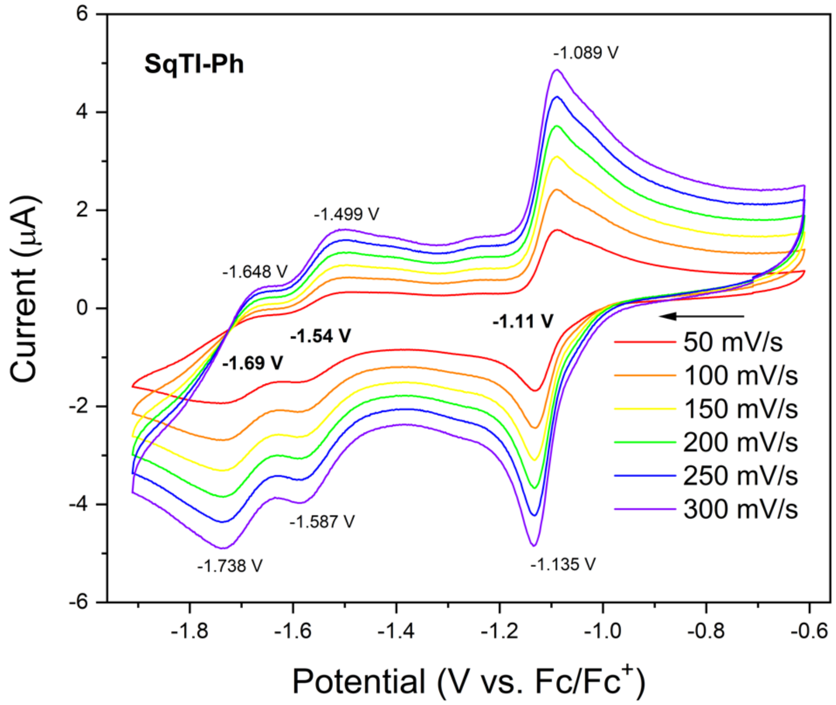

Figure S27. Cyclic voltammogram of **SqTI-Ph** recorded in 0.1 M [*n*-Bu_4N]PF_6/DMF at different scan rates.

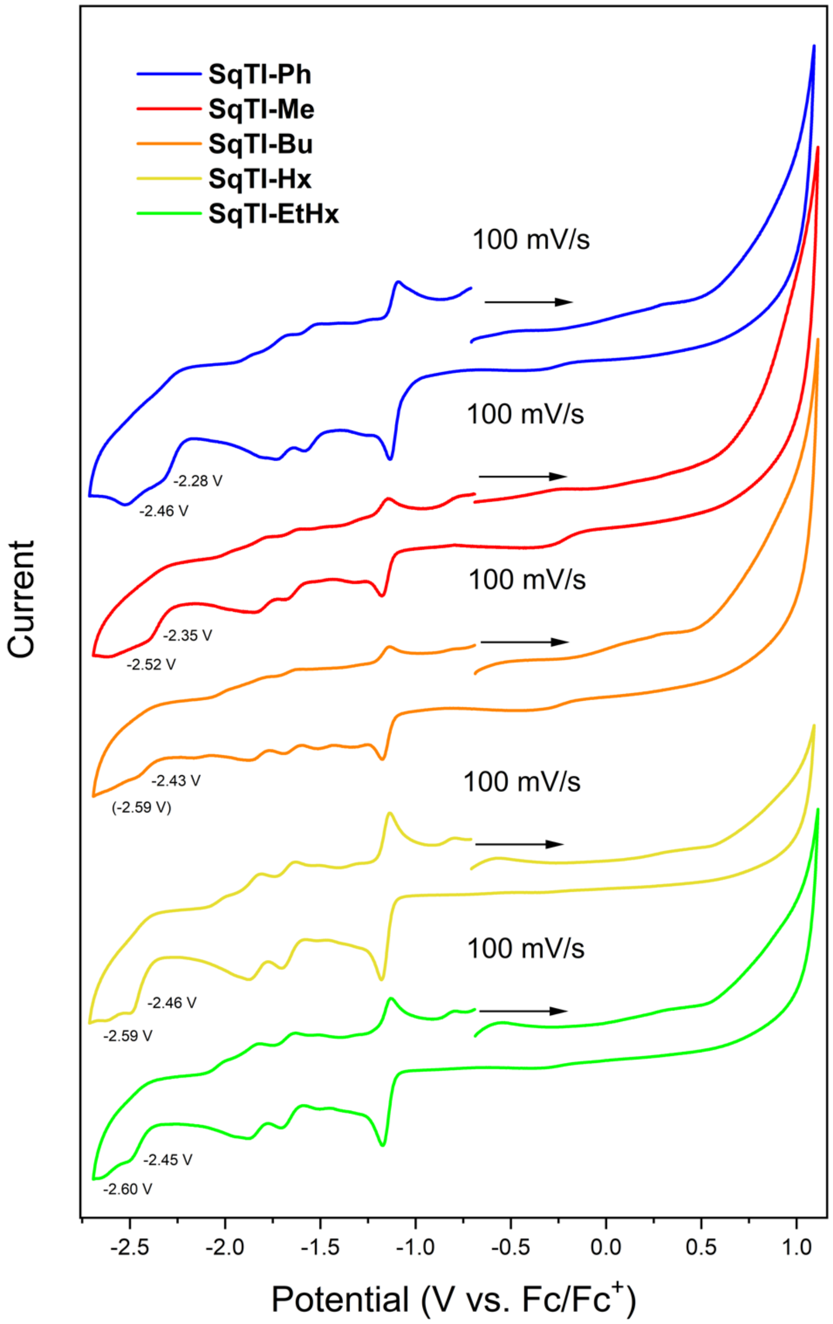

Figure S28. Cyclic voltammograms of all five squarephaneic tetraimides (**SqTI-R**) recorded in 0.1 M [*n*-Bu_4N]PF_6/DMF for a larger potential range. The voltammograms show further reduction steps at very low potential.

Table S1. Redox potentials vs. Fc/Fc^+ determined from the reduction waves in the cyclic voltammograms shown in Figures S23-29. The difference between the cathodic and anodic peak potential (ΔE_p) for the first reduction wave is also provided.

	1st wave	ΔE_p (1st wave)	2nd wave	3rd wave	4th wave	5th wave
**SqTI-Me**	-1.15 V	34 mV	-1.63 V	-1.78 V	-2.35 V	-2.52 V
**SqTI-Bu**	-1.16 V	38 mV	-1.66 V	-1.84 V	-2.43 V	-2.59 V
**SqTI-Hx**	-1.16 V	49 mV	-1.66 V	-1.84 V	-2.46 V	-2.59 V
**SqTI-EtHx**	-1.15 V	47 mV	-1.67 V	-1.85 V	-2.45 V	-2.60 V
**SqTI-Ph**	-1.11 V	46 mV	-1.54 V	-1.69 V	-2.28 V	-2.46 V

4.2 Measurements in the solid state

Electrodes were prepared by stirring 50 wt% active material, wt30% carbon black (C-NERGY C45) and wt20% polyvinylidene fluoride (Sigma Aldrich) in 1-Methyl-2-pyrrolidinone (Sigma Aldrich). The slurry was then cast onto a copper foil (Goodfellow) with a doctor blade at a thickness of 50 µm. The electrode was first dried on a hotplate and then under vacuum overnight at 120 °C. 18 mm electrode disks were punched out with a hand-held disk cutter (MTI) and assembled into coin cells (MTI casings and crimper). The electrodes were then cycled (Biologic battery tester) against lithium metal in the coin cells with LP57 (1 M $LiPF_6$ in EC/EMC (3:7)) electrolyte and opened for post-mortem analysis with a decrimper (Hohsen).
A cyclic voltammogram between 0.01 and 3.5 V vs. Li/Li^+, measured at a scan rate of 1 mVs^{-1}, is shown in Figure S29. The slow scan rate was chosen to facilitate the probability of lithium intercalation rather than plating, while the potential rage was chosen in order to investigate the full range of intercalation, above and below the open circuit potential. In the anodic scan of the first cycle, two sharp redox peaks were observed at approximately 1.8 and 1.4 V vs. Li/Li^+. The corresponding deintercalation peaks are observed at about 2.7 and 3.2 V vs. Li/Li^+, although at lower currents. The hysteresis observed suggests a significant increase in the cell's resistance. The anodic peak at ~ 0 V vs. Li/Li+ and the cathodic peak at ~ 0.5 V vs. Li/Li^+ result from the plating and stripping of lithium metal on the electrode. A capacitive contribution to the current from the presence of carbon black[7] is also noted at around 1.0 V vs. Li/Li^+ in both the cathodic and anodic scans. Upon continued cycling, the redox peaks attributed to the (de)intercalation of lithium ions into **SqTI-Bu** decrease in size, explained by dissolution of the active material in the electrolyte and migration through the separator (see below for details).

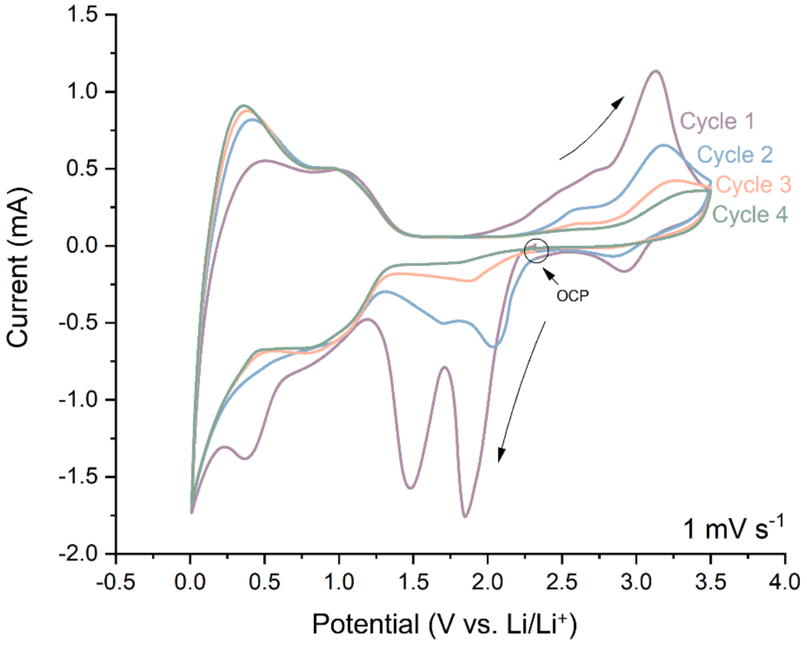

Figure S29. Cyclic voltammogram of **SqTI-Bu** vs. lithium metal in a coin cell between 0.01 and 3.5 V vs. Li/Li^+ measured at a scan rate of 1 mVs^{-1} for four cycles.

In order to solely investigate the redox peaks of interest, another cell was cycled at 1 mVs^{-1} within a restricted potential range, from 1.4 to 2.6 V vs. Li/Li^+ (Figure S30). Again, distinctive redox peaks were observed in the first anodic scan, attributed to lithium intercalation into **SqTI-Bu** but shifted to slightly higher potentials. The reverse peaks in the cathodic scan again

see significantly smaller currents, but less hysteresis was observed in these measurements. This is likely due to the lack of polarisation developed through the formation of an SEI layer from reduced electrolyte products at low potentials.[8] Upon further cycling, the redox peaks continue to decrease in size, suggesting that less intercalation takes place and that restricting the potential range does not prevent the degradation of the electrode. It is also worth noting that in the second cycle, three distinctive peaks may be noticed, and that looking back to the first cycles in Figures S29 and S30, contribution from this third peak may also be noted in the large anodic peak at lower potentials. The presence and positions of these three peaks is in good agreement with the findings of the electrochemical measurements in solution.

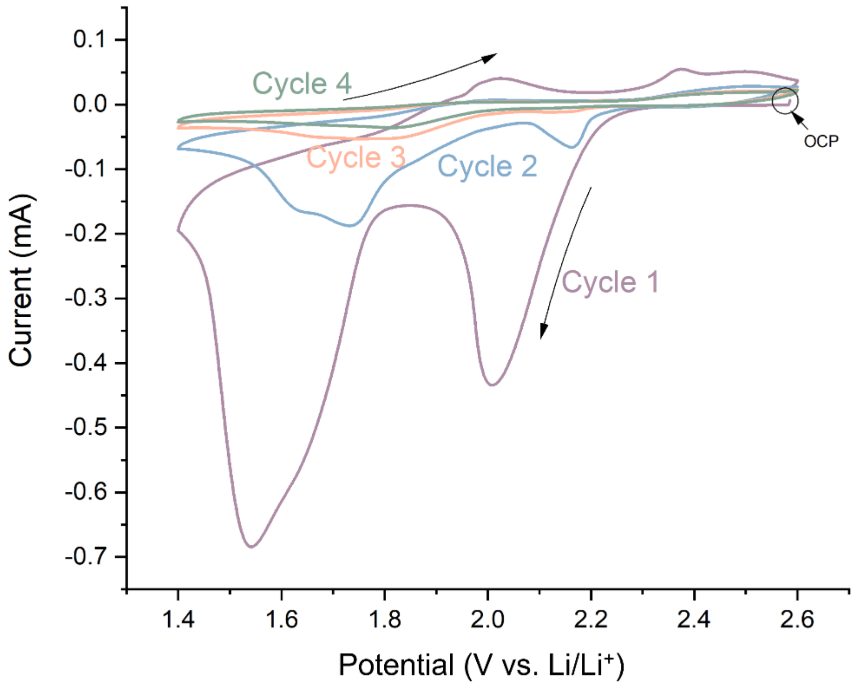

Figure S30. Cyclic voltammogram of **SqTI-Bu** vs. lithium metal in a coin cell in a smaller potential range of 1.4 and 2.5 V vs. Li/Li$^+$ measured at a scan rate of 1 mV s^{-1} for four cycles.

To further investigate the performance of **SqTI-Bu** as a lithium-ion battery anode material and the cause of the diminished intercalation on continued cycling, an electrode was cycled with a constant current between 2.5 and 0.01 V vs. Li/Li$^+$ at a rate of C/4 and the corresponding potential vs. capacity plot can be seen in Figure S31. In the first cycle, at least three potential plateaus were observed, contributing to significant capacity. Upon subsequent cycling, these plateaus are no longer observed, indicating the irreversibility of the discharging and charging process in constant current mode. To investigate the cause of this irreversibility, the coin cells were opened and taken apart after cycling. The coin cell separator had become orange in colour (Figure S32), the same colour as the active material powder, suggesting dissolution of the active material and migration through the separator. This dissolution likely explains the diminished performance upon increased cycle number.

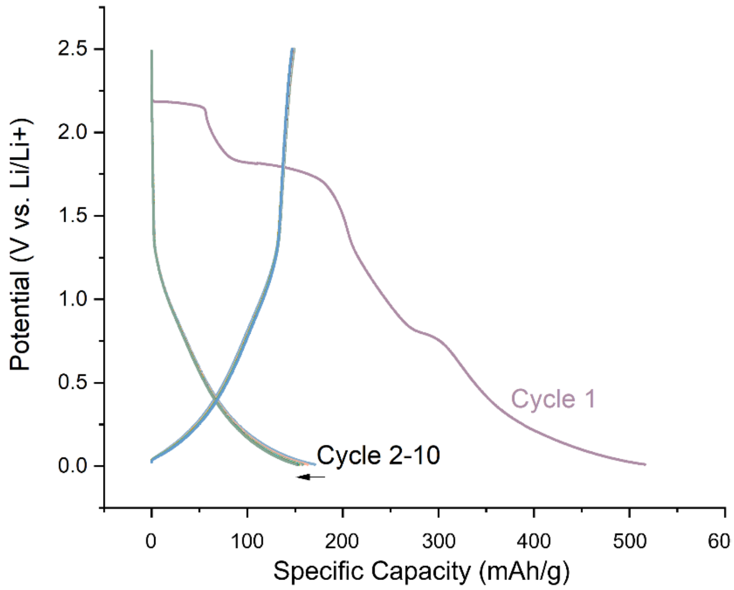

Figure S31. A potential versus specific capacity plot of **SqTI-Bu** vs. lithium metal in a coin cell cycled in a potential range of 0.01 and 2.5 V vs. Li/Li^{+} measured at C-rate of C/4 for 10 cycles.

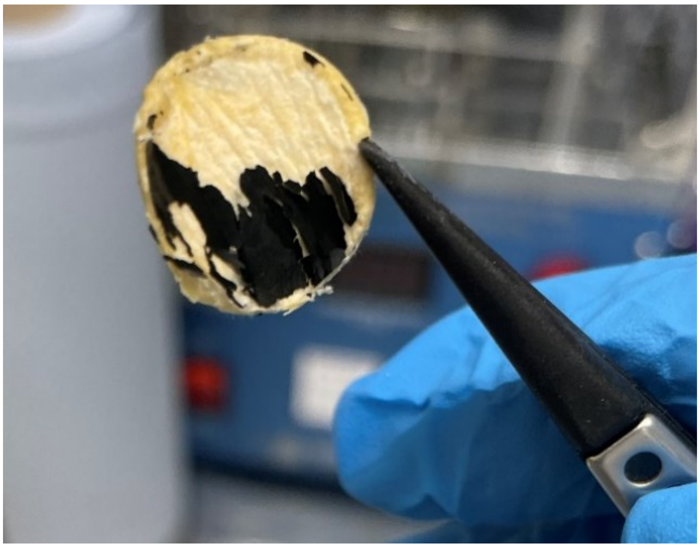

Figure S32. An image of the separator from a disassembled cycled **SqTI-Bu** coin cell. The orange colour of the active material can clearly be seen to have dissolved into the separator and soaked through the separator.

5. Computational analysis

Computations considered singlet states of charge 0, -1, -2, -3, -4, and -6 as well as triplet states of charge 0 and -4. Geometries for these electronic states were optimized in vacuum using the PBE0 functional[9] along with the def2-SV(P) basis set[10] and the D3 dispersion correction[11] in its optimized power parameterization.[12] For singlet states, spin-restricted Kohn-Sham (RKS) computations were performed, whereas for doublets and triplets an unrestricted (UKS) formalism was employed. Formal $<S^2>$ values indicating spin-contamination are presented in Section 5.2. Vibrational frequency analyses at the same level were performed to verify the nature of the stationary points as minima and to obtain enthalpy and entropy corrections for the redox potentials. Additional single-point computations were performed at the PBE0-D3/def2-SVPD level including solvation effects using the SMD model of Truhlar and co-workers[13] to represent DMF.

The redox potential for any given redox couple z_1/z_2 was computed as

$$E(z_1/z_2) = \frac{\mathrm{G}(z_2) - \mathrm{G}(z_1)}{e \times (z_2 - z_1)} - E_{\mathrm{ref}}$$

where $G(z)$ is the free energy, e is the unit charge and E_{ref} is the absolute potential of the reference electrode (cf. Ref. [14]). The free energy was determined from PBE0-D3(SMD)/def2-SVPD single-point computations along with enthalpy/entropy corrections determined from vibrational analyses at the PBE0-D3/def2-SV(P) level in gas phase. A value of 4.70 V was used for E_{ref}. For **SqTA**, **SqTI-Bu**, and **SqTI-Ph**, the redox potentials reported pertain to the redox couples (0/-1), (0/-2), (-2/-3), (-3/-4), and (-4/-6), considering generally states of singlet spin-multiplicity and for the tetraanions also triplet multiplicity. For **SqNa**, the redox potentials reported pertain to the redox couples (0/-1), (-1/-2), and (-2/4), considering only states of singlet spin-multiplicity. These calculations were carried out in Q-Chem 5.[15]
Nucleus independent chemical shifts (NICS)[16] were computed at the PBE0/def2-SVP level using gauge including atomic orbitals[17] as implemented in Gaussian 09.[18] NICS tensors were represented graphically using the VIST (visualisation of chemical shielding tensors) method[19] as implemented in TheoDORE 3.0[20] and using VMD as a graphical interface.[21]
Changes in the electronic density upon addition of electrons were modelled using Head-Gordon's attachment-detachment analysis.[22] This analysis was performed using the "analyze_nos" functionality of TheoDORE 3.0 using the molecular orbitals of the charged and neutral states (both computed at the geometry of the charged state) as input. Only contributions from natural difference orbitals with eigenvalues above 0.2 were considered in the analysis to eliminate unwanted orbital relaxation contributions in the pictorial representation.
The underlying computational research data is available via a separate repository (DOI: 10.17028/rd.lboro.20180150): Q-Chem input/output files for geometry optimisations, frequency analyses and solvated single-point computations; Gaussian input/output files for NICS computations; TheoDORE files for VIST analysis in VMD.

5.1 Redox potentials

Table S2. Computed redox potentials vs. Fc/Fc^+ for the different redox couples of the macrocycles.

	0/-1	0/-2	-2/-3	-3/-4	-3/-4 (trip.)	-4/-6	-4 (trip.)/-6
**SqTA**	-0.62	-0.80	-1.48	-1.82	-1.77	-2.37	-2.40
**SqTI-Bu**	-1.06	-1.18	-1.66	-2.31	-2.22	-2.65	-2.70
**SqTI-Ph**	-1.03	-1.13	-1.82	-2.09	-2.00	-2.65	-2.70
	**0/-1**	**-1/-2**	**-2/-4**				
**SqNa**	-2.33	-2.98	-4.16				

5.2 Spin-expectation values

Table S3 presents the formal spin expectation values computed for the UKS determinants in the doublet and triplet states. Values close to the idealized results (0.75 for doublets and 2.00 for triplets) are obtained in all cases except for the -3 state of **SqTI-Bu** where a slightly higher value is obtained.

Table S3. Formal spin-expectation values obtained in U-PBE0 computations in the different charge and spin states.

	-1 (doub.)	-3 (doub.)	0 (trip.)	-4 (trip.)
**SqTA**	0.759	0.778	2.004	2.016
**SqTI-Bu**	0.759	1.048	2.004	2.018
**SqTI-Ph**	0.759	0.781	2.004	2.018
**SqNa**	0.753			

5.3 Nucleus-independent chemical shifts (NICS)

Table S4. $NICS(0)_{ev}$ values of the macrocycles in the different charge and spin states.

	SqTA		SqTI-Bu		SqTI-Ph		SqNa
**Charge state**	**$NICS(0)_{ev}$ (ppm)**		**$NICS(0)_{ev}$ (ppm)**		**$NICS(0)_{ev}$ (ppm)**		**$NICS(0)_{ev}$ (ppm)**
	**singlet**	**triplet**	**singlet**	**triplet**	**singlet**	**triplet**	**singlet**
**0**	10.41	-26.28	11.02	-25.87	10.97	-24.29	6.20
**-1**	-3.52		-2.49		-2.08		-0.15
**-2**	-23.14		-22.02		-20.40		1.20
**-3**	21.16		3.58		18.13		
**-4**	58.70	-13.30	49.29	-10.91	53.97	-10.01	13.88
**-6**	-4.16		10.79		-1.85		

5.4 Visualisation of chemical shielding tensors (VIST)

Values shown in the plots give the chemical shielding of selected tensor components in ppm.

5.4.1 VIST plots of SqTA in different charge and spin states

SqTA

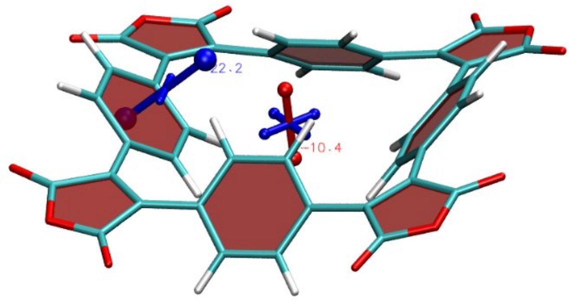

SqTA (triplet)

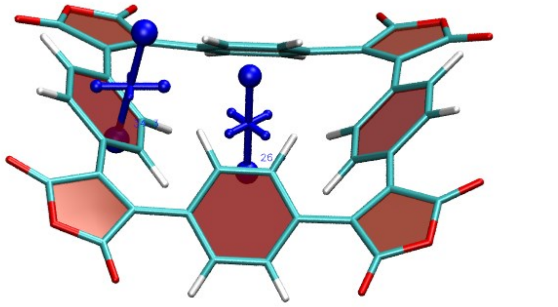

SqTA^{2-}

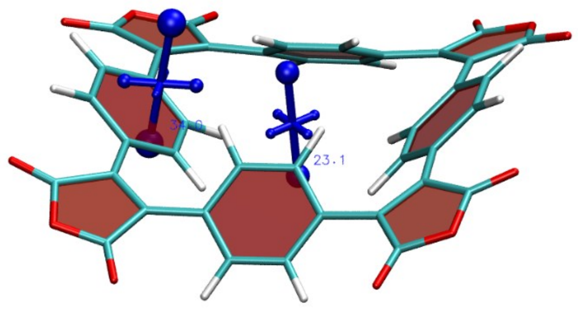

SqTA^{3-}

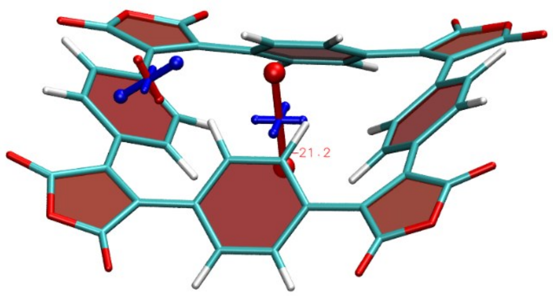

SqTA^{4-}

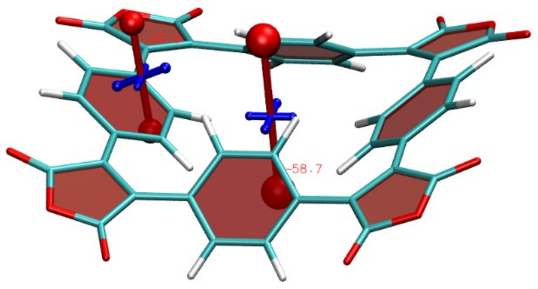

SqTA^{4-} (triplet)

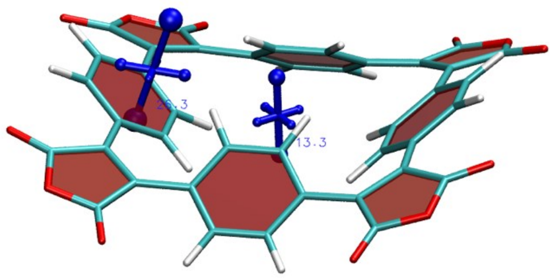

SqTA^{6-}

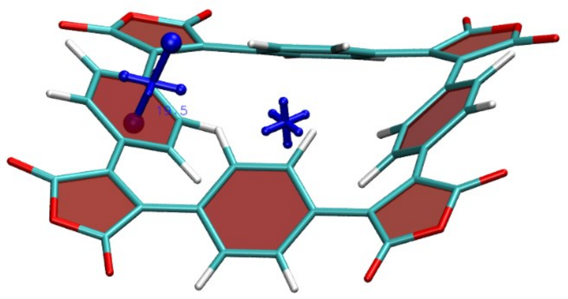

5.4.2 VIST plots of SqTI-Bu in different charge and spin states

SqTI-Bu

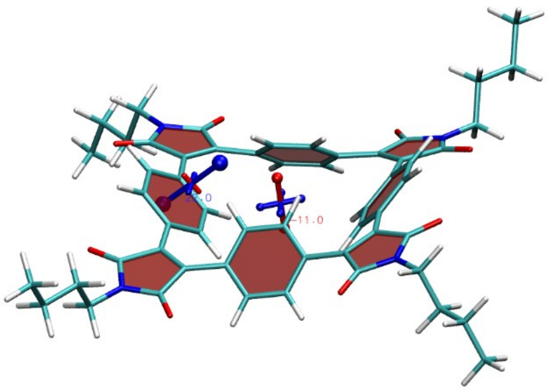

SqTI-Bu (triplet)

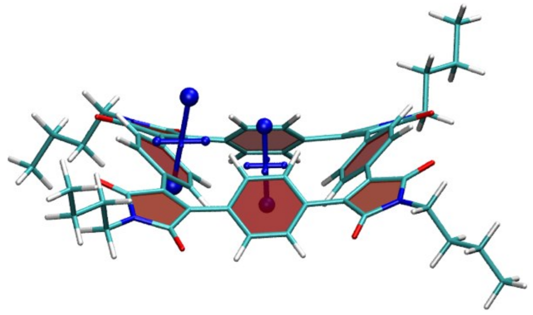

SqTI-Bu^{2-}

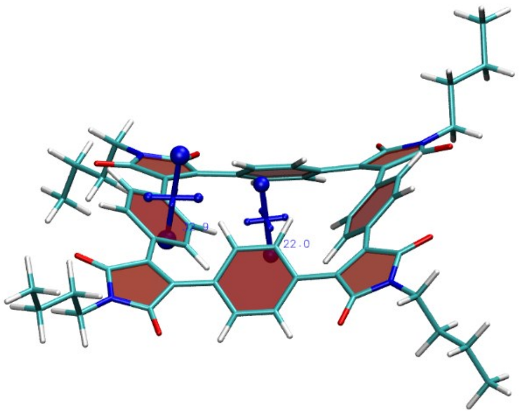

SqTI-Bu^{3-}

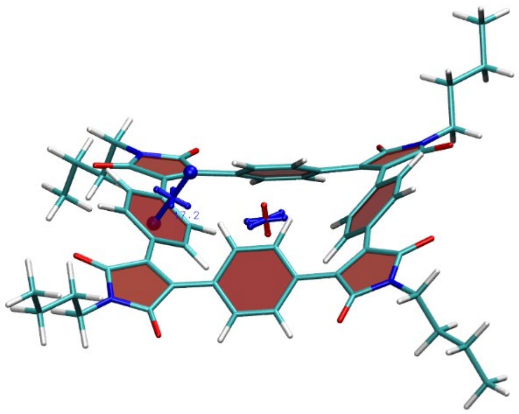

SqTI-Bu^{4-}

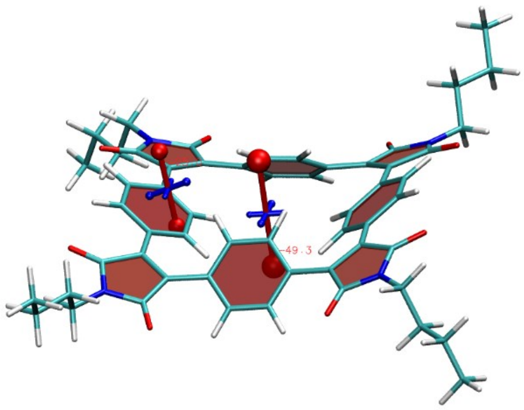

SqTI-Bu^{4-} (triplet)

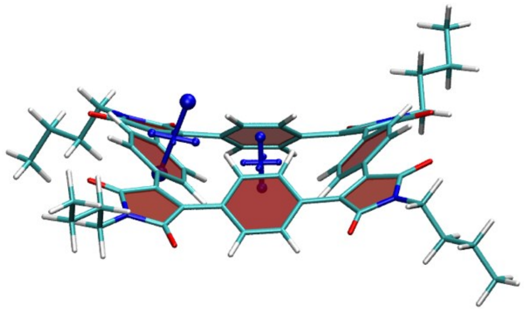

SqTI-Bu^{6-}

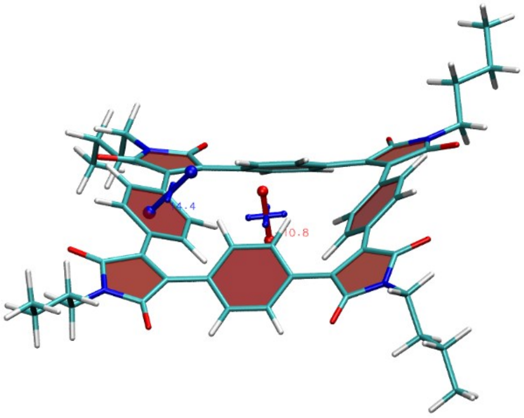

5.4.3 VIST plots of SqTI-Ph in different charge and spin states

**SqTI-Ph**	**SqTI-Ph (triplet)**
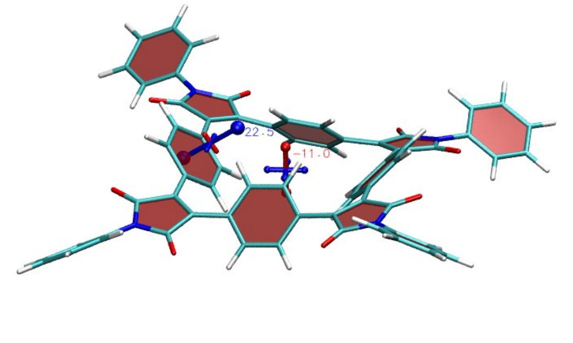	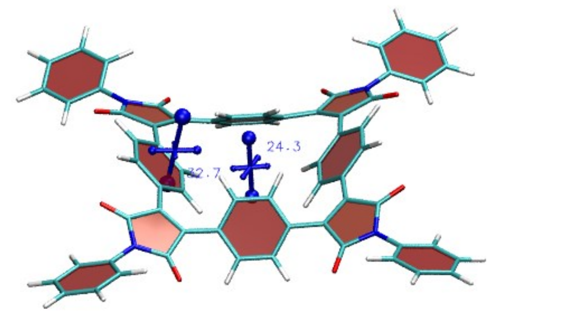
**SqTI-Ph^{2-}**	**SqTI-Ph^{3-}**
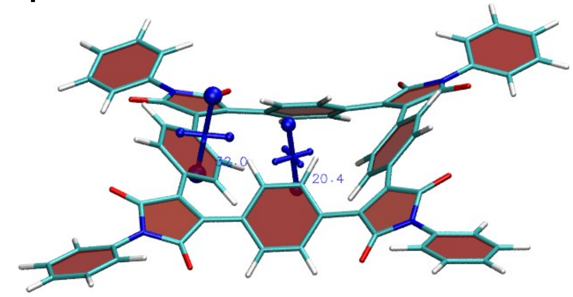	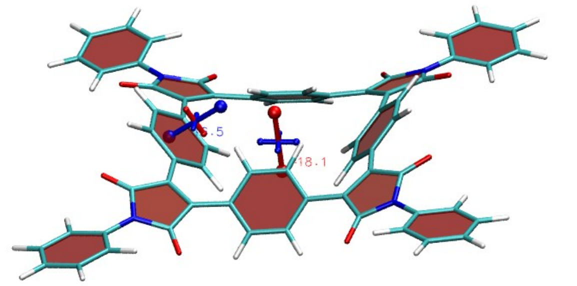
**SqTI-Ph^{4-}**	**SqTI-Ph^{4-} (triplet)**
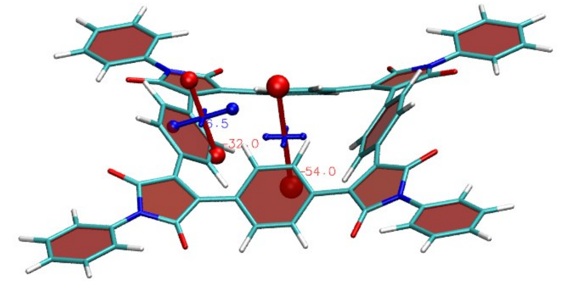	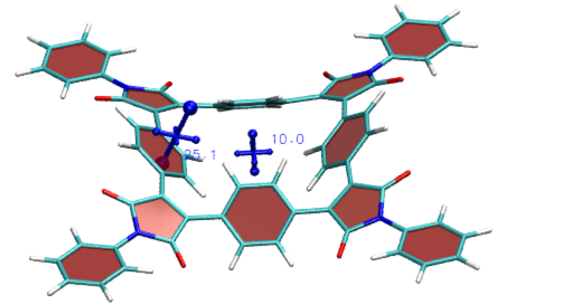
**SqTI-Ph^{6-}**	
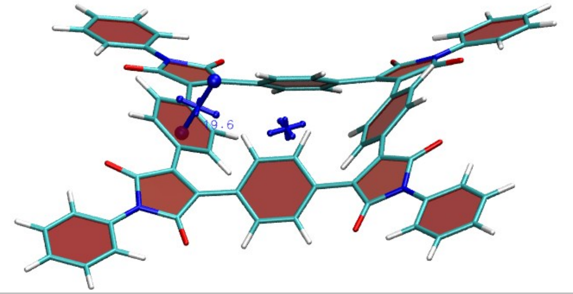	

5.4.4 VIST plots of SqNa in different charge

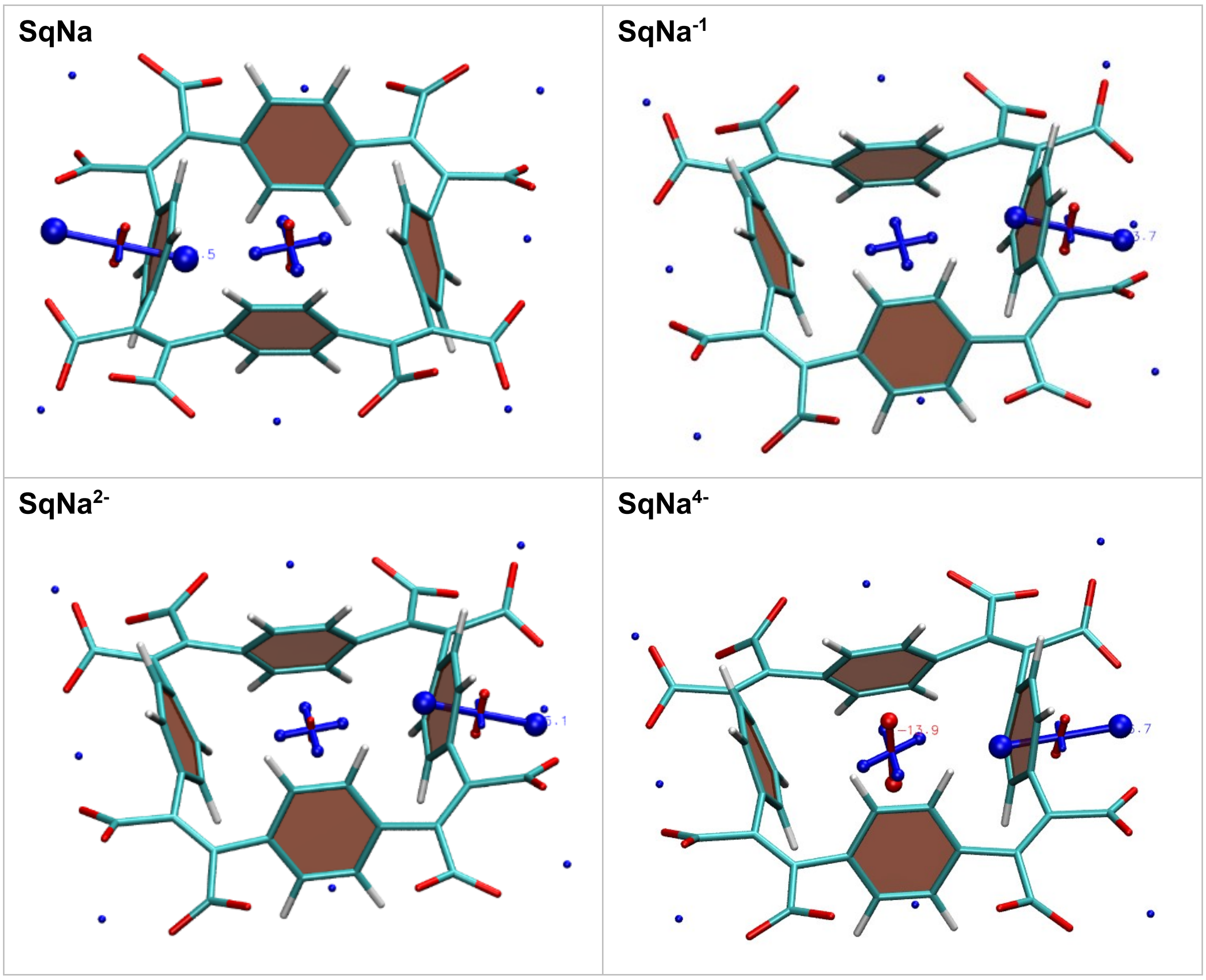

5.5 Electron density difference

Isovalue: -0.009 e

5.5.1 Attachment density plots for SqTI-Bu

SqTI-Bu^{2-}

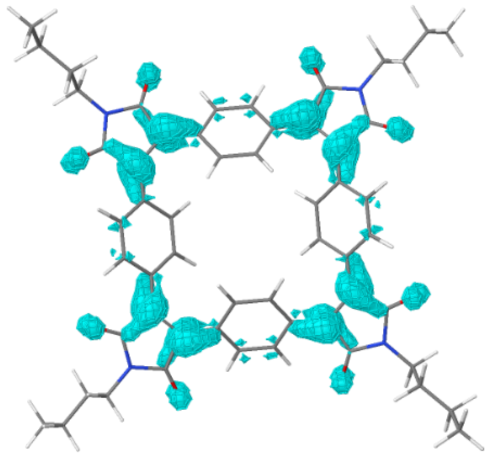

SqTI-Bu^{4-}

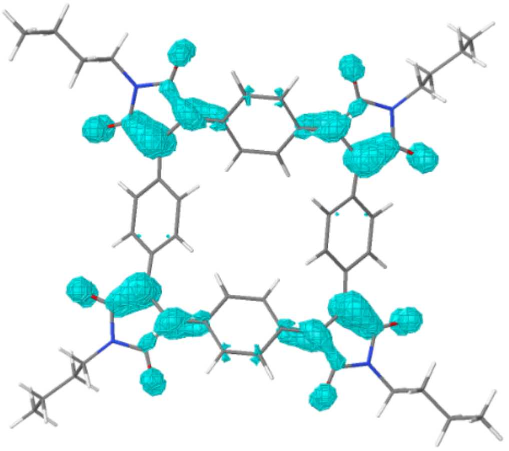

SqTI-Bu^{4-} (triplet)

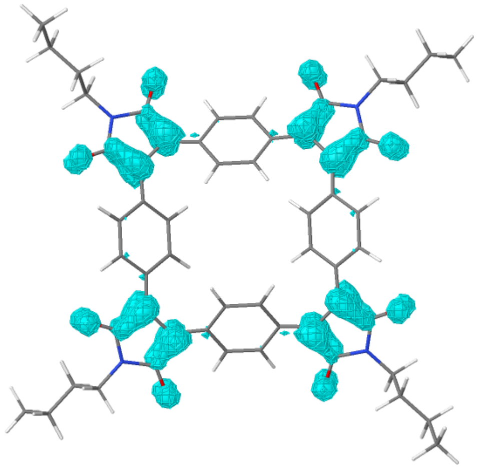

5.5.2 Attachment density plots for SqTI-Ph

SqTI-Ph^{2-}

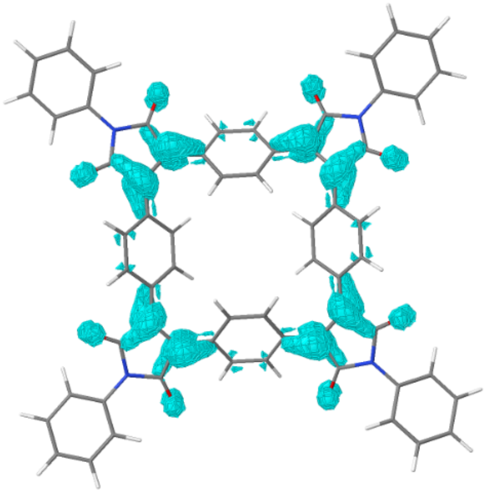

SqTI-Ph^{4-}

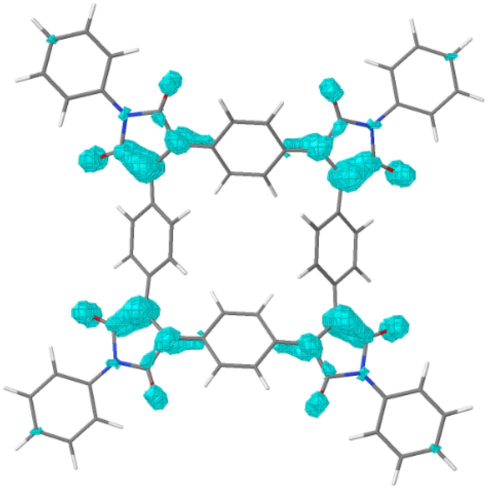

6. EPR spectroelectrochemical measurements

EPR spectroelectrochemical (SEC) data in the X-band was collected at room temperature using a Bruker EMX EPR spectrometer. The one compartment EPR SEC cell was constructed from a Pasteur pipette flame sealed at the thin end and filled to halfway with the sample dissolved in 0.1 M [n-Bu_4N]PF_6/DMF electrolyte (at *ca.* 1 mM). Three electrodes, which included a short bare Pt counter electrode, a medium length Teflon coated silver wire Ag/Ag^+ quasi-reference electrode, and a long Teflon coated Pt working electrode (WE), were connected to separate copper inserts wrapped in Teflon tape and sequentially inserted into the electrolyte to prevent short circuiting. The tip of the Pt wire WE was connected to a piece of Pt gauze onto which the EPR cell was centred about. The applied potential was controlled using a Biologic SP300 potentiostat and referenced against Fc/Fc^+ (by addition of ferrocene to the EPR SEC cell and CV measurements following the EPR measurements).

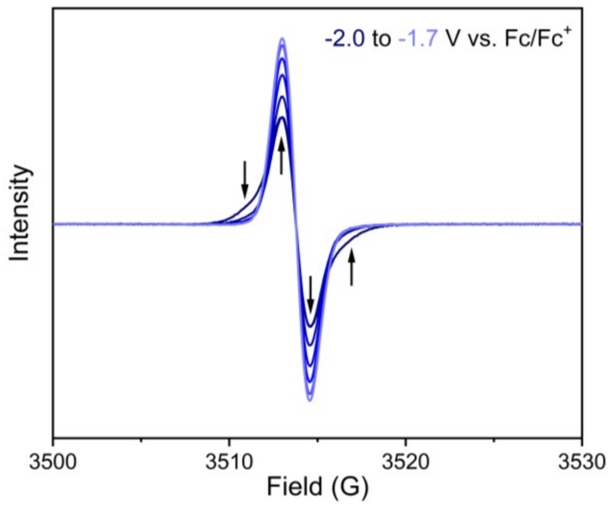

Figure S33. EPR spectroelectrochemical measurements of **SqTI-Hx** in 0.1 M [*n*-Bu_4N]PF_6/DMF showing the recovery of the doublet trianion organic radical signal upon returning the applied potential to -1.7 V.

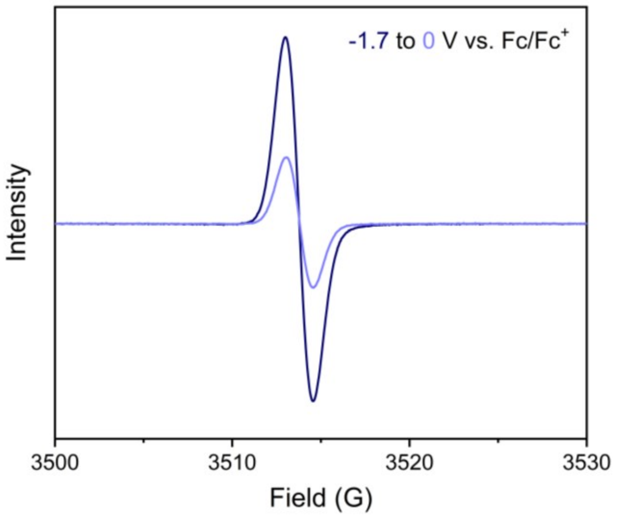

Figure S34. EPR spectroelectrochemical measurements of **SqTI-Hx** in 0.1 M [*n*-Bu_4N]PF_6/DMF showing the decay of the doublet radical signal as the applied potential was incremented back to 0 V.

7. Chemical reduction and low-temperature NMR measurements

7.1 Chemical reduction with cobaltocene

Chemical reduction of **SqTI-Hx** to **SqTI-Hx^{2-}** for NMR experiments was performed under nitrogen in a glovebox. Approx. 2 mg **SqTI-Hx** were dissolved in 0.6 mL CD_2Cl_2 (dried for >24 h using 3 Å molecular sieves) and 3 equiv. cobaltocene dissolved in 75 µL dry CD_2Cl_2 were added while stirring the **SqTI-Hx** solution. The resulting solution was transferred into an NMR tube fitted with a Young valve and transported from the glovebox to the NMR spectrometer, with the first measurement taken approx. 10 min after the addition of the cobaltocene solution. Figure S35 shows the NMR spectra of neutral **SqTI-Hx** in CD_2Cl_2 recorded at room temperature and 193 K at the top and the spectra of the reduced compound **SqTI-Hx^{2-}** at 193 K (two spectra of different experiments), 203 K, 213 K, and 223 K below. The reduction leads

to distinct changes in the spectrum, with the most pronounced change being the splitting of the signal of the phenylene-Hs into two signals (indicated by black lines), indicating the presence of a macrocyclic diatropic current. The peaks in grey boxes do not originate from **SqTI-Hx** or **SqTI-Hx^{2-}** but correspond to side products of the oxidation (related to cobaltocene/cobaltocenium) and to chloroform. These peaks (and further peaks overlapping with the aliphatic signals of the compound) are also present when measuring just the diluted cobaltocene solution without strict exclusion of air (Figure S36).

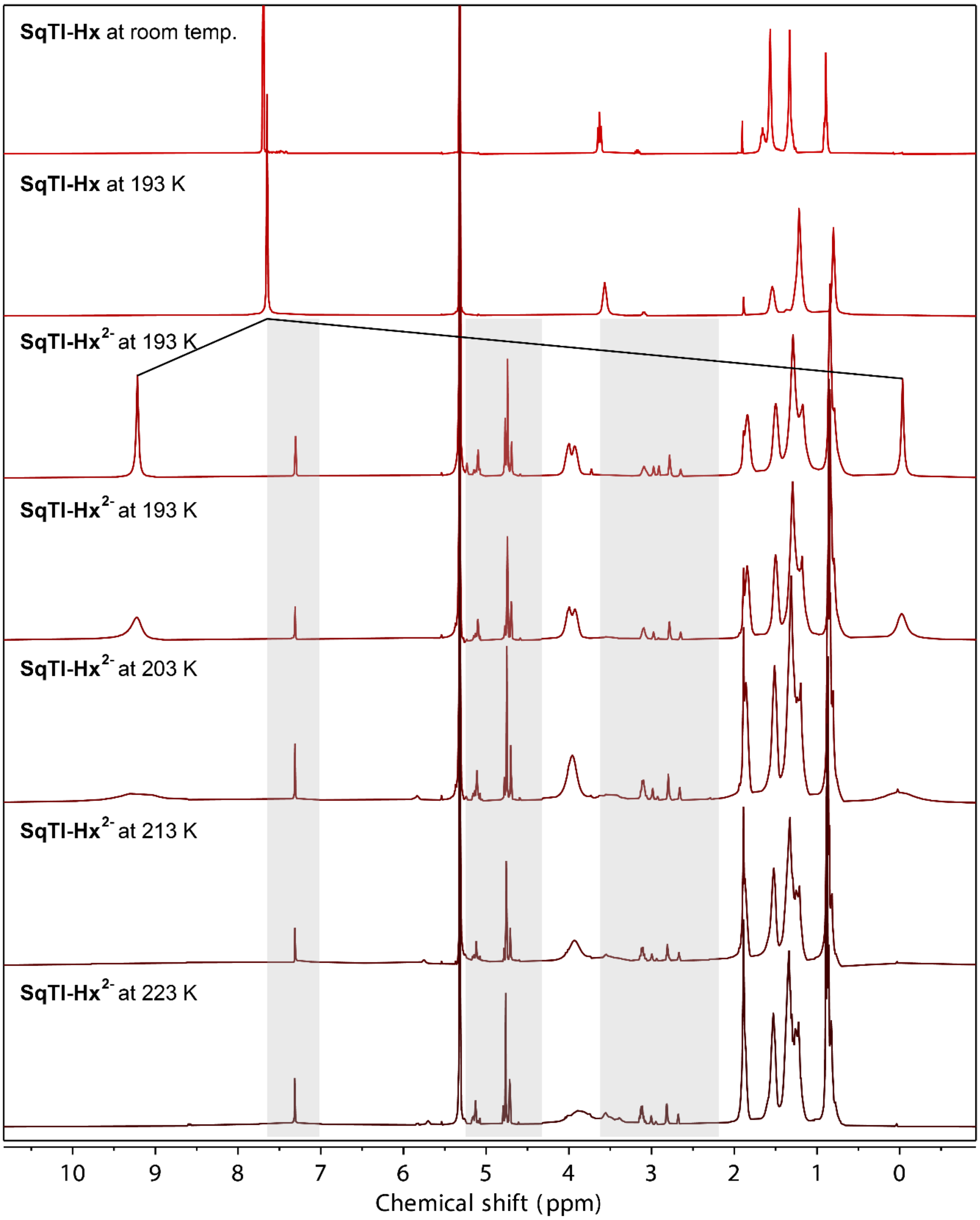

Figure S35. ^{1}H NMR (400 MHz, CD_2Cl_2) of **SqTI-Hx** at room temperature and 193 K (top) and the reduced compound **SqTI-Hx^{2-}** at 193 K (two spectra of different experiments), 203 K, 213 K, and 223 K (below). The signals shown in grey boxes do not originate from **SqTI-Hx** or **SqTI-Hx^{2-}** (see text above and Figure S36).

When increasing the temperature from 193 K to 223 K, the two signals of the phenylene-H of **SqTI-Hx^{2-}** become broader and disappear. Similar behaviour is observed for the signal at approx. 3.95 ppm (assigned to the CH_2 of the hexyl chains closest to the conjugated system) but the broadening starts at a higher temperature.

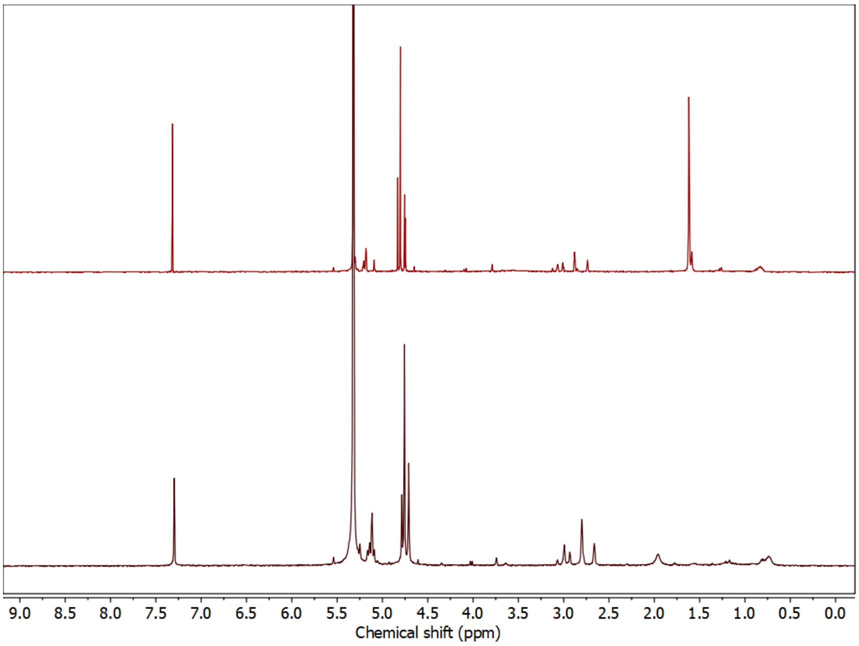

Figure S36. ^1H NMR (400 MHz, CD_2Cl_2) of diluted cobaltocene solution recorded at room temperature (top) and 193 K (bottom) without strict exclusion of air, showing that the signals in the grey boxes in Figure S35 do not originate from **SqTI-Hx** or **SqTI-Hx^{2-}**. The signal of cobaltocene itself appeared as a broad peak at -47.09 ppm at room temperature and -74.00 ppm at 193 K.

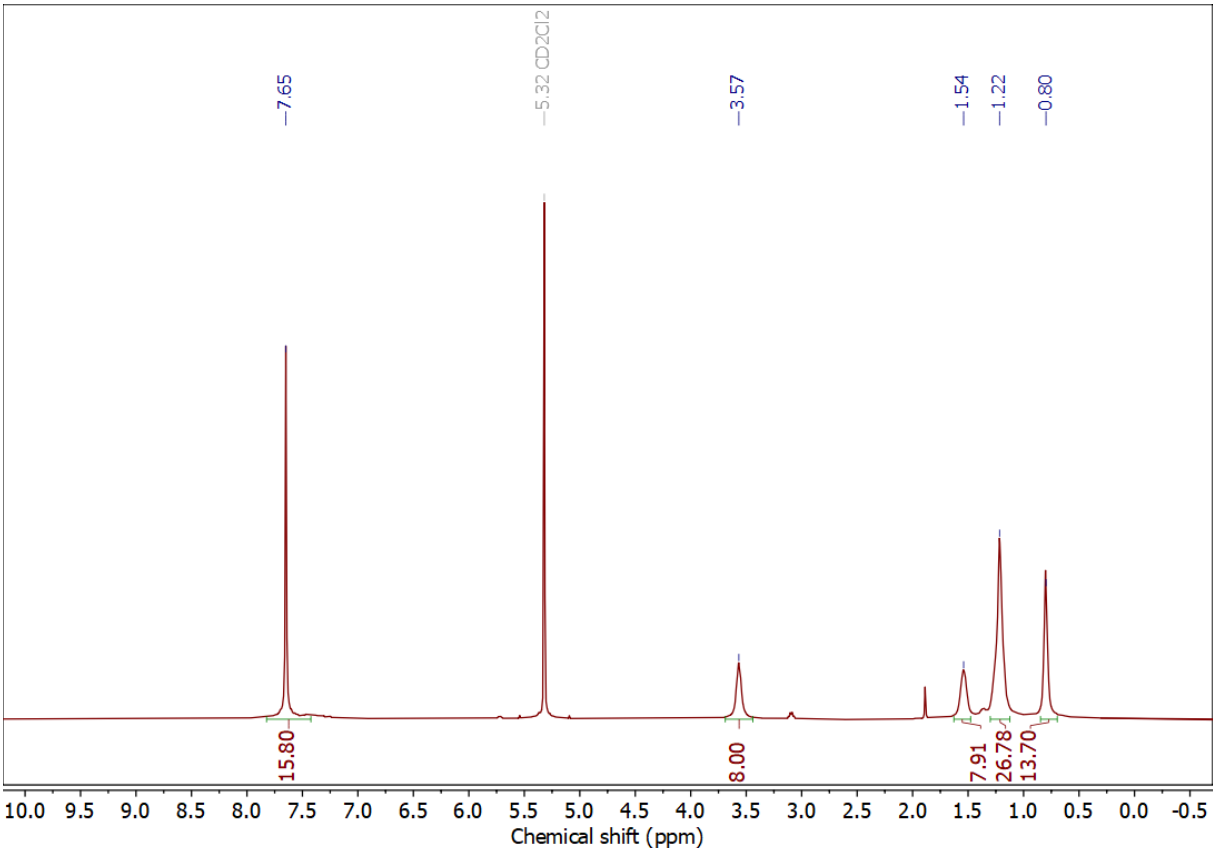

Figure S37. ^1H NMR (400 MHz, CD_2Cl_2) of **SqTI-Hx** at 193 K.

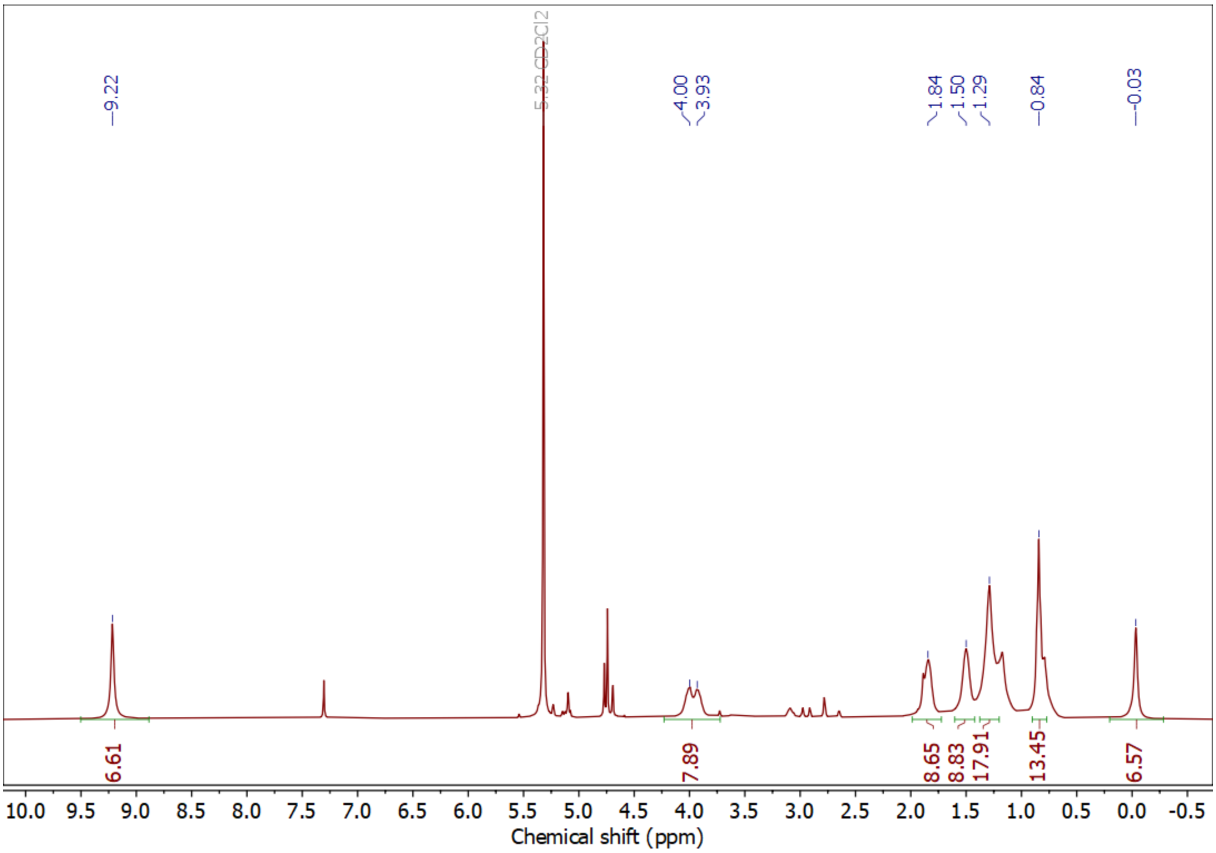

Figure S38. ^{1}H NMR (400 MHz, CD_2Cl_2) of **SqTI-Hx^{2-}** at 193 K.

7.2 Chemical reduction with sodium naphthalenide

Chemical reduction of **SqTI-Hx** with sodium naphthalenide for NMR experiments was performed under nitrogen in a glovebox. A 0.1 M stock solution of sodium naphthalenide (5 equiv., 100 µL) in dry THF-d8 (supplied in an ampule and opened inside the glovebox) was used in an attempt to form **SqTI-Hx^{4-}**, by addition (with stirring) to approx. 2 mg **SqTI-Hx** dissolved in 0.6 mL THF-d8. The resulting solution of reduced species was transferred into an NMR tube fitted with a Young valve and transported from the glovebox to the NMR spectrometer, with the first measurement taken approx. 10 min after the addition of the sodium naphthalenide solution.

The sodium naphthalenide solution was made by reacting a 0.1 M solution of naphthalene in THF-d8 with excess sodium metal for 24 h.

Figure S39 shows the NMR spectra of neutral **SqTI-Hx** in THF-d8 recorded at room temperature and 193 K at the top and the spectra of the reduced species at 193 K, 203 K, 213 K, and 223 K below. The same effects as with cobaltocene were observed; the recorded NMR signals likely correspond to the dianion (indicating incomplete reduction to the tetraanion despite the use of 5 equiv. of sodium naphthalenide), with the further reduced paramagnetic species being NMR silent.

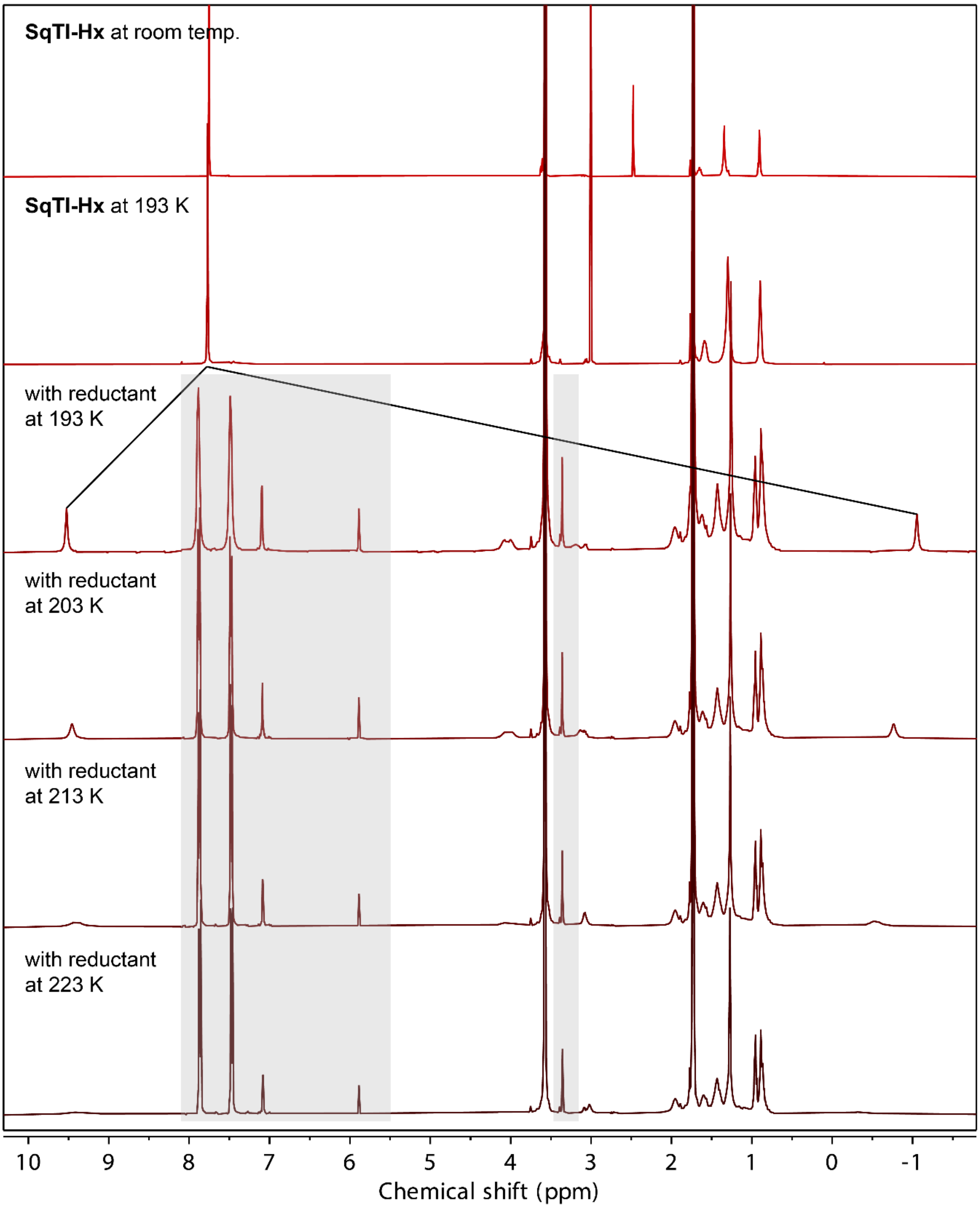

Figure S39. ^{1}H NMR (400 MHz, THF-d8) of **SqTI-Hx** at room temperature and 193 K (top) and the reduced species at 193 K, 203 K, 213 K, and 223 K (below). The signals shown in grey boxes do not originate from **SqTI-Hx** or the reduced species but from naphthalene and the sodium naphthalenide solution (see Figure S40).

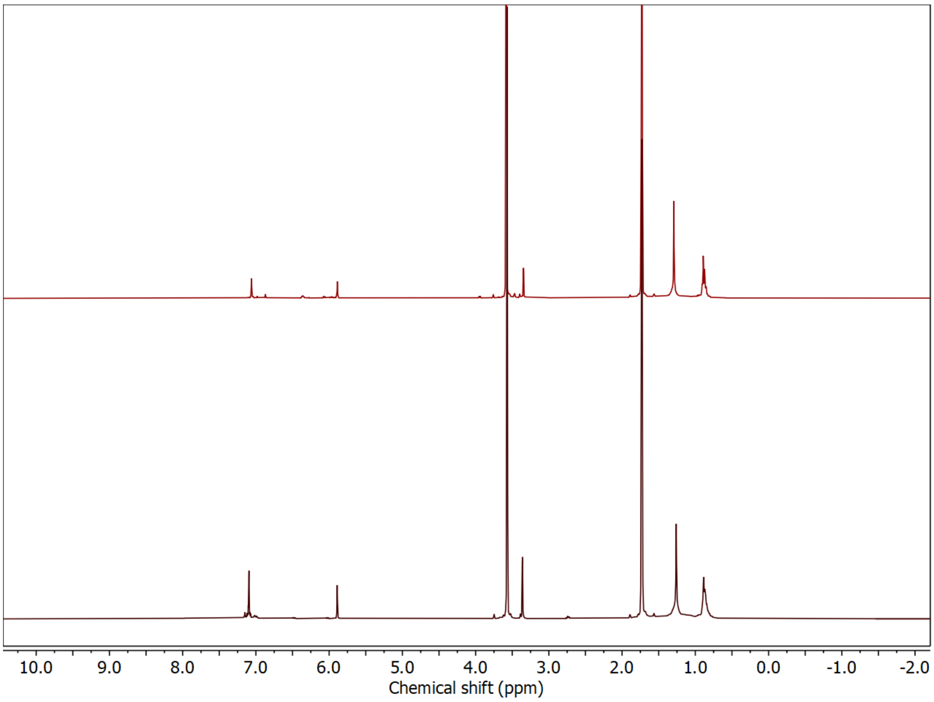

Figure S40. ^{1}H NMR (400 MHz, THF-d8) of diluted sodium naphthalenide solution recorded at room temperature (top) and 193 K (bottom) without strict exclusion of air.

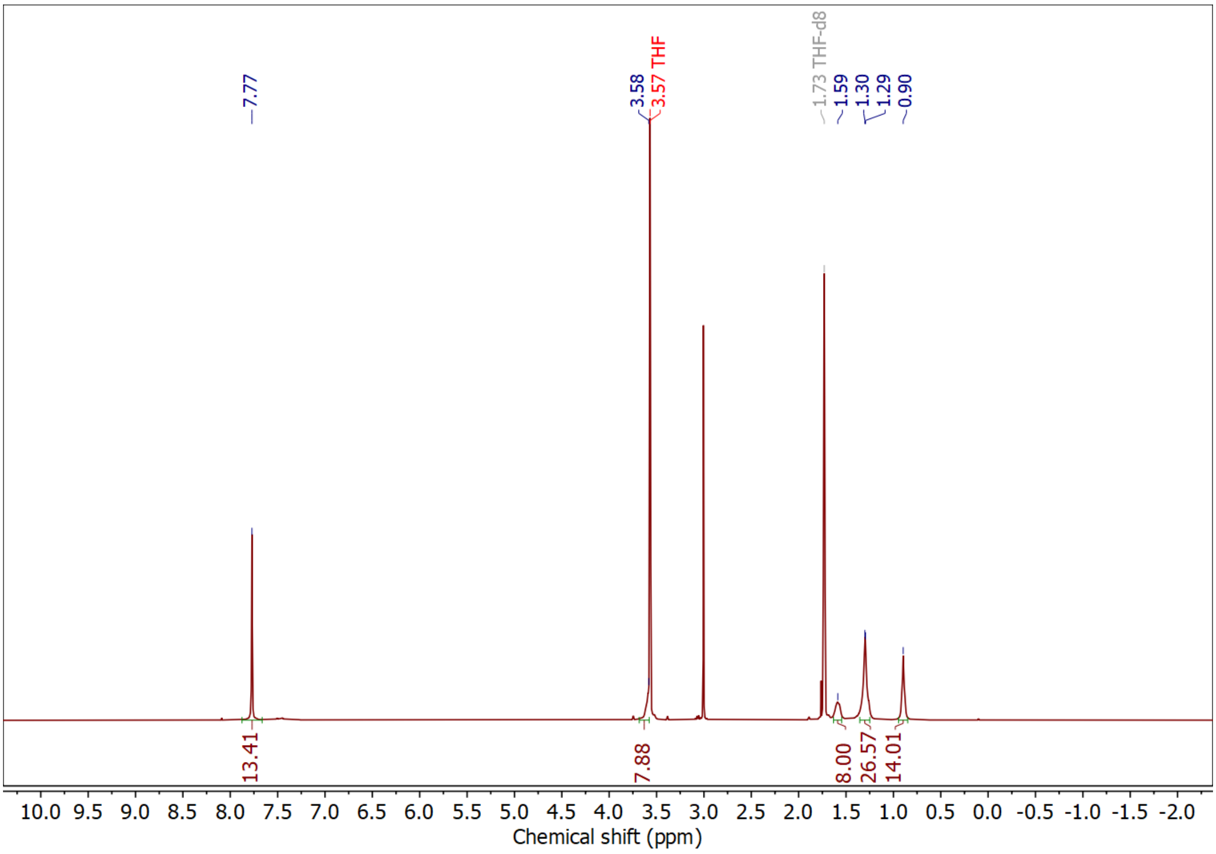

Figure S41. ^{1}H NMR (400 MHz, THF-d8) of **SqTI-Hx** at 193 K.

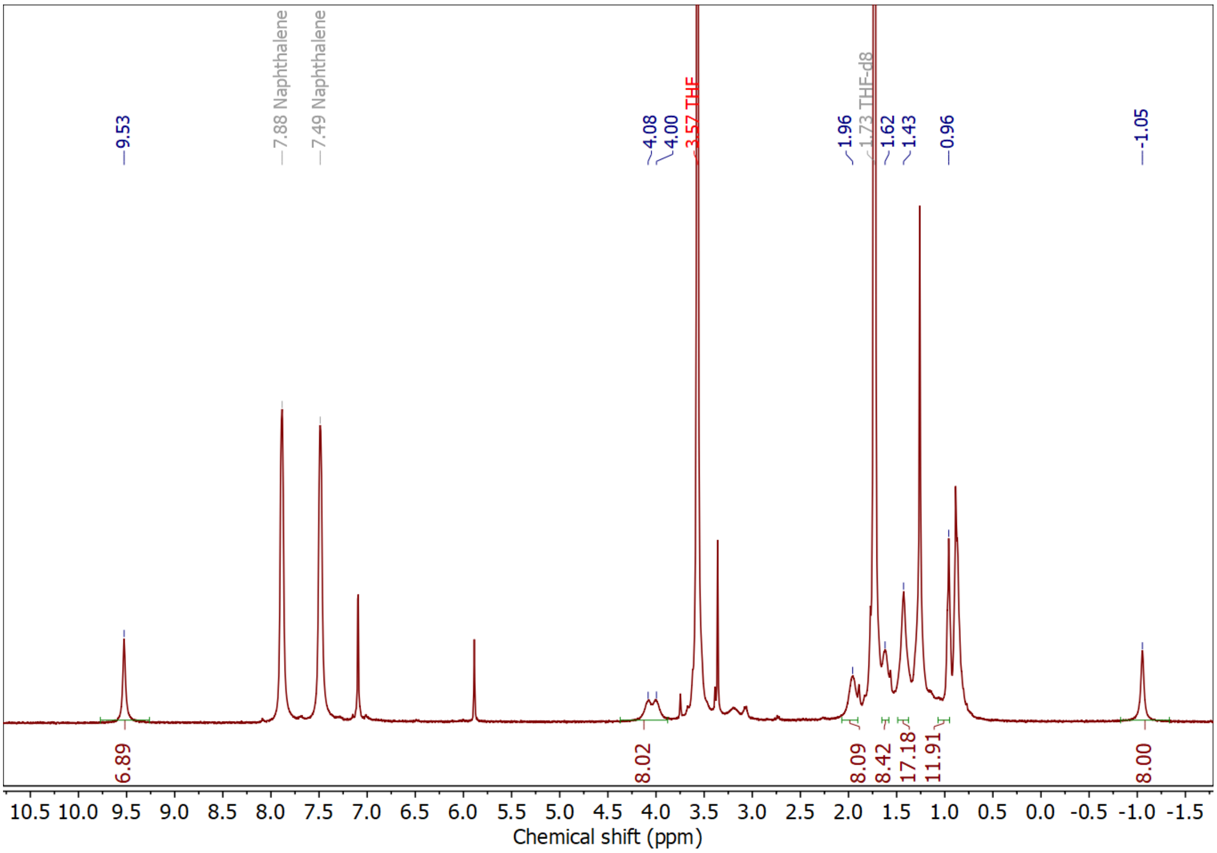

Figure S42. ^1H NMR (400 MHz, THF-d8) of **SqTI-Hx** after reduction with 5 equiv. sodium naphthalenide at 193 K.

8. UV-vis absorption and photoluminescence (PL) spectroscopy

UV-vis absorption spectra were recorded on an Agilent Cary 60 UV-Vis Spectrophotometer. All spectra were taken at room temperature under regular lab conditions with 5 µM solutions in acetonitrile (**SqTA**), $CHCl_3$ (**SqTI-R**) or H_2O (**SqNa**) at a data interval of 1 nm. For the measurements of films, 40 µL of 5 mM solution in acetonitrile (**SqTA**) or $CHCl_3$ (**SqTI-R**) were coated on cleaned glass slides using a spin coater at 1000 rpm for 1 min. The so prepared glass slides were then placed in the above-mentioned spectrophotometer.

Photoluminescence (PL) spectra of the macrocycles were acquired on an Agilent Cary Eclipse fluorescence spectrophotometer at a data interval of 1 nm. The excitation and emission slits were set to 5 nm and the detector voltage was set to ‘medium’ (600 V).

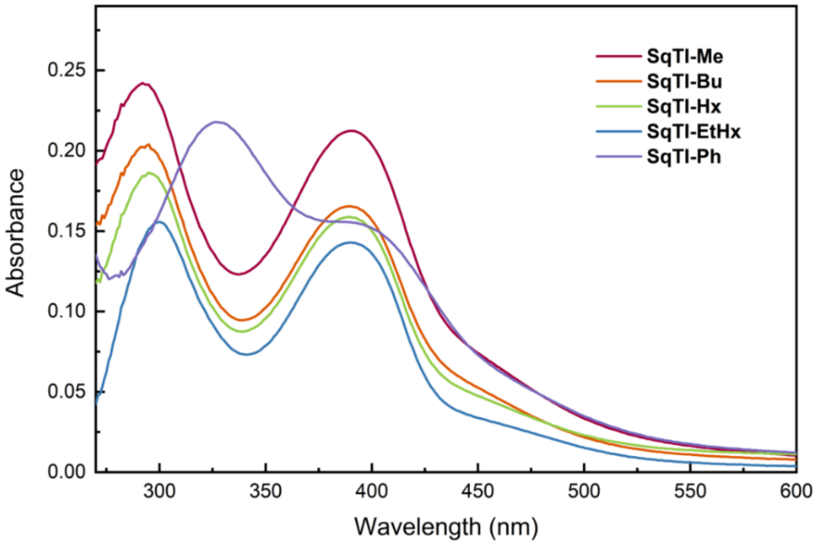

Figure S43. Film UV-vis absorption spectra of the five squarephaneic tetraimides (**SqTI-R**).

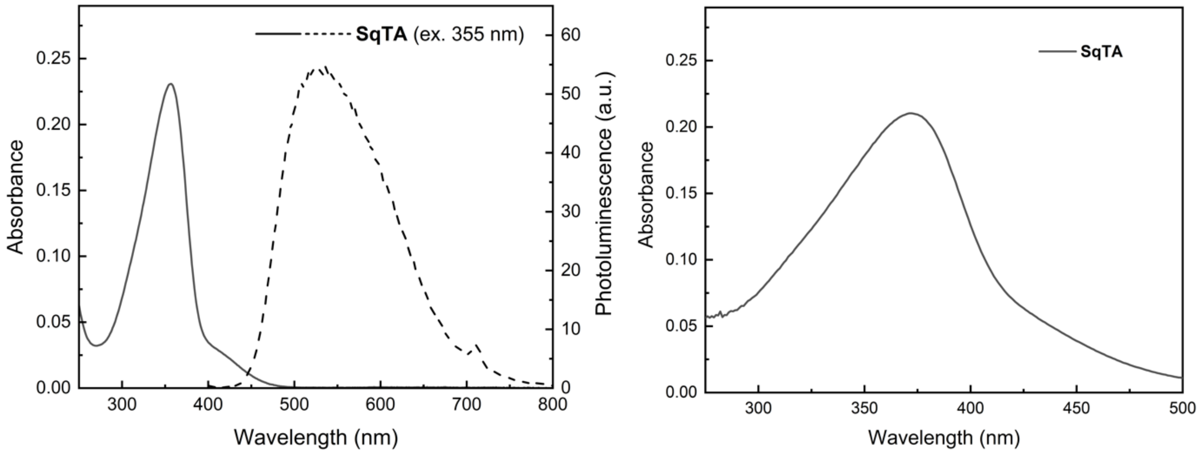

Figure S44. UV-vis absorption spectra (solid lines) and photoluminescence (PL) spectrum (dashed line) of **SqTA** in acetonitrile (left) and film (right). The excitation wavelength for recording the photoluminescence (PL) spectrum in solution is shown in brackets.

Table S5. UV-vis absorption peak wavelengths of **SqTA**, **SqTI-R**, and **SqNa** in solution (5 μM) and film. The maximum absorption wavelengths are shown in bold.

	**Absorption**				**Photoluminescence**
	Solution		Film		Solution
	$\lambda_{1st\ max}$ [nm]	$\lambda_{2nd\ max}$ [nm]	$\lambda_{1st\ max}$ [nm]	$\lambda_{2nd\ max}$ [nm]	λ_{max} [nm]
**SqTA**	**357**	–	**372**	–	526
**SqTI-Me**	385	**292**	390	**296**	542
**SqTI-Bu**	387	**294**	389	**296**	540
**SqTI-Hx**	387	**294**	389	**297**	540
**SqTI-EtHx**	387	**296**	390	**298**	545
**SqTI-Ph**	382	**323**	391	**329**	615
**SqNa**	**310**	255	n/a	n/a	–

8.1 Excitation spectra

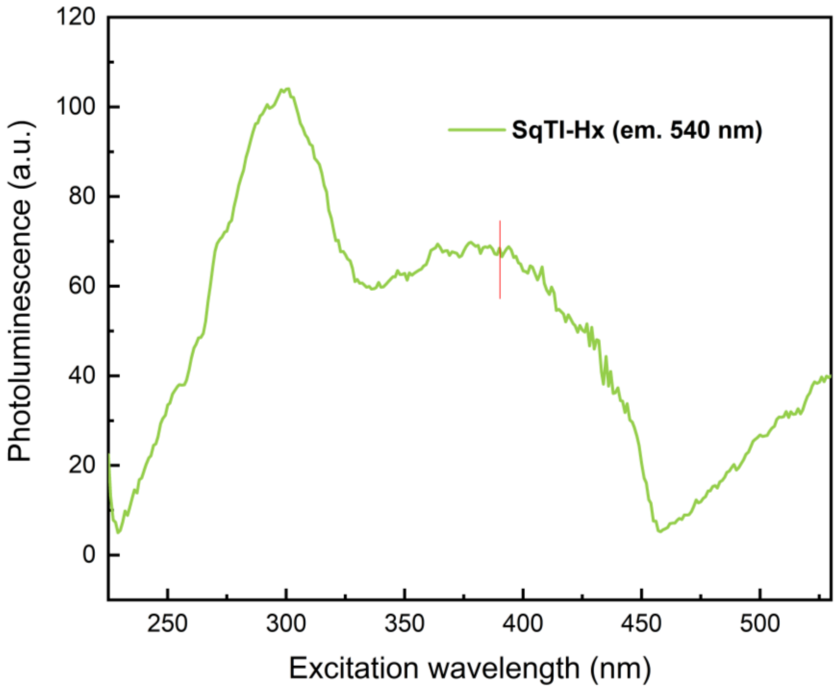

Figure S45. Excitation spectrum of **SqTI-Hx** in $CHCl_3$. The red line indicates the excitation wavelength used for recording the emission spectrum shown in Figure 6.

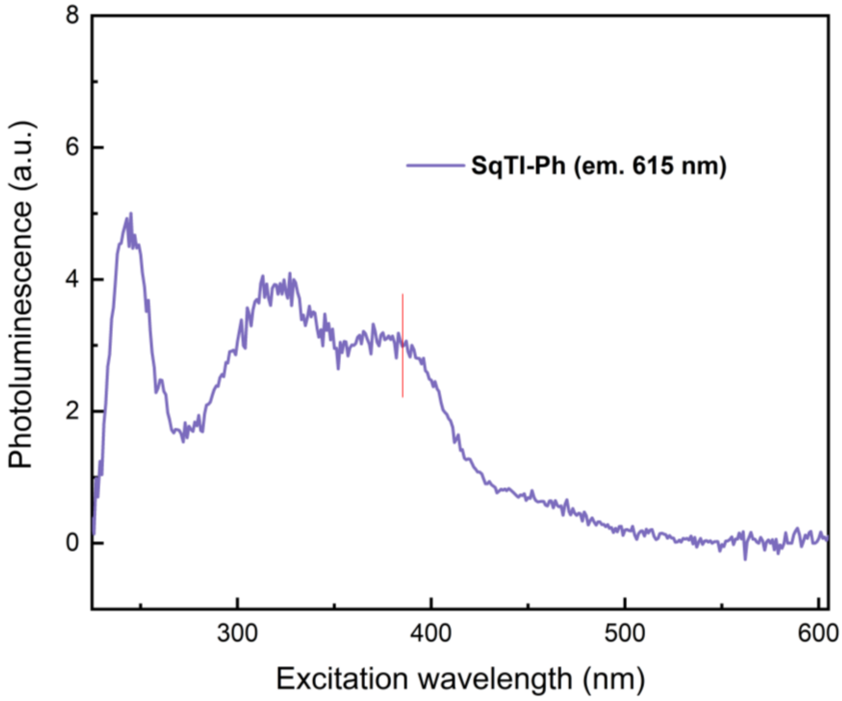

Figure S46. Excitation spectrum of **SqTI-Ph** in $CHCl_3$. The red line indicates the excitation wavelength used for recording the emission spectrum shown in Figure 6.

9. Thermogravimetric analysis (TGA)

TGA was carried out on a Mettler Toledo TGA/DSC 1LF/UMX at a heating rate of 10 °C min^{-1} under a nitrogen flow of 50 mL min^{-1}. The decomposition temperature (T_d, temperature of 5% weight loss) was determined for all macrocycles and is shown in the figures below.

SqTI-Me exhibited an initial 10% mass loss at 120 °C (Figure S48), which we attribute to residues of acetic acid (b.p. 118 °C) in the analysed batch of **SqTI-Me**. After subtracting the initial mass loss from the data, a new T_d of 498 °C was obtained, which is in good agreement with the T_d of the other squarephaneic tetraimides. Similarly, sodium squarephaneate (**SqNa**) showed an early mass loss at 66 °C (Figure S49), which corresponds to residues of methanol (b.p. 64.7 °C) in the analysed batch of **SqNa**. After adjusting the data in an analogous manner, a new T_d of 484 °C was obtained.

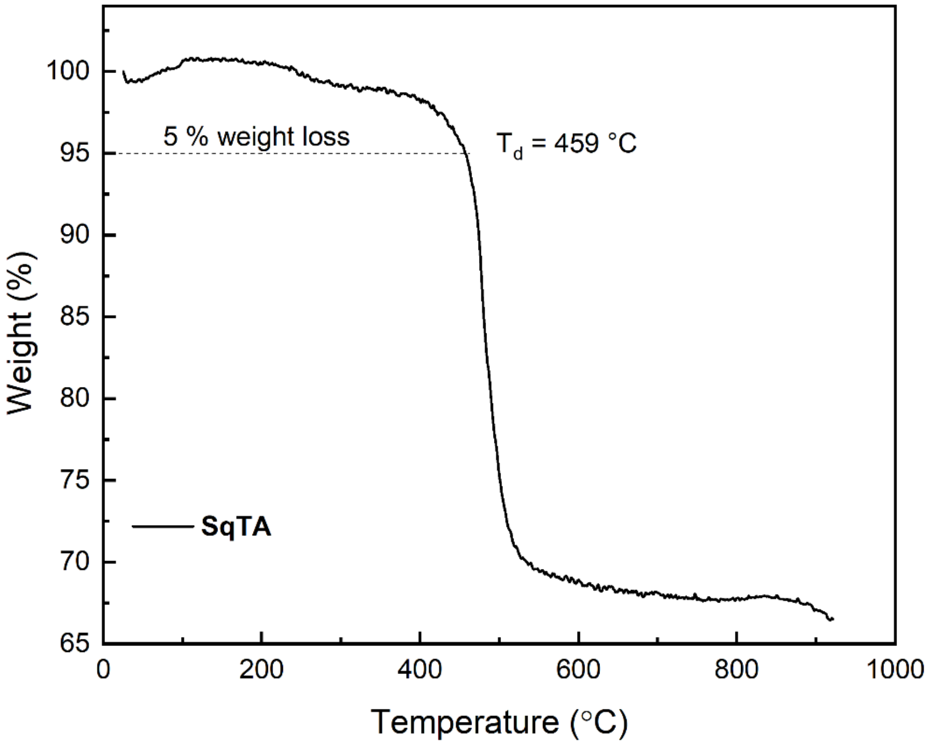

Figure S47. Thermogravimetric analysis (TGA) and decomposition temperature (T_d, temperature of 5% weight loss) of **SqTA**.

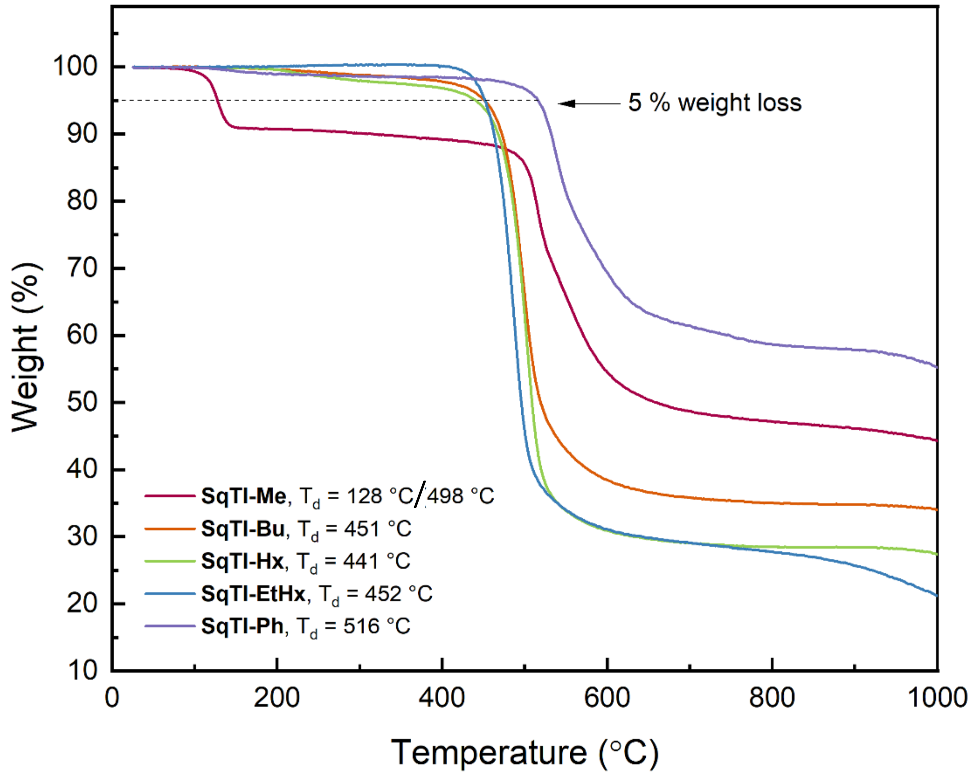

Figure S48. Thermogravimetric analysis (TGA) of **SqTl-R**; the decomposition temperatures (T_d, temperatures of 5% weight loss) and the corrected T_d for **SqTl-Me** (considering residues of acetic acid) are shown.

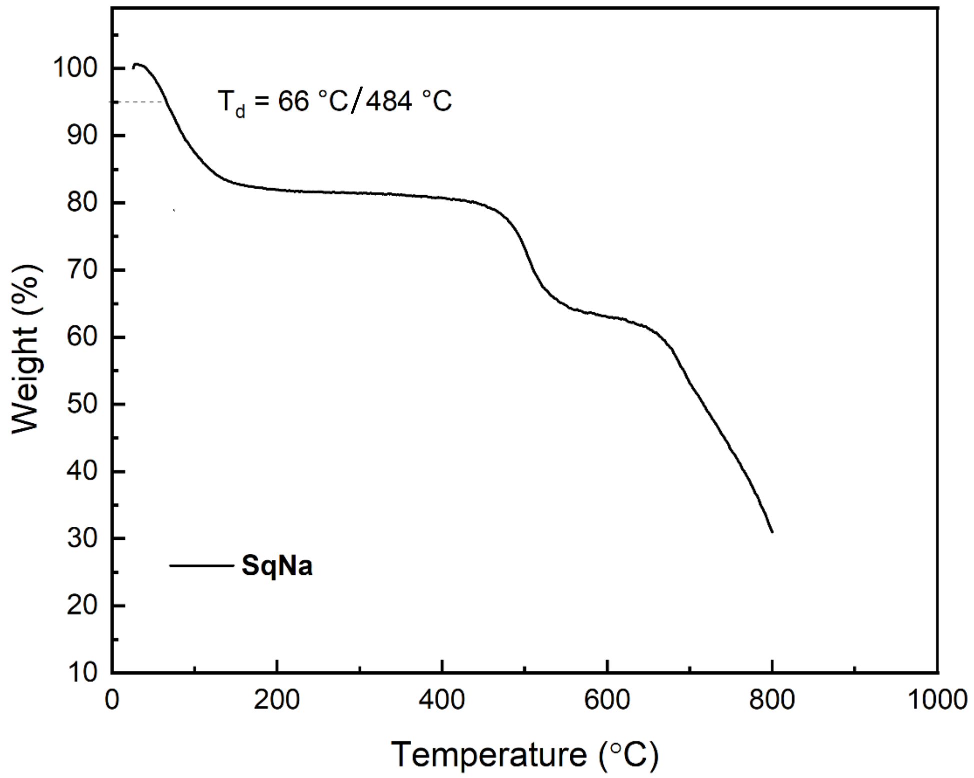

Figure S49. Thermogravimetric analysis (TGA) of **SqNa**; the decomposition temperature (T_d, temperature of 5% weight loss) and the corrected T_d (considering residues of solvent) are shown.

10. Infrared (IR) spectroscopy

Attenuated total reflectance (ATR) IR spectra were obtained on an Agilent CARY 630 FTIR spectrometer.

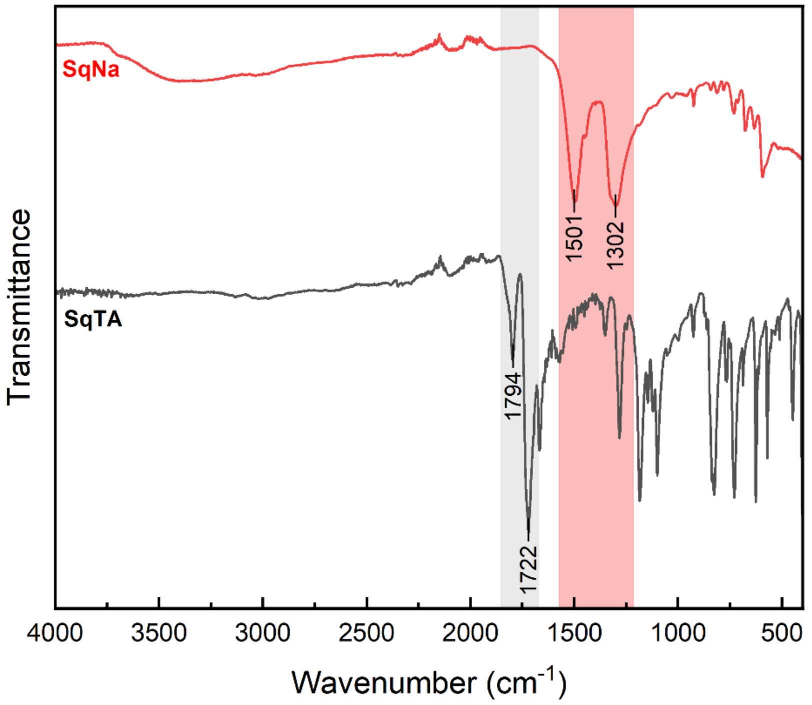

Figure S50. ATR-IR spectra of **SqNa** and **SqTA**.

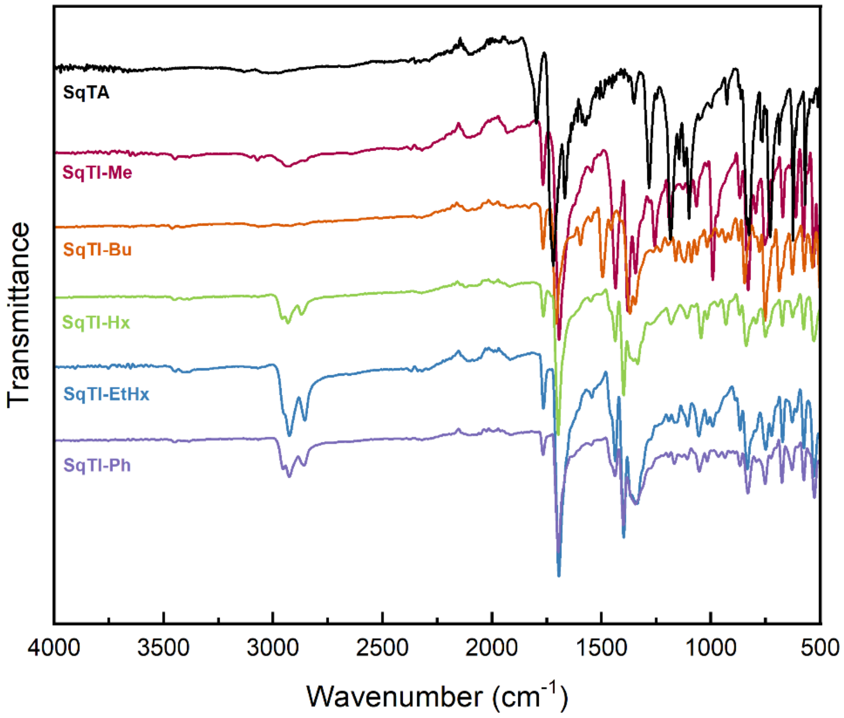

Figure S51. ATR-IR spectra of **SqTA** and **SqTI-R.**

11. References

[1] Y. Shunro, M. Kazuyuki, H. Terukiyo, *Bull. Chem. Soc. Jpn.* **1989**, *62*, 3036-3037.

[2] H. Härle, J. C. Jochims, *Chem. Ber.* **1986**, *119*, 1400-1412.

[3] T. Oakwood, C. Weisgerber, *Org. Synth.* **1944**, *24*, 16.

[4] M. Rimmele, W. Nogala, M. Seif-Eddine, M. M. Roessler, M. Heeney, F. Plasser, F. Glöcklhofer, *Org. Chem. Front.* **2021**, *8*, 4730-4745.

[5] A. Robert, P. Dechambenoit, H. Bock, F. Durola, *Can. J. Chem.* **2017**, *95*, 450-453.

[6] (a) SHELXTL v5.1, Bruker AXS, Madison, WI, **1998**; (b) G. M. Sheldrick, *Acta Crystallogr.* **2015**, *C71*, 3-8.

[7] K. A. See, M. A. Lumley, G. D. Stucky, C. P. Grey, R. Seshadri, *J. Electrochem. Soc.* **2016**, *164*, A327-A333.

[8] M. Gauthier, T. J. Carney, A. Grimaud, L. Giordano, N. Pour, H.-H. Chang, D. P. Fenning, S. F. Lux, O. Paschos, C. Bauer, F. Maglia, S. Lupart, P. Lamp, Y. Shao-Horn, *J. Phys. Chem. Lett.* **2015**, *6*, 4653-4672.

[9] (a) J. P. Perdew, K. Burke, M. Ernzerhof, *Phys. Rev. Lett.* **1996**, *77*, 3865-3868; (b) C. Adamo, V. Barone, *J. Chem. Phys.* **1999**, *110*, 6158-6170.

[10] F. Weigend, R. Ahlrichs, *Phys. Chem. Chem. Phys.* **2005**, *7*, 3297-3305.

[11] S. Grimme, J. Antony, S. Ehrlich, H. Krieg, *J. Chem. Phys.* **2010**, *132*, 154104.

[12] J. Witte, N. Mardirossian, J. B. Neaton, M. Head-Gordon, *J. Chem. Theory Comput.* **2017**, *13*, 2043-2052.

[13] A. V. Marenich, C. J. Cramer, D. G. Truhlar, *J. Phys. Chem. B* **2009**, *113*, 6378-6396.

[14] M.-H. Baik, R. A. Friesner, *J. Phys. Chem. A* **2002**, *106*, 7407-7412.

[15] E. Epifanovsky, A. T. B. Gilbert, X. Feng, J. Lee, Y. Mao, N. Mardirossian, P. Pokhilko, A. F. White, M. P. Coons, A. L. Dempwolff, Z. Gan, D. Hait, P. R. Horn, L. D. Jacobson, I. Kaliman, J. Kussmann, A. W. Lange, K. U. Lao, D. S. Levine, J. Liu, S. C. McKenzie, A. F. Morrison, K. D. Nanda, F. Plasser, D. R. Rehn, M. L. Vidal, Z.-Q. You, Y. Zhu, B. Alam, B. J. Albrecht, A. Aldossary, E. Alguire, J. H. Andersen, V. Athavale, D. Barton, K. Begam, A. Behn, N. Bellonzi, Y. A. Bernard, E. J. Berquist, H. G. A. Burton, A. Carreras, K. Carter-Fenk, R. Chakraborty, A. D. Chien, K. D. Closser, V. Cofer-Shabica, S. Dasgupta, M. d. Wergifosse, J. Deng, M. Diedenhofen, H. Do, S. Ehlert, P.-T. Fang, S. Fatehi, Q. Feng, T. Friedhoff, J. Gayvert, Q. Ge, G. Gidofalvi, M. Goldey, J. Gomes, C. E. González-Espinoza, S. Gulania, A. O. Gunina, M. W. D. Hanson-Heine, P. H. P. Harbach, A. Hauser, M. F. Herbst, M. H. Vera, M. Hodecker, Z. C. Holden, S. Houck, X. Huang, K. Hui, B. C. Huynh, M. Ivanov, Á. Jász, H. Ji, H. Jiang, B. Kaduk, S. Kähler, K. Khistyaev, J. Kim, G. Kis, P. Klunzinger, Z. Koczor-Benda, J. H. Koh, D. Kosenkov, L. Koulias, T. Kowalczyk, C. M. Krauter, K. Kue, A. Kunitsa, T. Kus, I. Ladjánszki, A. Landau, K. V. Lawler, D. Lefrancois, S. Lehtola, et al., *J. Chem. Phys.* **2021**, *155*, 084801.

[16] P. v. R. Schleyer, C. Maerker, A. Dransfeld, H. Jiao, N. J. R. van Eikema Hommes, *J. Am. Chem. Soc.* **1996**, *118*, 6317-6318.

[17] J. R. Cheeseman, G. W. Trucks, T. A. Keith, M. J. Frisch, *J. Chem. Phys.* **1996**, *104*, 5497-5509.

[18] M. J. Frisch, G. W. Trucks, H. B. Schlegel, G. E. Scuseria, M. A. Robb, J. R. Cheeseman, G. Scalmani, V. Barone, B. Mennucci, G. A. Petersson, H. Nakatsuji, M. Caricato, X. Li, H. P. Hratchian, A. F. Izmaylov, J. Bloino, G. Zheng, J. L. Sonnenberg, M. Hada, M. Ehara, K. Toyota, R. Fukuda, J. Hasegawa, M. Ishida, T. Nakajima, Y. Honda, O. Kitao, H. Nakai, T. Vreven, J. J. A. Montgomery, J. E. Peralta, F. Ogliaro, M. Bearpark, J. J. Heyd, E. Brothers, K. N. Kudin, V. N. Staroverov, T. Keith, R. Kobayashi, J. Normand, K.

Raghavachari, A. Rendell, J. C. Burant, S. S. Iyengar, J. Tomasi, M. Cossi, N. Rega, J. M. Millam, M. Klene, J. E. Knox, J. B. Cross, V. Bakken, C. Adamo, J. Jaramillo, R. Gomperts, R. E. Stratmann, O. Yazyev, A. J. Austin, R. Cammi, C. Pomelli, J. W. Ochterski, R. L. Martin, K. Morokuma, V. G. Zakrzewski, G. A. Voth, P. Salvador, J. J. Dannenberg, S. Dapprich, A. D. Daniels, O. Farkas, J. B. Foresman, J. V. Ortiz, J. Cioslowski, D. J. Fox, Wallingford, CT, **2013**.

[19] F. Plasser, F. Glöcklhofer, *Eur. J. Org. Chem.* **2021**, *2021*, 2529-2539.

[20] F. Plasser, *J. Chem. Phys.* **2020**, *152*, 084108.

[21] W. Humphrey, A. Dalke, K. Schulten, *J. Mol. Graph.* **1996**, *14*, 33-38.

[22] (a) M. Head-Gordon, A. M. Grana, D. Maurice, C. A. White, *J. Phys. Chem.* **1995**, *99*, 14261-14270; (b) F. Plasser, M. Wormit, A. Dreuw, *J. Chem. Phys.* **2014**, *141*, 024106.
